# A map of tumor–host interactions in glioma at single-cell resolution

**DOI:** 10.1093/gigascience/giaa109

**Published:** 2020-10-14

**Authors:** Francesca Pia Caruso, Luciano Garofano, Fulvio D'Angelo, Kai Yu, Fuchou Tang, Jinzhou Yuan, Jing Zhang, Luigi Cerulo, Stefano M Pagnotta, Davide Bedognetti, Peter A Sims, Mario Suvà, Xiao-Dong Su, Anna Lasorella, Antonio Iavarone, Michele Ceccarelli

**Affiliations:** Department of Electrical Engineering and Information Technology (DIETI), University of Naples “Federico II”, Via Claudio 21, 80128 Naples, Italy; Bioinformatics Lab, BIOGEM, Via Camporeale, 83031 Ariano Irpino, Italy; Department of Electrical Engineering and Information Technology (DIETI), University of Naples “Federico II”, Via Claudio 21, 80128 Naples, Italy; Institute for Cancer Genetics, Columbia University, 1130 St Nicholas Ave, New York, NY 10032, USA; Bioinformatics Lab, BIOGEM, Via Camporeale, 83031 Ariano Irpino, Italy; Institute for Cancer Genetics, Columbia University, 1130 St Nicholas Ave, New York, NY 10032, USA; Biomedical Pioneering Innovation Center (BIOPIC), School of Life Sciences, Peking University, 5 Yiheyuan Rd, Haidian District, 100871 Beijing, China; Biomedical Pioneering Innovation Center (BIOPIC), School of Life Sciences, Peking University, 5 Yiheyuan Rd, Haidian District, 100871 Beijing, China; Department of Science and Technologies, Università degli Studi del Sannio, Via de Sanctis, 82100 Benevento, Italy; Cancer Program, Sidra Medicine, Al Luqta Street, Zone 52, Education City, 26999, Doha Qatar; Institute for Cancer Genetics, Columbia University, 1130 St Nicholas Ave, New York, NY 10032, USA; Bioinformatics Lab, BIOGEM, Via Camporeale, 83031 Ariano Irpino, Italy; Department of Science and Technologies, Università degli Studi del Sannio, Via de Sanctis, 82100 Benevento, Italy; Department of Science and Technologies, Università degli Studi del Sannio, Via de Sanctis, 82100 Benevento, Italy; Cancer Program, Sidra Medicine, Al Luqta Street, Zone 52, Education City, 26999, Doha Qatar; Department of Internal Medicine and Medical Specialties (Di.M.I.), University of Genoa, Viale Benedetto XV 10, 16132 Genoa, Italy; Department of Systems Biology, Columbia University Irving Medical Center, 1130 St Nicholas Ave, New York , NY 10032, USA; Department of Biochemistry and Molecular Biophysics, Columbia University Irving Medical Center, 1130 St Nicholas Ave, New York, NY 10032, USA; Department of Pathology and Center for Cancer Research, Massachusetts General Hospital and Harvard Medical School, 55 Fruit St, Boston, MA 02114, USA; Broad Institute of Harvard and MIT, 415 Main St, Cambridge, MA 02142, USA; Biomedical Pioneering Innovation Center (BIOPIC), School of Life Sciences, Peking University, 5 Yiheyuan Rd, Haidian District, 100871 Beijing, China; Institute for Cancer Genetics, Columbia University, 1130 St Nicholas Ave, New York, NY 10032, USA; Department of Pathology and Cell Biology, Columbia University Medical Center, 1130 St Nicholas Ave, New York , NY 10032 USA; Institute for Cancer Genetics, Columbia University, 1130 St Nicholas Ave, New York, NY 10032, USA; Department of Pathology and Cell Biology, Columbia University Medical Center, 1130 St Nicholas Ave, New York , NY 10032 USA; Department of Neurology, Columbia University Medical Center, 1130 St Nicholas Ave, New York, NY 10032, USA; Department of Electrical Engineering and Information Technology (DIETI), University of Naples “Federico II”, Via Claudio 21, 80128 Naples, Italy; Bioinformatics Lab, BIOGEM, Via Camporeale, 83031 Ariano Irpino, Italy

**Keywords:** Brain Tumors, Single Cells, Ligand-receptor signalling, Tumor Microenvironment

## Abstract

**Background:**

Single-cell RNA sequencing is the reference technique for characterizing the heterogeneity of the tumor microenvironment. The composition of the various cell types making up the microenvironment can significantly affect the way in which the immune system activates cancer rejection mechanisms. Understanding the cross-talk signals between immune cells and cancer cells is of fundamental importance for the identification of immuno-oncology therapeutic targets.

**Results:**

We present a novel method, single-cell Tumor–Host Interaction tool (scTHI), to identify significantly activated ligand–receptor interactions across clusters of cells from single-cell RNA sequencing data. We apply our approach to uncover the ligand–receptor interactions in glioma using 6 publicly available human glioma datasets encompassing 57,060 gene expression profiles from 71 patients. By leveraging this large-scale collection we show that unexpected cross-talk partners are highly conserved across different datasets in the majority of the tumor samples. This suggests that shared cross-talk mechanisms exist in glioma.

**Conclusions:**

Our results provide a complete map of the active tumor–host interaction pairs in glioma that can be therapeutically exploited to reduce the immunosuppressive action of the microenvironment in brain tumor.

## Background

The interaction between malignant cells and their microenvironment influences tumor growth and progression because the immune system can eliminate cancer cells that present neoantigens recognized by receptors of the adaptive immune system or express ligands for activating receptors on innate immune cells [1]. The composition of the various cell types making up the microenvironment can significantly affect the way in which the immune system activates cancer rejection mechanisms [2–4] and influences the response to immune therapies [5, 6]. Therefore, the elucidation of the tumor–host interaction mechanisms plays a crucial role in the understanding of tumor growth and evolution [7, 8] and in the identification of immuno-oncology therapeutic targets [9]. Immune checkpoint inhibitor (ICI) therapies are aimed at targeting specific cell–cell interactions between programmed cell death protein 1 (PD1) and programmed death–ligand 1 (PD-L1) or cytotoxic T lymphocyte–associated protein 4 (CTLA-4) and B7-1/B7-2 [10]. The identification of novel interactions that characterize tumor types and shape the immune response also has important clinical implications and can help to better stratify patients [2] and predict response to ICI [11].

Single-cell RNA sequencing is the reference technology for the quantification and phenotyping of the tumor microenvironment at high resolution [12, 13], enabling measurement of the composition of individual immune/stromal compartments making up the microenvironment. This technique can also be used for a better elucidation of the tumor–host signaling mechanisms [14] and the identification of tissue-specific interactions at an unprecedented spatially resolved level of detail [15].

Gliomas are characterized by the worst survival among brain tumor malignant neoplasms [6]. In particular glioblastoma (GBM; grade IV glioma) is the most frequent type of primary brain tumor, having a median survival <15 months [16]. In glioma, higher mutational load is associated with increased tumor aggressiveness [2]. Myeloid-derived cells, mostly blood-derived macrophages and resident microglia, are the most prevalent immune compartments observed in the microenvironment of GBM [17–19], inhibiting a productive anti-tumor immunity in GBM and excluding T lymphocytes [20]. The elucidation of the active ligand–receptor (L–R) interactions in the cross-talk between tumor cells and their microenvironment can help to identify the mechanisms that the transformed cells in glioma use to recruit this immunosuppressive microenvironment and to discover novel therapeutic targets.

Here we exploit single-cell data developing single-cell Tumor–Host Interaction (scTHI), a novel algorithm and tool to identify the L–R pairs that modulate the tumor microenvironment cross-talk in glioma. scTHI is based on the hypothesis that when patterns of interaction are active, they are also simultaneously and highly expressed in homogeneous cell populations. We also model the autocrine and paracrine signaling effects of L–R partners [21]. Interestingly, by assembling the largest collection of single-cell datasets available to date, we show that unexpected cross-talk partners are highly conserved across different datasets in the majority of the tumor samples. This suggests that shared cross-talk mechanisms exist in glioma. Our results provide a complete map of the active tumor–host interaction pairs in glioma that can be therapeutically exploited to reduce the immunosuppressive action of the microenvironment in brain tumor.

## Data Description

### Single-cell datasets

Glioma single-cell gene expression profiles were collected from 6 different datasets of glioma (Table 1). We obtained a subset of isocitrate dehydrogenase (IDH)-mutant gliomas including 10,688 cells from 16 patients from 2 different studies [22, 23]. Later, we refer to this subset of cells as a unique dataset. High-grade glioma profiles were collected from 3 distinct studies: Darmanis et al. profiled 3,589 cells from 4 patients [24]; Yuan et al. profiled ∼29,000 cells from 10 patients augmented with 2 novel specimens (PJ052 and PJ053) following library construction and sequencing described in [17]; and Neftel et al. profiled 7,930 cells from 28 patients [25]. We also considered another dataset of 5,603 single-cell profiles derived from both patients with low-grade and high-grade glioma (n = 13) [26]. Overall, we collected gene expression profiles of 57,060 cells for a total of 71 patients with glioma. The cohort is composed of several tumor histologies, including 7 oligodendrogliomas (4,753 cells), 2 oligodendroastrocytomas (612 cells), 11 astrocytomas (9,421 cells), and 50 GBMs (42,274 cells). Gene expression profiles were processed independently for each dataset. The transcripts-per-million–normalized data of the Tirosh et al., Venteicher et al., and Neftel et al. [22, 23, 25] datasets were downloaded from the GEO repositories under accession numbers GSE70630, GSE89567, GSE131928, respectively. Gene expression profiles from Yuan et al. and Darmanis et al. [17, 24] were downloaded from GEO repositories under accession numbers GSE103224 and GSE84465, respectively. Meanwhile, raw data from Yu et al. [26] were obtained from the authors. The last 3 datasets were further processed applying a library size normalization and logarithmic transformation. Moreover, to reduce the drop-out effects, data matrices were also imputed with a Markov affinity-based graph approach [27]. When the specific information to distinguish malignant cells from non-tumor cells was not available, we analyzed the chromosomal aberrations in each individual cell by averaging expression level along genomic locations as performed by InferCNV [28]. The chromosomal landscape of inferred copy number variation (CNV) allows us to identify non-transformed cells, i.e., cells that did not harbor the typical chromosomal alterations observed in glioma. Altogether we identified 45,550 malignant cells and 11,510 non-malignant cells among datasets.

**Table 1:** Overview of collected datasets

Dataset	GEO Accession	No. tumor cells	No. non-tumor cells	No. patients	Protocol
Tirosh et al. [22]	GSE70630	4,045	302	6	Smart-seq2
Venteicher et al. [23]	GSE89567	5,284	1,057	10	Smart-seq2
Darmanis et al. [24]	GSE84465	1,091	2,498	4	Smart-seq2
Yuan et al. [17]	GSE103224	25,056	4,194	10	Proprietary microwell platform
Neftel et al. [25]	GSE131928	6,863	1,067	28	Smart-seq2
Yu et al. [26]	GSE117891	3,211	2,392	13	STRT-seq
Total		45,550	11,510	71	

STRT-seq: single-cell tagged reverse transcription sequencing.

### Ligand–receptor collection

To identify potential tumor–host interactions we collected a list of 2,548 pairs of ligands and receptors (Table S1) curating publicly available resources [21, 29]. The curated list is composed of known and novel literature-supported interactions and includes both heteromeric and monomeric ligands/receptors mainly related to chemokine, cytokine, growth factor, integrin, transforming growth factor (TGF) and tumor necrosis factor (TNF) family members, semaphorins, ephrins, Wnt, and Notch signaling. The table of interactions is released in the scTHI Bioconductor package.

### Immune cell type signatures

Cell-type–specific signatures and markers were used to infer the cellular identity of non-malignant cell subpopulations. For this purpose, we generated a curated list of 295 signatures of the immune and central nervous systems integrating data from various sources (Table S2). In particular we merged a manual collection of marker genes with a set of signatures available from public databases and published studies, including (i) a compendium of 64 human cell type signatures including lymphoid, myeloid, stromal, tissue-specific, and stem cells, collected from FANTOM5, ENCODE, Blueprint, and Gene Expression Omnibus (GEO) data portals; (ii) a set of markers for 30 immune cell types, including myeloid and lymphoid subpopulations identified from peripheral blood mononuclear cells [30]; (iii) a set of central nervous system cell signatures including astrocytes, neurons, oligodendrocytes, microglia, and endothelial cells [31]; (iv) a set of 53 signatures corresponding to 26 different cell types [32–35]; and (v) 2 gene expression programs related to microglia and bone marrow–derived macrophages in gliomas [23]. All 295 signatures are released in the scTHI tool.

## Analyses

We present scTHI, an R/Bioconductor package to discover L–R interactions in single cells. There have been several attempts at scoring such pairs that are mainly based on the mean expression of the gene pairs across cell populations. One recent approach is reported in Kumar et al. [14], in which the authors score interactions by calculating the product of mean receptor expression and mean ligand expression in the respective cell types under examination. The significance of the interaction is evaluated through a 1-sided Wilcoxon rank-sum between the median interaction score across samples. This idea is similar to the original approach reported by the authors of CellPhoneDB [29], where the mean expression of the gene pair is considered with the constraint that only receptors and ligands expressed in >10% of the cells in the analyzed cluster are selected. The significance of the interaction is then evaluated using a random permutation of the samples. Likewise, the approach proposed by Wang et al. [36] evaluated the expression levels of both ligands and receptors and only highly expressed or differentially expressed genes were selected to find significant L–R interaction. To test whether an interaction pair was highly expressed in 2 populations, Joost et al. [37] used a random sampling approach, selecting only pairs with an expression level above a baseline threshold with a corrected *P* < 0.01. Instead, Halpern et al. [38] computed an enrichment for each interaction based on the *z*-score of the mean expression of every L–R pair tested.

**Figure 1:**
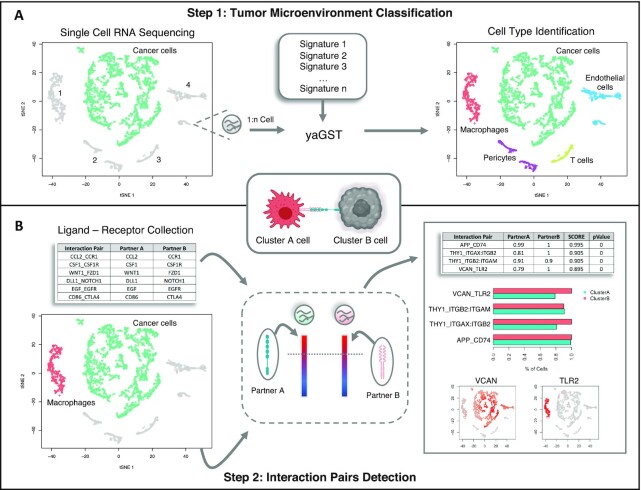
scTHI workflow. Description of scTHI functionalities. (A) In the first step a single sample enrichment analysis based on the Mann-Witney-Wilcoxon gene set test (mww-GST) [39] with a collection of 295 gene immune and stromal signatures is used to nominate the identity of cells of the microenvironment. (B) In the second step, given 2 clusters of cells, the most significant L–R interactions are identified by assigning a score to all 2,548 collected pairs of ligands and receptors.

The basic workflow of scTHI is presented in Fig. 1. First, we perform a cell-specific identification using the gene set enrichment Mann-Whitney-Wilcoxon gene set test (mww-GST) [39] based on a collection of 295 gene immune and stromal signatures. We adopt mww-GST because it has been proven to perform better than other enrichment analysis methods in situations of weak and noisy signals and therefore can be used in single-cell scenarios with the presence of low detection efficiency and drop-out phenomena. The second step of the pipeline is the scoring of the candidate L–R pairs using the procedure described in the Methods section. Briefly, the method computes for each cluster the percentage of cells where the L–R partners are ranked at the top 20% (this is a tunable parameter). The score is the mean between the 2 percentages and it prioritizes L–R with paracrine activation, removing from the score the autocrine effects (see Methods). The significance of the score is computed by a bootstrap-generated null distribution obtained by randomly shuffling the input data.

Most of the aforementioned methods use mean expression as a measure to detect whether an interaction exists. They do not consider that single-cell RNA-seq expression can be significantly influenced by drop-out effect and the average introduces bias in L–R detection. Differently from these methods, the scTHI score is based on the percentage of cells in 2 clusters where the expression of the L–R genes is ranked at the top of the expression profile of every cell of the cluster. The choice to use as score the percentage of cells expressing a top-rank L–R pair gives priority to those pairs that are more uniformly expressed in 2 clusters of cells. Overall, this allows us to discard interaction pairs for which 1 of the 2 members is highly expressed and the other is not, which instead would be detected using a score based on the average of the expression. To show how scTHI reduces the number of false-positive results with respect to methods based just on the gene expression average such as iTALK [36] we can consider the simple example in Fig. S1A, where we have a pair MMP7-ERBB4, with one of the partners poorly or not expressed at all in the cells. This pair is discarded by scTHI; however, the mean expression of both, 5.37, is comparable to that of most other reliable pairs such as the pair APP-FPR2 (score = 0.995, mean expression = 6.7) or BGN-TLR4 (score = 0.960, mean expression = 5.21) in the same patient reported in the figure. Similarly, in cases involving protein complexes, scTHI ensures the selection of only L–R pairs for which both partners of the complex are expressed at the top in the majority of the cells in 1 of the clusters. Fig. S1B shows the expression of the pair formed by the ITGB1:ITGA9 complex and the *SPP1*gene in patient MGH42; the ITGB1:ITGA9 complex should be expressed by cancer cells. However only 1 of the 2 members of the complex is highly expressed in the tumor cluster (*ITGB1*) while the expression of the other partner (*ITGA9*) is almost undetected. This pair has a scTHI score of 0 and is discarded. However, it has a global expression level (mean expression = 4.36) similar to that of other correctly expressed pairs, e.g., THY1_ITGAX:ITGB2 (mean expression 4.59 and scTHI score = 0.7).

Finally, differently from iTALK and cellPhoneDB, scTHI score explicitly models paracrine and autocrine effects. Because we are particularly interested in paracrine effects, our score penalizes L–R pairs in which both partners are highly expressed in the same cell. In the paracrine mode, scTHI returns only those pairs for which partner A is expressed only in 1 of the clusters and partner B is expressed in the other cluster. Meanwhile, in the autocrine mode scTHI returns all pairs for which the ligand and/or receptor are expressed at approximately the same level in both clusters (an example of the 2 different detection modes is reported in Fig. S1C).

### scTHI is able to recover validated interactions from single-cell data

Many putative L–R interactions could be identified on the basis of quantitative gene expression evaluation. However, only those occurring among cells spatially close to each other could have a real biological functionality. Goltsev et al. observed that the cellular neighborhood has a profound effect on the expression of protein receptors on immune cells [40], highlighting that the spatial resolution of infiltrating immune cells and the cancer cells plays a key role in defining tumor heterogeneity. Given these assumptions, we evaluated whether scTHI is able to detect interactions occurring between clusters of cells that are spatially close. For this reason, we used the high-quality CITE-seq dataset described in Govek et al., where the spatial architecture of murine splenic cells was resolved [15]. First, we used scTHI to identify all cell types composing the murine spleen described by Govek et al., including T cells (cytotoxic, memory, naive, regulatory, and helper), B cells (follicular, naive, and switched memory), dendritic cells (conventional and plasmacytoid), red-pulp macrophages, monocyte-derived macrophages, neutrophils, plasma cells, erythrocytes, and erythroid progenitors. We used the signatures in scTHI (generated for human) converted to their mouse orthologs. We asked whether scTHI was able to detect interactions occurring between clusters of spatially close cells. Govek et al. [15] used CODEX to validate some important L–R interactions occurring among red-pulp macrophages and monocyte-derived macrophages (C1q-Lrp1), red-pulp macrophages and neutrophils (Hebp1-Fpr2), and monocyte-derived macrophages and neutrophils (Anxa1-Fpr1). They identified these interactions on the basis of the proximity of the corresponding cells expressing ligands and receptors in both the CITE-seq and CODEX data. Interestingly, scTHI detected the validated interactions among the top 10 highest scored (*P* = 0.0277), without any spatial information, as shown in Fig. S2. This highlights how our approach, which scored pairs on the basis of rank expression values, is robust and accurate in the identification of relevant L–R interactions.

### Map of non-tumor cells in glioma

**Figure 2:**
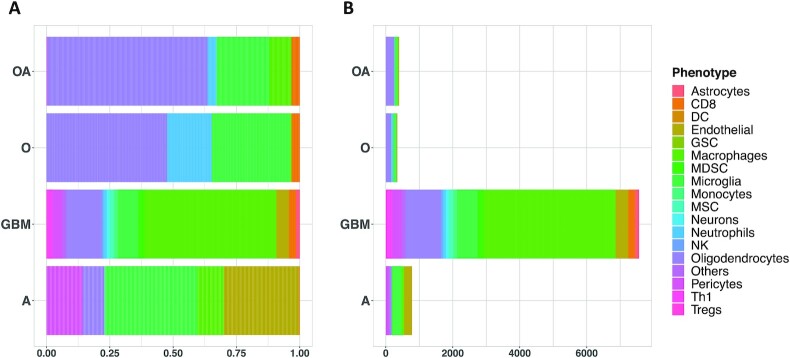
Tumor microenvironment cell type classification in glioma. The bar plots show the relative percentage (A) and the number of cells (B) of each cell type identified in the microenvironment of the main histological subtypes of glioma. DC: dendritic cell; GSC: glioma stem cell; MDSC: myeloid-derived suppressor cell; MSC: mesechymal stem cell; NK: natural kiler cell; Treg: regulatory T cell.

Gliomas are primary brain tumors characterized by high levels of intratumor heterogeneity, and, despite numerous research advances, the difference in tumor microenvironment composition is still not well understood [41]. We collected a single-cell glioma dataset integrating 6 published studies. This allowed us to comprehensively evaluate the composition of the tumor microenvironment, spanning different molecular and histological subtypes of glioma. Overall, we have 45,550 malignant cells and 11,510 non-malignant cells among datasets. We classified all non-malignant cells using scTHI; however, below we report the percentages of specific cell compartments computed using the datasets where the cells did not undergo any gating or selection strategy. Classification of the non-malignant cells (Table S3) showed that the most frequent cells in the glioma microenvironment were myeloid cells (∼57%), divided in macrophages (∼45%) and microglia (∼12%), followed by glial cells (∼19%), vascular cells (∼11%), CD8-positive (CD8^+^) T cells (∼4%), and a few subpopulations of other cell types including natural killer (NK), neutrophils, dendritic cells, monocytes, mesenchymal stem cells, and others (∼9%). As expected, grade IV glioma (GBM) showed the highest percentage of macrophages in their microenvironment (∼52% macrophages and ∼8% microglia) compared with other histological subtypes (astrocytoma: macrophages = ∼10% and microglia = ∼36%; oligo-astrocytoma: macrophages = ∼9% and microglia = ∼21%; oligodendroglioma: macrophages = ∼1% and microglia = ∼31%) (Fig. 2). Interestingly, switching from more aggressive histological phenotypes (i.e., GBM) to less aggressive ones (i.e., oligodendroglioma) the relative percentage of macrophages decreases while the percentage of microglia cells increases. These data are in agreement with the hypothesis that gliomas in the early stages of their development primarily contain brain-resident microglia cells, whereas macrophage phenotype is associated with higher grades [23]. Patients with astrocytoma and GBM also showed a high fraction of vascular cells (∼44% and ∼14%, respectively), probably due to increased microvascular proliferation of these high-grade tumors compared with oligodendrogliomas. Regarding the lymphoid populations, T cells represent the most abundant fraction, with a greater number of CD8 cells observed in GBM and oligo-astrocytoma.

We also evaluated whether there is a significant association between the different cell types composing the microenvironment and the molecular glioma subtypes [42]. We correlated the percentage of cells classified in 1 of the glioma subtypes with the percentages of non-malignant cell types only for patients in which the cells were not selected with any gate strategy (Fig. S3). This analysis showed a significant correlation between the mesenchymal subtype and the presence of macrophages (ρ = 0.47, *P* = 0.015), myeloid-derived suppressor cells (MDSCs) (ρ = 0.56, *P* = 0.003), dendritic cells (ρ = 0.40, *P* = 0.039), and astrocytes (ρ = 0.42, P = 0.033); proneural subtype was significantly associated with the presence of oligodendrocytes (ρ = 0.43, *P* = 0.028); and classical subtype was significantly associated with the presence of microglia (ρ = 0.45, *P* = 0.019).

### Cross-talk between mesenchymal GBM tumor cells and myeloid cells in the glioma microenvironment

Because cells of myeloid lineage account for ∼50–60% of non-neoplastic cells, we first focused on interactions occurring between tumor and myeloid cells, including bone marrow–derived macrophages and microglia. The analysis was performed on the patients with a sufficient number of detected myeloid cells (n = 39 patients). Each patient was tested to identify both paracrine and autocrine interaction pairs. Only significant L–R pairs were kept, with *P* < 0.05 and the constraint a total scTHI score >0.50 in both clusters. Altogether, we detected 368 significant L–R pairs across datasets by filtering out all interactions occurring in <4 patients. Approximately 80% of detected interactions (298 of 368) showed considerable autocrine signaling (Table S4). The remaining 20% of the identified interactions (n = 70) showed paracrine signaling, where the interaction genes were preferably expressed on only 1 of the 2 clusters (Table S4). Several high-scored interactions occurred specifically in few patients owing to the typical heterogeneity of the tumor being considered. Interestingly, a high fraction of detected pairs (n = 56, ∼15%) was shared between ≥50% of patients, suggesting the presence of common tumor–host signaling mechanisms in glioma. Many of the inferred interactions involved genes of the chemokine and cytokine family (e.g., CCL5, CCR1, CCRL2), Toll-like receptors (e.g., TLR2, TLR4), transforming growth factor genes, TNF genes, MHC proteins (e.g., HLA-E, CD74), growth factors and their receptors (e.g., EGFR, PDGFB, PDGFC, PDGFRA, IGF1, IGF1R), cell adhesion molecules (e.g., integrins), enzymes with inhibitor activity, and metalloproteinases. Gene ontology enrichment analysis revealed that secreted ligands or activated receptors in tumor cells are typically involved in processes of extracellular matrix remodeling, cell chemotaxis, Notch signaling, axonogenesis, and gliogenesis (Fig. S4A and Table S5). In contrast, receptors and ligands detected on myeloid cells modulate biological processes such as leukocyte chemotaxis and migration, cell–cell adhesion, cytokine production, myeloid cell differentiation, reactive oxygen species metabolic processes, and regulation of vasculature development (Fig. S4B and Table S5).

**Figure 3:**
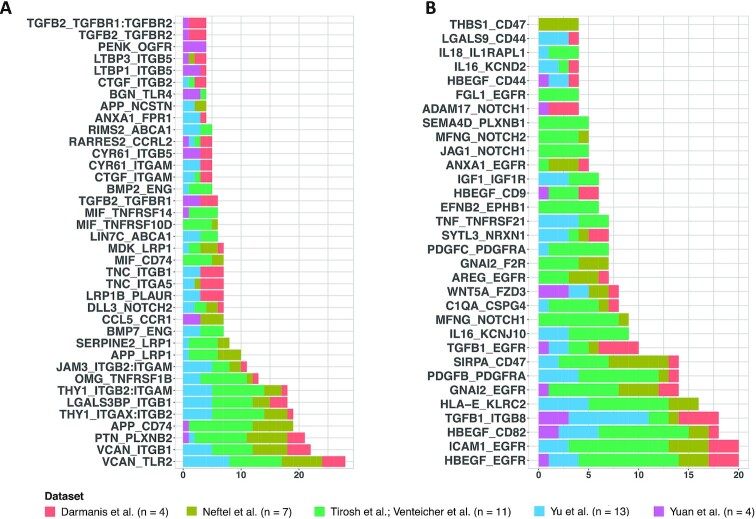
Paracrine tumor–myeloid cell interactions. Bar plots show significant paracrine L–R interactions (*P* ≤0.05 and scTHI score ≥0.50) occurring between tumor and myeloid cells shared in ≥4 patients. On the x axis are shown the number of patients in which each interaction occurred. (A) Interaction pairs in which the ligand is expressed on tumor cells and the receptor on myeloid cells. (B) Interaction pairs in which the ligand is expressed on myeloid cells and the receptor on tumor cells. Datasets are from [17, 22–26].

**Figure 4:**
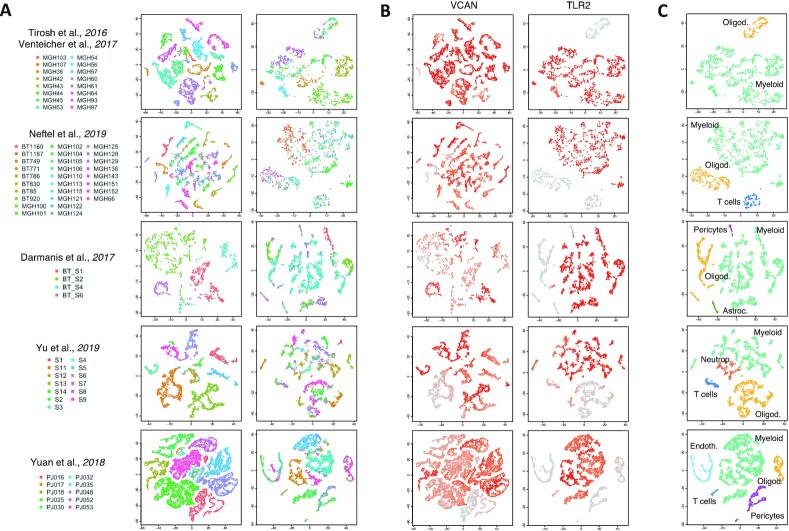
VCAN-TLR2 interaction. t-distributed stochastic neighbor embedding (t-SNE) plots of tumor and non-tumor cells for each dataset analyzed. (A) Each cell is colored by patient (right, non-tumor, and left, tumor cells). (B) Each cell is colored according to expression value of the genes *VCAN* and *TLR2* in tumor and non-tumor cells, respectively. (C) t-SNE plot of non-tumor cells colored according to cell type classification. Datasets are from [17, 22–26].

The significant interactions identified running scTHI in paracrine mode, and most common among all patients with glioma, were VCAN-TLR2 (72% of patients, *P* = 4.40 · 10^−67^) and HBEGF-EGFR (51% of patients, *P* = 6.97 · 10^−44^) (Fig. 3 and Fig. S5). The Versican gene (*VCAN*) codes for an extracellular matrix proteoglycan, typically involved in processes of cell adhesion, proliferation, and migration. It is highly expressed in glioma cells, where it strongly contributes to tumor progression mechanisms [43]. According to these observations, we found *VCAN* diffusely expressed in the tumor cells of all the datasets considered (Fig. 4). The interaction's partner, the Toll-like receptor 2 (TLR2), is a membrane protein expressed on the surface of many cell types, including monocytes and macrophages, and it is involved in pattern recognition signaling pathway and innate immunity activation. *TLR2* is highly and specifically expressed only in cells of the microenvironment of myeloid origin, and low expressed in other cell types (Fig. 4B). The interaction occurring among VCAN and TLR2 represents an effective link between inflammation and tumor progression. Indeed, the Versican protein, released by tumor cells in the extracellular space, binds TLR2, activating multiple cell types in the tumor microenvironment, including myeloid, fibroblasts, and endothelial cells, and promotes the production of many proinflammatory cytokines [44, 45]. The activation of the TLR2 downstream signaling pathway also induces the expression of metalloproteinases involved in extracellular matrix degradation to promote tumor expansion [43]. The most common interaction with the receptor on the tumor cells was composed by the pair EGFR and HBEGF, which could tend to promote tumor growth. In fact, the epidermal growth factor receptor (EGFR) is a tumor-promoting receptor commonly amplified in gliomas, and the EGF-like growth factor (HBEGF) is a protein highly expressed in regulatory macrophages with immunosuppressive activity [46]. Among other significant interactions of interest identified by scTHI, 1 involves the macrophage migration inhibitory factor (MIF) and its receptor CD74 (18% of patients); it plays a role in tumorigenesis, exerting pro-tumorigenic effects such as enhancing proliferation, tumor vascularization, and inhibition of apoptosis [47].

We also found that multiple significant interaction pairs involved ligands (TGFB1, TGFB2), receptors (TGFBR1, TGFBR2), and regulators (LTBP1, LTBP3) of the TGFβ signaling pathway. The TGFβ pathway is a known driver of immunosuppression in multiple epithelial tumors and in GBM, in which it also drives other hallmarks of aggressiveness (e.g., cancer stem cells, migration, and invasion) leading to poor survival [48, 49].

The identification of interaction pairs in autocrine mode (Table S4) revealed that this signaling is much more conserved among patients than paracrine signaling. As expected, some of the paracrine interactions described above were also identified as autocrine, although they have a preferential paracrine directionality. Among the top-scored L–R autoc-rine pairs, there was an interaction between RPS19 and C5AR1 (Fig. S6), detected in all the patients with glioma tested (n = 39, *P* = 6.68 · 10^−76^). Although this interaction has never been described in the context of glioma, the ribosomal protein S19 (*RPS19*) is upregulated in breast and ovarian cancers, and its interaction with the C5a receptor 1 (C5AR1), expressed on tumor-infiltrating myeloid cells, has an immunosuppressive effect. RPS19 induces the production of anti-inflammatory cytokines, the activation of T helper 2 (T_H_2) and regulatory T cells, and the reduction of infiltrating CD8^+^ T cells into tumors. It was also noted that reducing RPS19 in tumor cells or blocking the C5AR1-RPS19 interaction decreases RPS19-mediated immunosuppression, impairing the tumor growth [50]. These observations could be translated into gliomas, representing another potential therapeutic target.

### Cross-talk between proneural GBM tumor cells and oligodendrocytes in the glioma microenvironment

Whereas the microenvironment of mesenchymal GBM was massively enriched by myeloid cells, proneural GBM contained a low number of myeloid cells but exhibited significant infiltration by oligodendrocytes, which alone accounted for ∼17% of tumor-infiltrating cells. Altogether we tested 30 tumor samples, among the 71, for which we identified an adequate number of oligodendrocytes in their microenvironment. Each individual tumor was tested to identify both paracrine and autocrine interaction pairs, and only significant interactions shared in ≥4 patients were considered. Overall, we found 26 paracrine and 126 autocrine interactions (Table S6). Gene ontology enrichment analysis revealed that the cross-talk between tumor cells and oligodendrocytes mainly involved signaling pathways related to cell growth and nervous system development, such as axonogenesis, regulation of neurogenesis, extracellular matrix organization, developmental cell growth, gliogenesis, and synapse organization (Table S7). Indeed, among the top-scored paracrine interactions (Fig. S7) there were several ligands and receptors involved in cell-cell adhesion, angiogenesis, and tumorigenesis, such as MDK–LRP2 (12 of 30 patients, *P* = 1.86 · 10^−31^) and JAM2-JAM3 (11 of 30 patients, *P* = 1.96 · 10^−28^).

The MDK–LRP2, which scored as top interaction among GBM tumor cells (ligand) and oligodendrocyte (receptor), is especially intriguing because overexpression of MDK (midkine) has been shown in several human tumors and recently was reported as a driver of aberrant proliferation, poor survival, and pharmacological resistance in human glioma [51]. In many patients, we also detected as significant the interaction occurring between the platelet-derived growth factor subunit A (PDGFA) and its receptor, PDGFRA (11 of 30 patients, *P* = 3.97 · 10^−25^). PDGFA is a classical marker of oligodendrocytes required for normal oligodendrocyte development. However, the overexpression of its receptor is a hallmark of proneural GBM, where it plays a critical role during tumor development and progression [52]. We found that ∼72% of cells expressing *PDGFRA* at the top 20% of the ranked expression profile were classified as proneural subtype (Fisher exact test *P* < 2.2 · 10^−16^ and odds ratio = 5.4). Other L–R pairs were specifically related to the development of the nervous system and promoting neuronal adhesion, i.e., RGMB-NEO1 (11 of 30 patients, *P* = 1.96 · 10^−28^) and SEMA5A-PLXNB3 (8 of 30 patients, *P* = 9.72 · 10^−20^) bindings. The most shared interaction detected was CNTN2-NRCAM (15 of 30 patients, *P* = 2.35 · 10^−36^). The contactin 2 (*CNTN2*) gene, also known as axonal glycoprotein TAG-1 (*TAX-1*), codes for a cell adhesion molecule that plays an important role in axonal elongation, axonal guidance, and neuronal cell migration [53]. The gene is also amplified and aberrantly expressed in GBM, where it is involved in neoplastic glial cell migration and tumorigenesis [54]. TAX-1 binds numerous molecules, among which the neuronal cell adhesion molecule (NRCAM) gene. In response to contactin binding, NRCAM promotes directional axonal cone growth in fetal nervous system development and mediates neurite outgrowth in the peripheral nervous system [55]. Although less studied, *NRCAM* is also overexpressed in high-grade astrocytoma and GBM, representing a marker for brain tumor detection and a putative therapeutic target [56].

### Cross-talk involving T cells in glioma

The scTHI classification of non-tumor cells identified subpopulations of CD8^+^ T cells infiltrating the microenvironment of a small subset of patients (n = 8). Although T cells play a fundamental role in antitumor immunity, GBM is particularly adept at sabotaging immune surveillance, causing severe T-cell dysfunction, both qualitative and quantitative [57]. To better understand the complex role of T cells in the glioma microenvironment, we simultaneously evaluated the cross-talk existing between CD8^+^ T cells and tumor and myeloid cells, respectively.

We first investigated the putative L–R interactions occurring between tumor and CD8^+^ T cells. Altogether, we detected 16 paracrine and 120 autocrine significant L–R pairs (Table S8), considering only interactions occurring in >3 patients. Most of the identified interactions involved genes of the major histocompatibility complex Class I, chemokines, interleukins, interferon-γ, and TNF signaling genes. Among all interactions detected in paracrine mode (Fig. S8A–D), CADM1-CRTAM was the most common and high-scored L–R pair (6 of 8 patients, *P* = 1.90 · 10^−12^, Fig. S8E–G). The *CADM1* gene, also known as *TSLC1*, was originally identified as a non–small-cell lung cancer tumor suppressor. The gene encodes a cell surface protein, called NECL-2, which mediates epithelial cell junctions. We found that the CRTAM receptor is highly expressed in CD8 cells with respect to other non-tumor cells. Although recent studies have shown a CADM1 loss at the protein and messenger RNA levels in high-grade (World Health Organization III/IV) glioma compared with low-grade glioma [58], the fact that we found this interaction mainly in patients with GBM in association with infiltrating CD8^+^ T cells further supports the tumor suppressor role of this gene.

Similarly, we analyzed the interactions between CD8^+^ T cells and myeloid cells, identifying 53 paracrine (Fig. S9 and Table S9) and 159 autocrine L–R pairs. The detected interactions mainly involved (i) chemoattractant chemokine ligands and receptors, such as CXCL16/CXCR6, CXCL12/CXCR3, CLL8/CCR2, CCL5/CCR1, and others; (ii) immune checkpoint genes, such as *CD86, CD28, CTLA*-4, and *LGALS9*; and (iii) lymphotoxins α and β (LTA and LTB) and other TNF family members, which could modulate T cells' immunity through different signaling pathways. The chemokine receptor CXCR6, typically expressed on different T-cell compartments, and its ligand CXCL16, secreted by macrophage cells, were found highly expressed in all patients. They induce proliferation and migration of tumor-associated leukocytes, affecting cancer cell growth with pro-tumorigenic inflammation [59]. The CXCR3 receptor is also highly expressed on T cells and plays an important role in T-cell trafficking and function [60]. LTA and LTB are cytokines produced by lymphocytes belonging to the TNF family. Although they were originally identified as lymphocyte products capable of exerting cytotoxic effects on tumor cells *in vitro* and*invivo*, recent studies have shown that lymphotoxin (LT) contributes to several effector responses of both the innate and adaptive immune systems. Binding to both TNFRSF1A/TNFRSF1B and LTβR with high affinity, LT-mediated signaling is essential for the development of secondary lymphoid tissues [61]. Moreover, the activation of LTβR on macrophages by T-cell–derived LTs controls proinflammatory response, inducing cross-tolerance to TLR4 and TLR9 ligands through the downregulation of proinflammatory cytokines and a negative regulation of nuclear factor κB activation induced by TLR signaling [62]. Finally, we also identified 2 opposite immune checkpoint signals in almost all patients involving CD28 (n = 8, *P* = 4.06 · 10^−18^) and CTLA-4 (n = 6, *P* = 2.50 · 10^−12^) to binding CD86. Usually, CD86/CD28 binding results in activation and initiation of T-cell effector function. However, high levels of *CTLA*-4 expression on T cells, probably induced by cancer, create competition with CD28 and result in insufficient co-stimulation and a loss of T-cell proliferation and function [57].

## Discussion

In this work we describe scTHI, a novel computational approach to identify active L–R interactions in single cells, and apply it to 5 glioma datasets encompassing 71 patients, 45,550 malignant cells, and 11,510 non-malignant cells. We presented a comprehensive map of active tumor–host interactions in glioma (Fig. S10). We have first shown that scTHI can identify recently discovered interactions validated by CODEX [15] and then explored common L–R cross-talk in glioma. Our results confirm, using a much larger scale, that myeloid cells make up the bulk of the microenvironment in glioma and that the ratio between macrophages and microglia cell increases with more aggressive phenotypes as has been previously observed for IDH-mutant glioma [23]. Interestingly, the use of a large patient cohort allowed us to link the specific immune compartments with glioma subtypes. Indeed, we report that the presence of macrophages and myeloid-derived suppressor cells was significantly correlated with the mesenchymal subtype, as also described in [63], whereas the proneural subtype was significantly correlated with the presence of oligodendrocytes; cells in the classical subtype, on the other hand, tend to be correlated with the presence of microglia.

Our complete map of cross-talk between tumor and myeloid cells allowed us to identify some known and some novel potential targets for promoting anticancer therapy enhancing the immune response. Although a significant number of interactions are specific to few patients, when collectively analyzed, our findings show that the members of the interactions on the malignant cells enrich common pathways from those on immune cells. The ligand and receptor expressed on cancer cells participate in pathways such as extracellular matrix remodeling, cell chemotaxis, Notch signaling, axonogenesis, and gliogenesis. Instead, receptors and ligands detected on myeloid cells modulate biological processes such as leukocyte chemotaxis and migration, cell-cell adhesion, cytokine production, myeloid cell differentiation, reactive oxygen species metabolic processes, and regulation of vasculature development. This pattern underlines a highly connected tumor–host communication network in glioma, where many ligands and receptors can interact on the same cell type.

We have also identified a subset of interactions that are highly conserved across different patients and datasets in paracrine mode, showing that *TLR2* is specifically and exclusively upregulated in glioma-associated microglia; in contrast, astrocytes and glioma cells expressed only low levels of *TLR2*. It is known that versican is a glioma-derived endogenous TLR2 mediator that regulates microglial MT1-MMP expression for tumor expansion [45]. Microglial upregulation can be abolished by targeting TLR2, with potential therapeutic benefits in glioma progression [43]. Our results confirm that TLR2 is a candidate for adjuvant therapy in the treatment of glioma [64]. We also described the interaction between EGFR and HBEGF: the feedback loop between these 2 genes regulates astrocytes' maturation [65] and promotes gliomagenesis in specific contexts; the silencing of 1 of the partners tends to reduce tumor growth and increases survival *in vivo* [66]. Another common interaction across several patients includes the pair MIF-CD74. Although MIF is a proinflammatory cytokine, it also exerts immunosuppressive functions, influencing the M1/M2 polarization of tumor-associated macrophages [67]. In fact, recent studies have shown that the MIF-CD74 binding activates a signaling pathway, resulting in M2 shift in microglial cells, macrophages, and dendritic cells [68]. The inhibition of MIF signaling on these cells restores the antitumor immune response, leading to a decrease in the expression of immunosuppressive factors and a reacquired capacity in cytotoxic T-cell activation [69]. In addition, the CD74 receptor after activation is quickly internalized and recycles; therefore, it constitutes an attractive target for anticancer antibody-based treatment strategies. We reported the L–R interaction including THY1 (CD90) and ITGAM/ITGB2 (MAC-1, CD11B/CD18), involved in leukocyte recruitment in response to inflammatory signals (46% of patients). CD90 is a specific surface marker highly expressed in glioma-associated mesenchymal stem cells [70] and drives glioma progression through SRC-dependent mechanisms increasing proliferation, migration, and adhesion [71]. On the other hand, the CD11B/CD18 integrins complex is abundantly expressed on the monocyte/macrophages surface, where it is involved in critical adhesive reactions including the recruitment of myeloid cells to the tumor site [72]. Moreover, CD11B is a negative regulator of immune suppression, representing an interesting target for cancer immunotherapy [73]. In fact, CD11B activation promotes pro-inflammatory macrophage polarization, while its inhibition leads to immune-suppressive macrophage polarization, vascular maturation, and accelerated tumor growth.

When we applied our algorithm in autocrine mode, we found some common interactions shared by all patients of our cohort. RPS19, for example, is an abundant intracellular protein that is expressed by virtually all cells in the body, and its extracellular functions, including interaction with C5AR1, are activated upon its release from dying cells [74]. The importance of C5AR1–RPS19 interaction for immunosuppression was recently demonstrated, showing that the downregulation of *RPS19* in tumor cells or pharmacological blockade of C5AR1 by C5ARA reduced this immunosuppression and led to the generation of tumor-specific T-cell responses and slower tumor growth in breast and ovarian cancer cells [50]. We have shown that the C5AR1–RPS19 interaction is among the most common signaling cross-talk between tumor cells and their microenvironment across multiple patients, making these molecules an interesting target for therapeutic strategies.

Our analysis confirmed that proneural GBMs are significantly infiltrated by oligodendrocytes. MDK resulted in 1 of the top possible mediators of the interaction between glioma cells and oligodendrocytes through its ligand LRP2. The oncogenic role of MDK, promoting proliferation and pharmacological resistance in glioma, may involve the release of Sonic Hedgehog (SHH) from LRP2 sequestration in oligodendrocytes [75], thus functioning to activate one of the best-known activators of proliferation and stemness of brain tumors [76]. The single-cell characterization of proneural glioma evidenced that the concurrent expression of high levels of the PDGFA ligand by the abundant oligodendrocytes infiltrating PDGFRA-amplified proneural GBM provide the crucial initiating signaling event for the effects that this pathway has for proliferation, stemness, and progression of brain tumors [77].

Recent trials have shown that endogenous T cells play a significant role in the prolonged survival time of patients with glioma [2, 78]. However, GBM-induced immune suppression is a major obstacle to an effective and durable immune-mediated antitumor response. We have shown that a significant number of patients with glioma present subpopulations of CD8^+^ T cells. The presence and T-cell clonal diversity in the tumor microenvironment has also been associated with response to immune therapy in glioma [79]. Our analysis reported that this mechanism of tumor suppression could be mediated by CADM1 and its receptor CRTAM. Typically, CADM1 performs its antitumor activity ensuring that cells grow in organized layers, inhibiting uncontrolled growth. Moreover, NECL-2 binds NK and CD8^+^ T cells through a receptor known as class I-restricted T-cell–associated molecule (CRTAM), which is expressed only on activated cells [58]. The interaction among CRTAM and NECL-2 promotes cytotoxicity of NK cells and interferon γ (IFN-γ) secretion of CD8^+^ T cells *in vitro* [80].

## Methods

### Immune cell type classification

To evaluate the enrichment of each immune cell type in glioma samples we used the collection of 295 signatures and the Normalized Enrichment Score (NES) of the Mann-Whitney-Wilcoxon gene set test (mww-GST) that we previously described [39]. Briefly, NES is an estimate of the probability that the expression of a gene in the gene set is greater than the expression of a gene outside this set:

\begin{equation*} \mathrm{NES} = 1 - \frac{U}{{mn}}, \end{equation*}

where *m* is the number of genes in a gene set, *n* is the number of those outside the gene set, $U = mn + m( {m + 1} )\ - T$, and *T* is the sum of the ranks of the genes in the gene set. We applied single-cell mww-GST and classified each cell according to the signature with the highest NES and corrected *P* < 0.01.

### scTHI scores

The scTHI scores are computed between pairs of clusters of single cells, assigning a score to each interaction of the table. A typical example is when we have 1 cluster from the microenvironment (e.g., macrophages) and 1 cluster from the tumor cells. Given a single-cell profile *G*, ranked from the high-expressed to the low-expressed genes, we call *G^T^* the set of genes in the top of the ranked profile (in all the reported experiments we use the top 20%; the accompanying code allows the threshold to be selected). Let the 2 clusters also be called *A* and *B*, then for every L–R pair of the interaction table we compute the following score:

\begin{equation*} s(L,R) = \frac{1}{2}\left[ {\frac{1}{{\left| A \right|}}\sum\nolimits_{G \in A} {{I_{{G^T}}}} (L) \cdot {I_{\overline {{G^T}} }}(R) + \frac{1}{{\left| B \right|}}\sum\nolimits_{G \in B} {{I_{{G^T}}}} (R) \cdot {I_{\overline {{G^T}} }}(L)} \right], \end{equation*}

where *I* is the indicator function

\begin{equation*} {I_X}(y) = \left\{ \begin{array}{@{}l@{}} 1\ \ {\mathrm{if}}\ \ y \in X\\ 0\ \ {\mathrm{otherwise}} \end{array} \right. \end{equation*}

Briefly, the score *s* is the average between 2 percentages: the percentage of cells in cluster *A* where the gene *L* is at the top of the ranked list and gene *R* is not at the top and vice versa for cluster *B* in the second percentage; it tends to give a higher score to paracrine interactions (in order to also score autocrine interactions the second term in the product of the 2 summations in the equation can be removed). A null distribution of the interaction score to assign significance is then obtained by a bootstrap procedure, shuffling the input data.

### Significance of recurrent interactions among patients

We use the binomial test to quantify the significance of the observed recurrent interaction among patients. To estimate the *p* parameter of the binomial distribution under the null hypothesis, for each cell compartment (myeloid, oligodendrocyte, T lymphocyte), we first generated a synthetic patient with 1,000 cells, 500 from the clusters of malignant cells and 500 from the cluster of non-malignant cells, and then generated 100 random L–R tables by shuffling the rows of the original interaction table. This generates a set of random L–R pairs preserving the overall distribution of the expression of the genes involved in the interaction. By applying scTHI to the synthetic patient and the random interaction tables, we compute the expected number of active interactions per patient in the null case. We then use this value as the *p* parameter of the binomial test. It is worth mentioning that we obtain exactly the same estimate (up to the fourth decimal digit) if, on the contrary, we generate a synthetic interaction table and sample on the patients' profiles. For each interaction described in the text we report its significance in Tables S4, S6, S8, and S9.

### Gene Ontology enrichment analysis

The GO category enrichments of ligand and receptor genes detected by scTHI analysis were performed using clusterProfiler [81]. Enriched GO terms were filtered using an adjusted *P*-value cut-off of 0.0001.

## Availability of Source Code and Requirements

Project name: single-cell Tumor Host Interaction

Project home page: https://bioconductor.org/packages/devel/bioc/html/scTHI.html

Operating system: Platform independent

Programming language: R

License: GPL-2

RRID:SCR_018918

BiotoolsID: scTHI

## Availability of Supporting Data and Materials

Imputed matrices for malignant and non-malignant cells are available in the *GigaScience* GigaDB database [82].

## Additional Files

**Figure S1**. Examples of L–R pairs: mean expression vs ranks. (A) t-SNE plot of tumor and myeloid cells in patient PJ017 colored by phenotype (left). t-SNE plots of 3 different interaction pairs (APP_FPR2, BGN_TLR4, MMP7_ERBB4) colored according to the expression value of each gene in patient PJ017 (right). (B) t-SNE plot of tumor and myeloid cells in patient MGH42 colored by phenotype (left). t-SNE plots of 2 different complex interaction pairs (THY1_ITGAX:ITGB2 and ITGB1:ITGA9_SPP1) colored according to the expression value of each gene in patient MGH42 (right). (C) t-SNE plot of interaction pairs occurring among tumor and myeloid cells in patient MGH42. The interaction between *IL13* and *IL13RA1* genes was detected in paracrine mode (left); the interaction between *CALR* and *LRP1* genes was detected in autocrine mode (right). Cells are colored according to the expression value of each gene.

**Figure S2**. scTHI recovers validated interactions. The bar plots show the top 15 significant interactions identified between (A) red-pulp and monocyte-derived macrophage cells, (B) red-pulp macrophages and neutrophils, and (C) monocyte-derived macrophages and neutrophils. The interactions spatially resolved in [15] are pointed out with an arrow.

**Figure S3**. Association between immune cell types and glioma subtypes. Heat map of correlation between the percentage of cells classified in one of the glioma subtypes with the percentages of non-tumor cell types. *P ≤ 0.05, **P ≤ 0.01.

**Figure S4**. Enriched GO categories of L–R pair. Network of the most represented biological process category enriched by L–R partners on tumor (A) and on myeloid cells (B).

**Figure S5**. Specific and shared paracrine tumor–myeloid cell interactions among datasets. Dot plots showing the significant paracrine L–R interactions occurring between tumor and myeloid cells per dataset. (A) Interaction pairs in which the ligand is expressed on tumor cells and the receptor on myeloid cells. (B) Interaction pairs in which the ligand is expressed on myeloid cells and the receptor on tumor cells.

**Figure S6**. RPS19–C5AR1 interaction. t-SNE plots of tumor and non-tumor cells for each dataset analyzed. (A) Each cell is colored by patient (right, non-tumor and left, tumor cells). (B) Each cell is colored according to expression value of the genes *RPS19* and *C5AR1* in tumor and non-tumor cells, respectively. (C) t-SNE plot of non-tumor cells colored according to cell type classification.

**Figure S7**. Paracrine tumor–oligodendrocyte cell interactions. Bar plots show significant paracrine L–R interactions (*P* ≤ 0.05 and scTHI score ≥ 0.50) occurring between tumor and oligodendrocyte cells shared in ≥4 patients. On the x axis are shown the number of patients in which each interaction occurred. (A) Interaction pairs in which the ligand is expressed on tumor cells and the receptor on oligodendrocyte cells. (B) Interaction pairs in which the ligand is expressed on oligodendrocyte cells and the receptor on tumor cells. (C) Interaction pairs as in (A) for each dataset. (D) Interaction pairs as in (B) for each dataset.

**Figure S8**. Paracrine tumor–CD8 cell interactions. Bar plots show significant paracrine L–R interactions (*P*≤ 0.05 and scTHI score ≥ 0.50) occurring between tumor and CD8 cells shared in ≥3 patients. On the x axis the number of patients in which each interaction occurred is shown. (A) Interaction pairs in which the ligand is expressed on tumor cells and the receptor on CD8 cells. (B) Interaction pairs in which the ligand is expressed on CD8 cells and the receptor on tumor cells. (C) Interaction pairs as in (A) for each dataset. (D) Interaction pairs as in (B) for each dataset. (E) CADM1-CRTAM interaction: each cell is colored by patient (right, non-tumor and left, tumor cells). (F) As in (E) where each cell is colored according to expression value of the genes *CADM1* and *CRTAM* in tumor and non-tumor cells, respectively. (F) t-SNE plot of non-tumor cells colored according to cell type classification.

**Figure S9**. Paracrine myeloid–CD8 cell interactions. Bar plots show significant paracrine L–R interactions (*P* ≤ 0.05 and scTHI score ≥ 0.50) occurring between myeloid and CD8 cells shared in ≥3 patients. On the x axis the number of patients in which each interaction occurred is shown. (A) Interaction pairs in which the ligand is expressed on myeloid cells and the receptor on CD8 cells. (B) Interaction pairs in which the ligand is expressed on CD8 cells and the receptor on myeloid cells. (C) Interaction pairs as in (A) for each dataset. (D) Interaction pairs as in (B) for each dataset.

**Figure S10**. A map of tumor–host interactions in glioma. Chord diagram of paracrine tumor–host interaction detected by scTHI. The color of the arcs indicates the clusters of cells among which the interaction has been identified. The origin of the arc indicates that the ligand or receptor is expressed on the tumor, while the arrow of the arc indicates that the ligand or receptor is expressed on the cells of the microenvironment.

**Table S1**. Ligand–receptor interactions. List of ligand–receptor interaction pairs provided in scTHI.

**Table S2**. Signatures. List of immune system and tissue cell type signatures provided in scTHI.

**Table S3**. Non-tumor cell classification. Phenotype classification of tumor microenvironment cells in glioma datasets performed by TME.classification function provided by scTHI. The second sheet reports the breakdown for each dataset.

**Table S4**. Tumor–myeloid cell interactions. List of significant paracrine and autocrine L–R interactions occurring between tumor and myeloid cells from considered datasets detected by scTHI.

**Table S5**. Enriched GO categories of L–R partners in tumor and myeloid cells. List of significant enriched GO biological processes of ligand and receptor genes expressed in tumor and myeloid cells, respectively.

**Table S6**. Tumor–oligodendrocyte cell interactions. List of significant paracrine and autocrine L–R interactions occurring between tumor and oligodendrocyte cells from considered datasets detected by scTHI.

**Table S7**. Enriched GO categories of L–R partners in tumor and oligodendrocyte cells. List of significant enriched GO biological processes of ligand and receptor genes expressed in tumor and oligodendrocyte cells, respectively.

**Table S8**. Tumor–CD8 cell interactions. List of significant paracrine and autocrine L–R interactions occurring between tumor and CD8 T cells detected by scTHI.

**Table S9**. Myeloid–CD8 cell interactions. List of significant paracrine and autocrine L–R interactions occurring between myeloid and CD8 T cells detected by scTHI.

giaa109_GIGA-D-20-00015_Original_Submission

giaa109_GIGA-D-20-00015_Revision_1

giaa109_Response_to_Reviewer_Comments_Original_Submission

giaa109_Reviewer_1_Report_Original_Submission Valentine Svensson -- 5/4/2020 Reviewed

giaa109_Reviewer_1_Report_Revision_1 Valentine Svensson -- 8/29/2020 Reviewed

giaa109_Reviewer_2_Report_Original_Submission Jiansong Ji -- 5/8/2020 Reviewed

giaa109_Reviewer_3_Report_Original_Submission Geng Chen -- 6/3/2020 Reviewed

giaa109_Reviewer_3_Report_Revision_1 Geng Chen -- 8/17/2020 Reviewed

giaa109_Supplemental_Figures_and_Tables

## Abbreviations

CITE-seq: cellular indexing of transcriptomes and epitopes by sequencing; CNV: copy number variation; CODEX: co-detection by indexing; CTLA-4: cytotoxic T lymphocyte–associated protein 4; ENCODE: Encyclopedia of DNA Elements; GBM: glioblastoma; GEO: Gene Expression Omnibus; GO: Gene Ontology; ICI: immune checkpoint inhibitor; IDH: isocitrate dehydrogenase; L–R: ligand–receptor; LT: lymphotoxin; MDSC: myeloid-derived suppressor cell; MIF: migration inhibitory factor; mww-GST: Mann-Whitney-Wilcoxon gene set test; NES: Normalized Enrichment Score; NK: natural killer; PD1: programmed cell death protein 1; PD-L1: programmed death–ligand 1; scRNA: single-cell RNA; scTHI: single-cell Tumor–Host Interaction; CITE-seq: cellular indexing of transcriptomes and epitopes by sequencing; TGF: transforming growth factor; TNF: tumor necrosis factor.

## Competing Interests

The authors declare that they have no competing interests.

## Funding

The research leading to these results has received funding from Associazione Italiana per la Ricerca sul Cancro (AIRC) under IG 2018–ID. 21846 project and from Italian Ministry of Research Grant PRIN 2017XJ38A4_004.

## Authors’ Contributions

Conceptualization: F.P.C., A.I., M.C.; Methodology: F.P.C., J.Z., L.G., S.M.P., A.I., M.C.; Software: F.P.C., L.C., F.D.A.; Resources: K.Y., F.T., J.Y., P.A.S., M.S., X.D.S., A.L.; Data Curation: K.Y., F.T., J.Y., P.A.S., M.S., X.D.S., A.L.; Writing—Review and Editing: F.P.C., M.S.,D.B., A.I., M.C.; Supervision: A.I., M.C.; Funding Acquisition: M.C.
