# Supplementary material for: A map of tumor–host interactions in glioma at single-cell resolution
Source: Gigascience. 2020 Oct 14;9(10):giaa109. doi: 10.1093/gigascience/giaa109 (PMC7645027; doi:10.1093/gigascience/giaa109)
Supplement: giaa109_GIGA-D-20-00015_Original_Submission [file giaa109_giga-d-20-00015_original_submission.pdf]

GigaScience

A MAP of tumor-host interactions in glioma at single cell resolution

--Manuscript Draft--

**Manuscript Number:**	GIGA-D-20-00015
**Full Title:**	A MAP of tumor-host interactions in glioma at single cell resolution
**Article Type:**	Research
**Funding Information:**	Associazione Italiana per la Ricerca sul Cancro (IT) (IG 2018 - ID. 21846) — prof. Michele Ceccarelli
**Abstract:**	Background<br>Single-cell RNA sequencing is the reference technique to characterize the heterogeneity of tumor microenvironment. The composition of the various cell types making up the microenvironment can significantly affect the way in which the immune system activates cancer rejection mechanisms. Understanding the cross-talk signals between immune cells and cancer cells is a fundamental for the identification immuno-oncology therapeutic targets .<br>Results<br>We present a novel method, single cell Tumor-Host Interaction tool (scTHI), to identify significantly activated ligand-receptor interactions across clusters of cells from single-cell RNA sequencing data. We apply our approach to uncover the ligand-receptor interactions in glioma using six publicly available human glioma datasets encompassing 57,060 gene expression profiles from 71 patients. By leveraging this large-scale collection we show that unexpected cross-talk partners are highly conserved across different datasets in the majority of the tumor samples. This suggests that shared cross-talk mechanisms exist in glioma.<br>Conclusions<br>Our results provide a complete map of the active tumor-host interaction pairs in glioma that can be therapeutically exploited to reduce the immunosuppressive action of the microenvironment in brain tumor.
**Corresponding Author:**	Michele Ceccarelli<br>Universita degli Studi di Napoli Federico II<br>Naples, NA ITALY
**Corresponding Author Secondary Information:**	
**Corresponding Author's Institution:**	Universita degli Studi di Napoli Federico II
**Corresponding Author's Secondary Institution:**	
**First Author:**	Michele Ceccarelli
**First Author Secondary Information:**	
**Order of Authors:**	Michele Ceccarelli
	Francesca Pia Caruso, PhD
	Luciano Garofano, PhD
	Fulvio D'Angelo
	Kai Yu, PhD
	Fuchou Tang, PhD
	Jinzhou Yuan
	Jing Zhang
	Luigi Cerulo
	Davide Bedognetti
	Peter Sims
	Mario Suvà

Powered by Editorial Manager® and ProduXion Manager® from Aries Systems Corporation

	Xiao Dong Su
	Anna Lasorella
	Antonio Iavarone
**Order of Authors Secondary Information:**	
**Additional Information:**	
**Question**	**Response**
Are you submitting this manuscript to a special series or article collection?	No
**Experimental design and statistics**<br><br>Full details of the experimental design and statistical methods used should be given in the Methods section, as detailed in our Minimum Standards Reporting Checklist. Information essential to interpreting the data presented should be made available in the figure legends.<br><br>Have you included all the information requested in your manuscript?	Yes
**Resources**<br><br>A description of all resources used, including antibodies, cell lines, animals and software tools, with enough information to allow them to be uniquely identified, should be included in the Methods section. Authors are strongly encouraged to cite Research Resource Identifiers (RRIDs) for antibodies, model organisms and tools, where possible.<br><br>Have you included the information requested as detailed in our Minimum Standards Reporting Checklist?	Yes
**Availability of data and materials**<br><br>All datasets and code on which the conclusions of the paper rely must be either included in your submission or deposited in publicly available repositories (where available and ethically appropriate), referencing such data using a unique identifier in the references and in the "Availability of Data and Materials" section of your manuscript.<br><br>Have you have met the above requirement as detailed in our Minimum Standards Reporting Checklist?	Yes

Powered by Editorial Manager® and ProduXion Manager® from Aries Systems Corporation

Manuscript

Click here to access/download;Manuscript;GigaScience scTHI Manuscript .pdf

A MAP of tumor-host interactions in glioma at single cell resolution

Francesca Pia Caruso[1,2], Luciano Garofano[1,3], Fulvio D'Angelo[2,3], Kai Yu[4], Fuchou Tang[4], Jinzhou Yuan[5,6], Jing Zhang[3], Luigi Cerulo[1,2], Davide Bedognetti[7], Peter A. Sims[5,6], Mario Suvà[8,9], Xiao-Dong Su[4], Anna Lasorella[3,10], Antonio Iavarone[3,10,11], Michele Ceccarelli[2,12*]

[1]Department of Science and Technologies, Università degli Studi del Sannio, 82100, Benevento, Italy
[2]Bioinformatics Lab, BIOGEM, Ariano Irpino, Italy
[3]Institute for Cancer Genetics, Columbia University, New York (NY), USA
[4]Biomedical Pioneering Innovation Center (BIOPIC), School of Life Sciences, Peking University, Beijing 100871, China
[5]Department of Systems Biology, Columbia University Irving Medical Center, New York, NY, USA
[6]Department of Biochemistry and Molecular Biophysics, Columbia University Irving Medical Center, New York, NY, USA
[7]Cancer Program, Sidra Medicine, Doha, Qatar
[8]Department of Pathology and Center for Cancer Research, Massachusetts General Hospital and Harvard Medical School, Boston, MA, 02114, USA
[9]Broad Institute of Harvard and MIT, Cambridge, MA 02142, USA
[10]Department of Pathology and Cell Biology, Columbia University Medical Center, New York, NY, USA
[11]Department of Neurology, Columbia University Medical Center, New York, NY, USA
[12]Department of Electrical Engineering and Information Technology (DIETI), University of Naples "Federico II", Italy

* To whom correspondence should be addressed. Tel: +390817683787; Email: michele.ceccarelli@unina.it (MC);

ABSTRACT

Background

Single-cell RNA sequencing is the reference technique to characterize the heterogeneity of tumor microenvironment. The composition of the various cell types making up the microenvironment can significantly affect the way in which the immune system activates cancer rejection mechanisms. Understanding the cross-talk signals between immune cells and cancer cells is a fundamental for the identification immuno-oncology therapeutic targets.

Results

We present a novel method, single cell Tumor-Host Interaction tool (`scTHI`), to identify significantly activated ligand-receptor interactions across clusters of cells from single-cell RNA sequencing data. We apply our approach to uncover the ligand-receptor interactions in glioma using six publicly available human glioma datasets encompassing 57,060 gene expression profiles from 71 patients. By leveraging this large-scale collection we show that unexpected cross-talk partners are highly conserved across different datasets in the majority of the tumor samples. This suggests that shared cross-talk mechanisms exist in glioma.

Conclusions

Our results provide a complete map of the active tumor-host interaction pairs in glioma that can be therapeutically exploited to reduce the immunosuppressive action of the microenvironment in brain tumor.

BACKGROUND

The interaction between malignant cells and their microenvironment influences tumor growth and progression, as the immune system can eliminate cancer cells that present neoantigens recognized by receptors of the adaptive immune system or express ligands for activating receptors on innate immune cells [1]. The composition of the various cell types making up the microenvironment can significantly affect the way in which the immune system activates cancer rejection mechanisms [2–4] and influences the response to immune therapies [5, 6]. Therefore, the elucidation of the tumor-host interaction mechanisms plays a crucial role in the understanding of the tumor growth and evolution [7, 8] and in the identification of immuno-oncology therapeutic targets [9]. Immune checkpoint inhibitor (ICI) therapies are aimed to target specific cell-cell interactions between PD1 and PD-L1 or CTLA4 and B7-1/B7-2 [10]. The identification of novel interactions that characterize tumor-types and shape the immune response has also important clinical implications and can also help to better stratify patients [2] and predict response to ICI [11].
Single-cell RNA sequencing is the reference technology for the quantification and phenotyping of tumor microenvironment at high resolution [12, 13] allowing to measure the composition of individual immune/stromal compartments making up the microenvironment. This technique can be also used for a better elucidation of the tumor-host signalling mechanisms [14] and the identification of tissue-specific interactions at an unprecedented spatially-solved level of details [15].
Glioma are characterized by the worst survival among brain tumor malignancies [6]. In particular Glioblastoma (grade IV glioma) is the most frequent type of primary brain tumor being with median survival below 15 months [16]. In glioma, higher mutational load is associated with increased tumor aggressiveness [2]. Myeloid-derived cells, mostly blood-derived macrophages and resident microglia, are the most prevalent immune compartments observed in the microenvironment of GBM [17–19], inhibiting a productive anti-tumor immunity in GBM and excluding T lymphocytes [20]. The elucidation of the active ligand-receptor (L-R) interactions in the cross-talk between tumor cells and their microenvironment can help to identify the mechanisms that the transformed cells in glioma use to recruit this immunosuppressive microenvironment and to discover novel therapeutic targets.

Here we exploit single cell data developing `scTHI`, a novel algorithm and tool to identify the ligand-receptor pairs that modulate the tumor-microenvironment cross-talk in glioma. `scTHI` is based on the hypothesis that when patterns of interaction are active, they are also simultaneously and highly expressed in homogeneous cell populations. We also model the autocrine and paracrine signalling

effects of L-R partners [21]. Interestingly, by collecting the largest collection of single cells dataset available up to date, we show that unexpected cross-talk partners are highly conserved across different datasets in the majority of the tumor samples. This suggests that shared cross-talk mechanisms exist in glioma. Our results provide a complete map of the active tumor-host interaction pairs in glioma that can be therapeutically exploited to reduce the immunosuppressive action of the microenvironment in brain tumor.

DATA DESCRIPTION

single cell datasets

Glioma single cell gene expression profiles were collected from six different dataset of glioma (**Table 1**). We obtained a subset of IDH mutant gliomas including 10,688 cells from 16 patients from two different studies [22, 23]. Later, we will refer to this subset of cell as a unique dataset. High grade glioma profiles were collected from three distinct studies: Darmanis et al. profiled 3,589 cells from 4 patients [24]; Yuan et al. profiled ~ 29,000 cells from 10 patients augmented with two novel specimens (PJ052 and PJ053) following library construction and sequencing described in [17]; Neftel et al. profiled 7,930 cells from 28 patients [25]. We also considered another dataset of 5,603 single cell profiles derived from both LGG and HGG patients (n = 13) [26]. Overall, we collected gene expression profiles of 57,060 cells for a total of 71 glioma patients. The cohort is composed of several tumor histologies, including 7 Oligodendroglioma (4,753 cells), 2 Oligodendroastrocytoma (612 cells), 11 Astrocytoma (9,421 cells) and 50 Glioblastoma (42,274 cells). Gene expression profiles was processed independently for each dataset. The TPM normalized data of the Tirosh et al., Venteicher et al. and Neftel et al. [22, 23, 25] datasets were downloaded from the GEO repositories under accession numbers GSE70630, GSE89567, GSE131928, respectively. Gene expression profiles from Darmanis et al. and Yuan et al. [17, 24] were downloaded from GEO repositories under accession numbers GSE84465 and GSE103224, respectively. While, raw data from Yu et al. [26] were obtained from authors. The last three datasets were further processed applying a library size normalization and logarithmic transformation. Moreover, in order to reduce the drop-out effects, data matrices were also imputed with a Markov Affinity-based graph approach [27]. When the specific information to distinguish malignant cells from non-tumor cells was not available, we analyzed the chromosomal aberrations in each individual cell by averaging expression level along genomic locations as performed by InferCNV [28]. The chromosomal landscape of inferred CNV allows us to identify non-transformed cells, i.e. cells that didn't harbor the typical chromosomal alterations observed in glioma. Altogether we identified 45,550 malignant cells and 11,510 non-malignant cells among datasets.

Table 1: Overview of collected dataset.

Dataset	GEO Accession	N° Tumor Cells	N° Non-Tumor Cells	N° Patients
Tirosh et al. [22, 23]]	GSE70630	4045	302	6
Venteicher et al. [[22, 23]	GSE89567	5284	1057	10
Darmanis et al. [24]	GSE84465	1091	2498	4
Yuan et al. [17]	GSE103224	25056	4194	10
Neftel et al. [25]	GSE131928	6863	1067	28
Yu et al. [26]	na	3211	2392	13
Tot.		45550	11510	71

Ligand-receptor collection

In order to identify potential tumor-host interactions we collected a list of 2,548 pairs of ligands and receptors (**Table S1**) curating publicly available resources [21, 29]. The curated list is composed of known and novel literature-supported interactions and includes both heteromeric and monomeric ligands/receptors mainly related to chemokine, cytokine, growth factors, integrin, TGF and TNF family members, semaphorins, ephrins, Wnt and Notch signalings. The table of interactions is released in the `scTHI` Bioconductor package.

Immune cell type signatures

Cell-type specific signatures and markers were used to infer the cellular identity of non-malignant cell subpopulations. For this purpose, we generated a curated list of 295 signatures of the Immune and Central Nervous Systems integrating data from various sources (**Table S2**). In particular we merged a manual collection of marker genes with a set of signatures available from public databases and published studies, including: (i) a compendium of 64 human cell types signatures including lymphoid, myeloid, stromal, tissue-specific and stem cells, collected from FANTOM5, ENCODE, Blueprint and Gene Expression Omnibus (GEO) data portals ; (ii) a set of markers for 30 immune cell types, including myeloid and lymphoid subpopulations identified from Peripheral Blood Mononuclear Cells (PBMCs) [30]; (iii) a set of Central Nervous System cell signatures including astrocytes, neuron, oligodendrocytes, microglia, and endothelial cells [31]; (iv) a set of 53 signatures corresponding to 26 different cell types [32–35]; (v) two gene expression programs related to microglia and bone marrow-derived macrophages in gliomas [23]. All the 295 signatures are released in the `scTHI` tool.

ANALYSES

We present `scTHI` an R/Bioconductor package to discover L-R interactions in single cells. There have beenseveral attempts in scoring such pairs that are mainly based on the average expression of the gene pairs across cell populations. One recent approach is reported in [14] where the authors score interactions by calculating the product of mean receptor expression and mean ligand expression in the respective cell types under examination. The significance of the interaction is evaluated through a one-sided Wilcoxon rank-sum between the median interaction score across samples. This idea is similar to the original approach reported by the authors of the CellPhoneDB [29] where the mean expression of the gene pair is considered with the constraint that only receptors and ligands expressed in more than 10% of the cells in the analyzed cluster are selected. The significance of the interaction is then evaluated using a random permutation of the samples. Likewise, the approach proposed from Wang et al. [36] evaluated the expression levels of both ligand and receptors and only highly expressed or differentially expressed genes were selected to find significant L-R interaction. To test whether an interaction pair was highly expressed in two populations, Joost et al. [37] used a random sampling approach, selecting only pairs with an expression level above a baseline threshold with a corrected p-value < 0.01. Instead, Halpern et al. [38] computed an enrichment for each interaction based on the z-score of the mean expression of every L-R pair tested. Most of these methods use mean expression as a measure to detect whether an interaction exists. They not considering that single cell RNA-seq expression is heavily influenced by dropout effect and the average introduces bias in L-R detection. Our `scTHI` score is based on the percentage of cells in two clusters where the expression of the L-R genes is ranked at the top of the expression profile of every cell of the cluster. Due to the limitations in mRNA capture affecting scRNA sequencing, the use of ranked expression values to compare gene pairs is more stable than just averaging gene expression, reducing the false positive rate in interaction pairs detection. Furthermore, the choice to use as score the percentage of cells expressing a top-rank L-R pair gives priority to those pairs that are more uniformly expressed in two clusters of cells. Overall, this allows us to discard interaction pairs for which one of the two members is highly expressed and the other is not, which instead would be detected using a score based on the average of the expression. Our score explicitly model paracrine and autocrine effects. Since we are particularly interested in paracrine effects, our score penalizes L-R pairs where both partners are highly expressed in the same cell. We assess the significance of the score using a bootstrap method similar to [29] (see Methods).

The basic workflow of `scTHI` is presented in **Figure 1.** First, we perform a cell-specific identification using the gene set enrichment Mann-Whitney-Wilcoxon Gene Set Test (mww-GST) [39] based on a collection of 295 gene immune and stromal signatures. We adopt mww-GST since it has been proven to perform better than other enrichment analysis in situations of weak and noisy signals, and therefore can be used in the single cell scenarios with the presence of low detection efficiency and drop-out phenomena. The second step of the pipeline is the scoring of the candidate L-R pairs using the

procedure described in the Methods section. Briefly, the method computes for each cluster the percentage of cells where the L-R partners are ranked at the top 20% (this is a tunable parameter). The score is the mean between the two percentages and it prioritizes L-R with paracrine activation removing form the score the autocrine effects (see Methods). The significance of the score is computed by a bootstrap generated null distribution obtained by randomly shuffling the input data.

scTHI is able to recover validated interactions from single cell data

Many putative ligand/receptor interactions could be identified based on quantitative gene expression evaluation. However, only those occurring among cells spatially close to each other could have a real biological functionality. Goltsev et al. observed that the cellular neighborhood has a profound impact on the expression of protein receptors on immune cells [40], highlighting that the spatial resolution of infiltrating immune cells and the cancer cells play a key role in defining tumor heterogeneity. Given these assumptions, we evaluated whether `scTHI` is able to detect interactions occurring between cluster of cells spatially close. For this reason, we used the high quality CITE-seq dataset described in Govek et al., where the spatial architecture of murine splenic cells was resolved [15]. First, we used `scTHI` to identify all cell types composing the murine spleen described by Govek et al., including T cells (cytotoxic, memory, naïve, regulatory and helper), B cells (follicular, naïve and switched memory), dendritic cells (conventional and plasmacytoid), red pulp macrophages, monocytes-derived macrophages, neutrophils, plasma cells, erythrocytes and erythroid progenitors. We used the signatures in `scTHI` (generated for human) converted to their mouse orthologs. We asked if scTHI was able to detect interactions occurring between clusters of spatially close cells. Govek et al. [15] used CODEX to validate some important ligand/receptor interactions occurring among red-pulp macrophages and monocyte-derived macrophages (C1q-Lrp1), red-pulp macrophages and neutrophils (Hebp1-Fpr2), monocyte-derived macrophages and neutrophils (Anxa1-Fpr1). They identified these interactions on the basis of the proximity of the corresponding cells expressing ligand and receptor in both the CITEseq and CODEX data. Interestingly, `scTHI` detected the validated interactions among the top 10 highest scored, without any spatial information, as shown in **Figure S1**. This highlights how our approach, which scored pairs based on rank expression values, is robust and accurate in the identification of relevant L-R interactions.

Map of non-tumor cells in glioma

Gliomas are primary brain tumors characterized by high levels of intratumor heterogeneity and, despite numerous research advances, the difference in tumor microenvironment composition is still not well understood [41]. We collected a single cell glioma dataset integrating six published studies. This allowed us to comprehensively evaluate the composition of the tumor microenvironment,

spanning different molecular and histological subtypes of glioma. Overall, we have 45,550 malignant cells and 11,510 non-malignant cells among datasets. We classified all non-malignant cells using scTHI, however, below we report the percentages of specific cell compartments computed using the datasets where the cells did now undergo any gating or selection strategy. Classification of the non-malignant cells (**Table S3**) showed that the most relevant fraction of cells in the glioma microenvironment were myeloid cells (~57%) divided in macrophages (~45%) and microglia (~12%) followed by glial cells (~19%), vascular cells (~11%), CD8 T cells (~4%) and few subpopulations of other cell types including natural killer, neutrophils, dendritic cells, monocytes, mesenchymal stem cells and others (~9%). As expected, grade IV glioma (GBM) showed the highest percentage of macrophages in their microenvironment (~52% macrophages and ~8% microglia) compared to other histological subtypes (Astrocytoma-macrophages = ~10% and Astrocytoma-microglia = ~36%; OligoAstro-macrophages = ~9% and OligoAstro-microglia = ~21%; Oligodendroglioma-macrophages = ~1% and Oligodendroglioma-microglia = ~31%) (**Figure 2**). Interestingly, switching from more aggressive histological phenotypes (i.e. GBM) to less aggressive ones (i.e. Oligodendroglioma) the relative percentage of macrophages decreases, while the percentage of microglia cells increases. These data are in agreement with the hypothesis that gliomas in the early stages of their development primarily contain brain-resident microglia cells, whereas macrophage phenotype is associated with higher grades [23]. Astrocytoma and glioblastoma patients showed also a relevant fraction of vascular cells (~44% and ~14%, respectively), probably due to increased microvascular proliferation of these high grade tumors compared to oligodendrogliomas. Regarding the lymphoid populations, T cells represent the most abundant fraction, with a greater number of CD8 cells observed in GBM and Oligo-Astrocytoma.

We also evaluated whether there is a significant association between the different cell types composing the microenvironment and the molecular glioma subtypes [42]. We correlated the percentage of cells classified in one of the glioma subtypes with the percentages of non-malignant cell types only for patients in which the cells were not selected with any gate strategy (**Figure S2**). This analysis showed a significant correlation between the Mesenchymal subtype and the presence of macrophages (rho=0.47, p-value=0.015), myeloid-derived suppressor cells (MDSC) (rho=0.56, p-value=0.003), dendritic cells (rho=0.40, p-value=0.039), and astrocytes (rho=0.42, p-value=0.033); Proneural subtypes was significantly associated with the presence of Oligodendrocytes (rho=0.43, p-value=0.028); Classical subtype was significantly associated with the presence of Microglia (rho=0.45, p-value=0.019).

Cross-talk between mesenchymal GBM tumor cells and myeloid cells in the glioma microenvironment

Since cells of myeloid lineage account for about 50-60% of non-neoplastic cells, we first focused on interactions occurring between tumor and myeloid cells, including bone marrow-derived macrophages and microglia. The analysis was performed on the patients with a sufficient number of detected myeloid cells (n = 39 patients). Each patient was tested to identify both paracrine and autocrine interaction pairs. Only significant L-R pairs were kept, with p-value less than 0.05 and the constraint a total `scTHI` score greater than 0.50 in both clusters. Altogether, we detected 368 significant L-R pairs across datasets by filtering out all interactions occurring in fewer than 4 patients. About 80% of detected interactions (298 out of 368) showed a considerable autocrine signaling (**Table S4**). The remaining 20% of the identified interactions (n = 70) showed paracrine signaling, where the interaction genes were preferably expressed on only one of the two clusters (**Table S4**). Several of high-scored interactions occurred specifically in few patients due to the typical heterogeneity of the considered tumor. Interestingly, a relevant fraction of detected pairs (n = 56, about 15%) was shared at least 50% or more of patients, suggesting the presence of common tumor-host signaling mechanisms in glioma. Many of the inferred interactions involved genes of chemokine and cytokine family (e.g. CCL5, CCR1, CCRL2), toll-like receptors (e.g. TLR2, TLR4), transforming growth factor genes, tumor necrosis factor genes, MHC proteins (e.g. HLA-E, CD74), growth factors and their receptors (e.g. EGFR, PDGFB, PDGFC, PDGFRA, IGF1, IGF1R), cell adhesion molecules (e.g. integrins), enzymes with inhibitor activity, and metalloproteinases. Gene ontology enrichment analysis revealed that secreted ligands or activated receptors in tumor cells are typically involved in processes of extracellular matrix remodeling, cell chemotaxis, Notch signaling, axonogenesis and gliogenesis (**Figure S3A and Table S3**). Instead, receptors and ligands detected on myeloid cells modulate biological processes such as leukocyte chemotaxis and migration, cell-cell adhesion, cytokine production, myeloid cell differentiation, reactive oxygen species metabolic processes and regulation of vasculature development (**Figure S3B and Table S5**).

The significant interactions identified running `scTHI` in paracrine mode and most common among all glioma patients, were VCAN-TLR2 (72% of patients) and HBEGF-EGFR (51% of patients) (**Figure 3**). The Versican gene (VCAN) is an extracellular matrix proteoglycan, typically involved in processes of cell adhesion, proliferation, and migration. It is highly expressed in glioma cells where strongly contributes to tumor progression mechanisms [43]. According to these observations, we found VCAN diffusely expressed in the tumor cells of all the datasets considered (**Figure 4)**. The interaction's partner, the Toll-like receptor 2 (TLR2), is a membrane protein expressed on the surface of many cell types, including monocytes and macrophages, and it is involved in pattern recognition signaling pathway and innate immunity activation. TLR2 is highly and specifically expressed only in cells of the microenvironment of myeloid origin, and low expressed in other cell types (**Figure 4B**). The interaction occurring among VCAN and TLR2 represents an effective link between inflammation and tumor progression. Indeed, the Versican protein, released by tumor cells in the extracellular space, binds TLR2 activating multiple cell types in the tumor microenvironment, including myeloid, fibroblasts and endothelial cells, and promotes the production of many proinflammatory cytokines [44, 45]. The

activation of TLR2 downstream signaling pathway also induces the expression of metalloproteinases involved in extracellular matrix degradation to promote tumor expansion [43]. The most common interaction with the receptor on the tumor cells was composed by the pair EGFR and HBEGF, that could tend to promote tumor growth. In fact, the Epidermal Growth Factor Receptor (EGFR) is a tumor-promoting receptor commonly amplified in gliomas, and the EGF-Like growth factor (HBEGF) is a protein highly expressed in regulatory macrophages with immunosuppressive activity [46]. Among other significant interactions of interest identified by `scTHI`, one involves the macrophage migration inhibitory factor MIF and its receptor CD74 (18% of patients), it plays a role in tumorigenesis, exerting pro-tumorigenic effects such as enhancing proliferation, tumor vascularization and inhibition of apoptosis [47].

We also found that multiple significant interaction pairs involved ligands (TGFB1, TGFB2), receptors (TGFBR1, TGFBR2) and regulators (LTBP1, LTBP3) of the TGFb signaling pathway. The TGFb pathway is a known driver of immunosuppression in multiple epithelial tumors and in glioblastoma in which it also drives other hallmarks of aggressiveness (cancer stem cells, migration, and invasion,etc.) leading to poor survival [48, 49].

The identification of interaction pairs in autocrine mode (**Table S4)** revealed that these signaling are much more conserved among patients than paracrine signaling. As expected, some of the paracrine interactions described above were also identified as autocrine, although they have a preferential paracrine directionality. Among the top scored L-R autocrine pairs, there was an interaction between RPS19 and C5AR1 (**Figure S4**), detected in all the glioma patients tested (n = 39). Although this interaction has never been described in the context of glioma, the Ribosomal protein S19 (RPS19) is up-regulated in breast and ovarian cancers, and its interaction with the C5a receptor 1 (C5AR1), expressed on tumor-infiltrating myeloid cells, has an immunosuppressive effect. RPS19 induces the production of anti-inflammatory cytokines, the activation of Th2 and regulatory T cells, and the reduction of infiltrating CD8 T cells into tumors. It was also noted that reducing RPS19 in tumor cells or blocking the C5aR1-RPS19 interaction decreases RPS19-mediated immunosuppression, impairing the tumor growth [50]. These observations could be translated into gliomas, representing another potential therapeutic target.

Cross-talk between proneural GBM tumor cells and oligodendrocytes in the glioma microenvironment

Whereas the microenvironment of mesenchymal GBM was massively enriched by myeloid cells, proneural GBM contained a low number of myeloid cells but exhibited significant infiltration by oligodendrocytes, which alone accounted for about 17% of tumor-infiltrating cells. Altogether we tested 30 tumor samples, among the 71, for which we identified an adequate number of

oligodendrocytes in their microenvironment. Each individual tumor was tested to identify both paracrine and autocrine interaction pairs, and only significant interactions shared in at least 4 patients were considered. Overall, we found 26 paracrine and 126 autocrine interactions (**Table S6**)**.** Gene ontology enrichment analysis revealed that the cross-talk between tumor cells and oligodendrocytes mainly involve signaling pathways related to cell growth and nervous system development, such as axonogenesis, regulation of neurogenesis, extracellular matrix organization, developmental cell growth, gliogenesis, synapse organization (**Table S7**). Indeed, among the top-scored paracrine interactions (**Figure S5**) there were several ligands and receptors involved in cell-cell adhesion, angiogenesis, and tumorigenesis, such as MDK-LRP2 (12 out of 30 patients) and JAM2-JAM3 (11 out of 30 patients).

The MDK-LRP2, which scored as top interaction among GBM tumor cells (ligand) and oligodendrocyte (receptor) is especially intriguing as overexpression of MDK (midkine) has been shown in several human tumors and recently was reported as driver of aberrant proliferation, poor survival and pharmacological resistance in human glioma [40, 51]. In many patients, we also detected as significant the interaction occurring between the Platelet Derived Growth Factor Subunit A (PDGFA) gene and its receptor, PDGFRA (11 out of 30 patients). PDGFA is a classical marker of oligodendrocytes required for normal oligodendrocyte development. However, the overexpression of its receptor is a hallmark of proneural GBM, where it plays a critical role during tumor development and progression [52]. We found that ~72% of cells expressing PDGFRA at the top 20% of the ranked expression profile was classified as Proneural subtype (Fisher's Exact Test p-Value < 2.2e-16 and odds ratio = 5.4). Other L-R pairs were specifically related to the development of the nervous system and promoting neuronal adhesion, i.e. RGMB-NEO1 and SEMA5A-PLXNB3 bindings (both in 11 out of 30 patients). The most shared interaction detected was CNTN2-NRCAM (15 out of 30 patients). The Contactin 2 (CNTN2) gene, also known as Axonal Glycoprotein TAG-1 (TAX-1), is a cell adhesion molecule that plays an important role in axonal elongation, axonal guidance, and neuronal cell migration [53]. The gene is also amplified and aberrantly expressed in glioblastoma, where it is involved in neoplastic glial cell migration and tumorigenesis [54]. TAX-1 binds numerous molecules, among which the Neuronal Cell Adhesion Molecule (NRCAM) gene. In response to contactin binding, NRCAM promotes directional axonal cone growth in fetal nervous system development and mediate neurite outgrowth in the peripheral nervous system [55]. Although less studied, also NRCAM is overexpressed in high-grade astrocytoma and glioblastoma, representing a marker for brain tumor detection and a putative therapeutic target [56].

Cross-talk involving T-cells in glioma

The scTHI classification of non-tumor cells identified subpopulations of CD8 T cells infiltrating the microenvironment of a small subset of patients (n = 8). Although T cells play a fundamental role in

antitumor immunity, glioblastoma is particularly adept at sabotaging immune surveillance causing severe T cell dysfunction, both qualitative and quantitative [57]. To better understand the complex role of T cells in glioma microenvironment, we simultaneously evaluated the cross-talk existing between CD8 T cells and tumor and myeloid cells, respectively.

We first investigated the putative L-R interactions occurring between tumor and CD8 T cells. Altogether, we detected 16 paracrine and 120 autocrine significant L-R pairs (**Table S8**), considering only interactions occurring in more than three patients. Most of the identified interactions involved genes of the major histocompatibility complex Class I, chemokine, interleukins, interferon gamma and Tumor Necrosis Factor (TNF) signaling genes. Among all interaction detected in paracrine mode (**Figure S6**), CADM1-CRTAM was the most common and high-scored L-R pair. The CADM1 gene, also known as TSLC1, was originally identified as a non–small-cell lung cancer tumor suppressor. The gene encodes a cell-surface protein, called Necl-2, which mediates epithelial cell junctions. We found that CRTAM receptor is highly expressed in CD8 cells with respect to other non-tumor cells in 6 out of 8 glioma patients (**Figure S7**). Although recent studies have shown a CADM1 loss at the protein and mRNA levels in high-grade (WHO III/IV) glioma compared to low-grade glioma [58], the fact that we found this interaction mainly in GBM patients in association with infiltrating CD8 T cells further supports the tumor suppressor role of this gene.

Similarly, we analyzed the interactions between T CD8 and myeloid cells, identifying 53 paracrine and 159 autocrine L-R pairs (**Table S9**). The detected interactions mainly involved: (i) chemoattractant chemokine ligands and receptors, like as CXCL16/CXCR6, CXCL12/CXCR3, CLL8/CCR2, CCL5/CCR1, and others.; (ii) immune checkpoint genes, like as CD86, CD28, CTLA-4, LGALS9; (iii) lymphotoxins (LTA and LTB) and other Tumor Necrosis Factor family members, which could modulate T cells immunity through different signaling pathways. The chemokine receptor CXCR6, typically expressed on different T cell compartments, and its ligand CXCL16 secreted by macrophages cells, were found highly expressed in all patients. They induce proliferation and migration of tumor-associated leukocytes affecting cancer cell growth with pro-tumorigenic inflammation [59]. The CXCR3 receptor is also highly expressed on T cells and plays an important role in T cell trafficking and function [60]. Lymphotoxins alfa and beta (LTA and LTB) are cytokines produced by lymphocytes, belonging to the tumor necrosis factor family. Although they were originally identified as lymphocyte products capable of exerting cytotoxic effects on tumor cells in vitro and in vivo, recent studies have shown that LT contributes to several effector responses of both the innate and adaptive immune systems. Binding to both TNFRSF1A/TNFRSF1B and LTβR with high affinity, LT-mediated signaling is essential for the development of secondary lymphoid tissues [61]. Moreover, the activation of LTβR on macrophages by T cell-derived lymphotoxins controls proinflammatory response, inducing cross-tolerance to TLR4 and TLR9 ligands through the downregulation of proinflammatory cytokine and a negative regulation of NF-kB activation induced by TLR signaling [62]. Finally, we also identified two opposite immune checkpoint signals in almost all patients involving CD86 (n = 8) and CTLA-4 (n =

6) to binding CD28. Usually, CD86/CD28 binding results in activation and initiation of T cell effector function. However, high levels of CTLA-4 expression on T cells, probably induced by cancer, creates competition with CD28 and results in insufficient costimulation and a loss of T-cell proliferation and function [57].

DISCUSSION

In this work, we described `scTHI` a novel computational approach to identify active ligand-receptor interactions in single cells and applied to five glioma datasets encompassing 71 patients, 45,550 malignant cells and 11,510 non-malignant cells. We presented a comprehensive map of active tumor-host interactions in glioma (**Figure S8**). We have first shown that scTHI can identify recently discovered interactions validated by CODEX [15] and then explored common ligand-receptor cross-talk in glioma. Our results confirm, using a much larger scale, that myeloid cells make up the bulk of the microenvironment in glioma and that the ratio between macrophages and microglia cell increases with more aggressive phenotypes as has been previously observed for IDH-mutant glioma [23]. Interestingly, the use of a large patient cohort allowed us to link the specific immune compartments with glioma subtypes. Indeed, we report that the presence of macrophages and myeloid-derived suppressor cells was significantly associated with the Mesenchymal subtype, as also described in [63], whereas the Proneural subtype was significantly associated with the presence of Oligodendrocytes, cells in the Classical subtype, on the other hand, tend to associated with the presence of microglia.

Our complete map of cross-talk between tumor and myeloid cells allowed us to identify some known and some novel potential targets for promoting anticancer therapy enhancing the immune response. Although a significant number of interactions are specific to few patients, when collectively analyzed, our findings show that the members of the interactions on the malignant cells enrich common pathways from those on immune cells. The ligand and receptor expressed on cancer cells participate in pathways such as extracellular matrix remodelling, cell chemotaxis, Notch signaling, axonogenesis, and gliogenesis. Instead, receptors and ligands detected on myeloid cells modulate biological processes such as leukocyte chemotaxis and migration, cell-cell adhesion, cytokine production, myeloid cell differentiation, reactive oxygen species metabolic processes and regulation of vasculature development. This pattern underlines a highly connected tumor-host communication network in glioma, where many ligands-receptors can interact on the same cell type.

We have also identified a subset of interactions that are highly conserved across different patients and datasets in paracrine mode showing that TLR2 is specifically and exclusively upregulated in glioma-associated microglia; in contrast, astrocytes and glioma cells expressed only low levels of TLR2. It is known that versican is a glioma-derived endogenous TLR2 mediator that regulates microglial MT1-MMP expression for tumor expansion [45]. Microglial upregulation can be abolished by targeting TLR2 with potential therapeutic benefits in glioma progression [43]. Our results confirm that TLR2 is a candidate for adjuvant therapy in the treatment of glioma [64]. We also described the interaction between EGFR and HBEGF, the feedback loop between these two genes regulates astrocytes maturation [65] and promotes gliomagenesis in specific contexts, the silencing of one of the partners tends to reduce tumor growth and increases survival *in vivo* [66].

Another common interaction across several patients include the pair MIF-CD74. Although MIF is a proinflammatory cytokine, it also exerts immunosuppressive functions, influencing the M1/M2 polarization of tumor-associated macrophages [67]. In fact, recent studies have shown that the MIF-CD74 binding activates a signaling pathway resulting in M2 shift both in microglial cells, macrophages and dendritic cells [68]. The Inhibition of MIF signaling on these cells restore the antitumor immune response leading to a decrease in the expression of immunosuppressive factors and a reacquired capacity in cytotoxic T cells activation [69]. In addition, the CD74 receptor after activation is quickly internalized and recycles, therefore it constitutes an attractive target for anticancer antibody-based treatment strategies. We reported the L-R interaction including THY1 (CD90) and ITGAM/ITGB2 (Mac-1, CD11b/CD18), involved in leukocyte recruitment in response to inflammatory signals (46% of patients). The CD90 gene is a specific surface marker highly expressed in glioma-associated mesenchymal stem cells [70]. The encoded protein drives glioma progression through SRC-dependent mechanisms increasing proliferation, migration, and adhesion [71]. On the other hand, the CD11b/CD18 integrins complex is abundantly expressed on monocyte/macrophages surface, where it is involved in critical adhesive reactions including the recruitment of myeloid cells to the tumor site [72]. Moreover, CD11b is a negative regulator of immune suppression, representing an interesting target for cancer immunotherapy [73]. In fact, CD11b activation promotes pro-inflammatory macrophage polarization, while its inhibition leads to immune suppressive macrophage polarization, vascular maturation, and accelerated tumor growth.

When we applied our algorithm in autocrine mode, we found some common interactions shared by all patients of our cohort. RPS19, for example, is an abundant intracellular protein that is expressed by virtually all cells in the body, and its extracellular functions, including interaction with C5aR1, are activated upon its release from dying cells [74]. The importance of C5aR1-RPS19 interaction for immunosuppression was recently demonstrated showing that the downregulation of RPS19 in tumor cells or pharmacological blockade of C5aR1 by C5aRA reduced this immunosuppression and led to the generation of tumor-specific T cell responses and slower tumor growth in breast and ovarian cancer cells [50]. We have shown that the C5aR1-RPS19 interaction is among the most common signaling cross-talk between tumor cells and their microenvironment across multiple patients making these molecules an interesting target for therapeutic strategies.

Our analysis confirmed that proneural GBMs are significantly infiltrated by oligodendrocytes. MDK resulted in one of the top possible mediators of the interaction between glioma cells and oligodendrocytes through its ligand LRP2. The oncogenic role of MDK, promoting proliferation and pharmacological resistance in glioma, may involve the release of the Sonic Hedgehog (SHH) from LRP2 sequestration in oligodendrocytes [75], thus functioning to activate one of the best-known activator of proliferation and stemness of brain tumors [76]. The single-cell characterization of proneural glioma evidenced that the concurrent expression of high levels of the PDGFA ligand by the abundant oligodendrocytes infiltrating PDGFRA-amplified proneural GBM provide the crucial initiating

signaling event for the effects that this pathway has for proliferation, stemness, and progression of brain tumors [77].

Recent trials have shown that endogenous T cells play a significant role in the prolonged survival time of glioma patients [2, 78]. However, glioblastoma-induced immune suppression is a major obstacle to an effective and durable immune-mediated antitumor response. We have shown that a significant number of glioma patients present subpopulations of CD8 T cells. The presence and T cell clonal diversity in the tumor microenvironment has also been associated with response to immune-therapy in glioma [79]. Our analysis reported that this mechanism of tumor suppression could be mediated by CADM1 and its receptor CRTAM. Typically, CADM1 performs its antitumor activity ensuring that cells grow in organized layers, inhibiting uncontrolled growth. Moreover, Necl-2 binds natural killer and CD8+ T cells through a receptor known as class I-restricted T-cell-associated molecule (CRTAM), which is expressed only on activated cells [58]. The interaction among CRTAM and Necl-2 promotes cytotoxicity of NK cells and interferon gamma (IFN-gamma) secretion of CD8+ T cells in vitro [80].

METHODS

Immune cell type classification

To evaluate the enrichment of each immune cell type in glioma samples we used the collection of 295 signature and Normalized Enrichment Score (NES) of the Mann Whitney Wilcoxon Gene Set test (mww-GST) that we previously described in [39]. Briefly, NES is an estimate of the probability that the expression of a gene in the gene set is greater than the expression of a gene outside this set:

$$NES = 1 - \frac{U}{mn}$$

where m is the number of genes in a gene set, n is the number of those outside the gene set, $U = mn + m(m+1) - T$, and T is the sum of the ranks of the genes in the gene set. We applied single cell mww-GST and classified each cell according to the signature with the highest NES and corrected p-value less than 0.01.

scTHI scores

The scTHI scores are computed between pairs of clusters of single cells assigning a score to each interaction of the table. A typical example is when we have one cluster from the microenvironment (ex. macrophages) and one cluster from the tumor cells. Given a single cell profile G, ranked from the high expressed to the low expressed genes, we call G^T the set of genes in the top of the ranked profile (in all the reported experiments we use the top 20%, the accompanying code allows to select the threshold). Let also call the two cluster A and B, then for every ligand-receptor (L-R) pair of the interaction table we compute the following score:

$$s(L,R) = \frac{1}{2}\left(\frac{1}{|A|}\sum_{G\in A} I_{G^T}(L)\cdot I_{\overline{G^T}}(R) + \frac{1}{|B|}\sum_{G\in B} I_{G^T}(R)\cdot I_{\overline{G^T}}(L)\right)$$

where I is the indicator function

$$I_X(y) = \begin{cases} 1 \text{ if } y \in X \\ 0 \text{ otherwise} \end{cases}$$

Briefly, the score s is the average between two percentages: the percentage of cells in cluster A where the gene L is at the top of the ranked list and gene R is not at the top and vice versa for cluster B in the second percentage, it tends to give a higher score to paracrine interactions (in order to also score autocrine interactions the second term in the product of the two summations in equation can be removed). A null distribution of the interaction score to assign significance is then obtained by a bootstrap procedure shuffling the input data.

Gene Ontology enrichment analysis

The GO category enrichments of ligand and receptor genes detected by `scTHI` analysis were performed using clusterProfiler [81]. Enriched GO terms were filtered using an adjusted p-Value cutoff of 0.0001.

AVAILABILITY OF SOURCE CODE AND REQUIREMENTS

Project name: single cell Tumor Host Interaction

Project home page: https://bioconductor.org/packages/devel/bioc/html/scTHI.html

Operating system(s): Platform independent

Programming language: R

License: GPL-3

AVAILABILITY OF SUPPORTING DATA AND MATERIALS

Imputed matrices for malignant and non-malignant cells will be uploaded on GigaDb according to the submission Guidelines.

LIST OF ABBREVIATIONS

scTHI: single cell Tumor-Host Interaction; ICI: immune checkpoint inhibitor; GBM: Glioblastoma; L-R: ligand-receptor; scRNA: single cell RNA; mww-GST: Mann-Whitney-Wilcoxon Gene Set Test; CITE-seq: Cellular Indexing of Transcriptomes and Epitopes by Sequencing; CODEX: CO-Detection by indexing; MDSC: myeloid-derived suppressor cells; WHO: the World Health Organization; LGG: low grade glioma; HGG: high grade glioma; GEO: Gene Expression Omnibus; CNV: copy number variation; ENCODE: Encyclopedia of DNA Elements; PBMC: Peripheral Blood Mononuclear Cells; NES: Normalized Enrichment Score; GO: Gene Ontology.

COMPETING INTERESTS

The authors declare no competing interests.

FUNDING

The research leading to these results has received funding from Associazione Italiana per la Ricerca sul Cancro (AIRC) under IG 2018 - ID. 21846 project.

AUTHORS' CONTRIBUTION

- Conceptualization: FPC, AI, MC
- Methodology: FPC, JZ, LG, AI, MC
- Software: FPC, LC, FDA
- Resources: KY, FT, JY, PS, MS, XDS, AL
- Data Curation: KY, FT, JY, PS, MS, XDS, AL
- Writing – Review & Editing: FPC, MS, AI, MC
- Supervision: AI, MC
- Funding Acquisition: MC

TABLE AND FIGURES LEGENDS

Table S1. Ligand-Receptor interactions. List of Ligand-Receptor interaction pairs provided in `scTHI`.

Table S2. Signatures. List of immune system and tissue cell type signatures provided in `scTHI`.

Table S3. Non tumor cell classification. Phenotype classification of tumor microenvironment cells in glioma datasets performed by TME.classification function provided by `scTHI`.

Table S4. Tumor-Myeloid cell interactions. List of significant paracrine and autocrine L-R interactions occurring between tumor and myeloid cells from considered datasets detected by `scTHI`.

Table S5. Enriched GO categories of L-R partners in tumor and myeloid cells. List of significant enriched GO biological processes of ligand and receptor genes expressed in tumor and myeloid cells, respectively.

Table S6. Tumor-Oligodendrocyte cell interactions. List of significant paracrine and autocrine L-R interactions occurring between tumor and oligodendrocyte cells from considered datasets detected by `scTHI`.

Table S7. Enriched GO categories of L-R partners in tumor and oligodendrocyte cells. List of significant enriched GO biological processes of ligand and receptor genes expressed in tumor and oligodendrocyte cells, respectively.

Table S8. Tumor-CD8 cell interactions. List of significant paracrine and autocrine L-R interactions occurring between tumor and CD8 T cells detected by `scTHI`.

Table S9. Myeloid-CD8 cell interactions. List of significant paracrine and autocrine L-R interactions occurring between myeloid and CD8 T cells detected by `scTHI`.

Figure 1. scTHI workflow. Description of scTHI functionalities. (A) In the first step a single sample enrichment analysis based on the Mann-Witney-Wilcoxon Gene Set Test (mww-GST) [39] with a collection of 295 gene immune and stromal signatures is used to nominate the identity of cells of the microenvironment. (B) In the second step, given two clusters of cells, the most significant L-R interactions are identified by assigning a score to every all the 2,548 collected pairs of ligands and receptors.

Figure 2. Tumor microenvironment cell type classification in glioma. The barplots show the relative percentage (A) and the number of cells (B) of each cell type identified in the microenvironment of the main histological subtypes of glioma.

Figure 3. Paracrine tumor-myeloid cell interactions. Barplots show significant paracrine L-R interactions (*p-Value* <= 0.05 and scTHI score >= 0.50) occurring between tumor and myeloid cells shared in at least 4 patients. On the x axis are shown the number of patients where each interaction occurred. (A) Interaction pairs in which the ligand is expressed on tumor cells and the receptor on

myeloid cells. (B) Interaction pairs in which the ligand is expressed on myeloid cells and the receptor on tumor cells.

Figure 4. VCAN-TLR2 interaction. TSNE plots of tumor and non-tumor cells for each dataset analyzed. (A) Each cell is colored by patient (on the right non-tumor and on the left tumor cells). (B) Each cell is colored according to expression value of the genes VCAN and TLR2 in tumor and non tumor cells, respectively. (C) tSNE plot of non-tumor cells colored according to cell type classification.

Figure S1. scTHI recovers validated interactions. The barplots show the top 15 significant interactions identified between (A) red-pulp and monocyte-derived macrophage cells; (B) red-pulp macrophages and neutrophils; (C) monocyte-derived macrophages and neutrophils. The interactions spatially resolved in ([15] are pointed out with an arrow.

Figure S2. Association between immune cell types and glioma subtypes. Heatmap of correlation between the percentage of cells classified in one of the glioma subtypes with the percentages of non tumor cell types. Significant correlations are marker with * (p-value <= 0.05) and ** (p-value <= 0.01).

Figure S3. Enriched GO categories of L-R pair. Network of most represented biological process category enriched by L-R partners on tumor (A) and on myeloid cells (B)**.**

Figure S4. RPS19-C5AR1 interaction. TSNE plots of tumor and non-tumor cells for each dataset analyzed. (A) Each cell is colored by patient (on the right non-tumor and on the left tumor cells). (B) Each cell is colored according to expression value of the genes RPS19 and C5AR1 in tumor and non tumor cells, respectively. (C) tSNE plot of non-tumor cells colored according to cell type classification.

Figure S5. Paracrine tumor-oligodendrocyte cell interactions. Barplots show significant paracrine L-R interactions (*p-Value* <= 0.05 and scTHI score >= 0.50) occurring between tumor and oligodendrocyte cells shared in at least 4 patients. On the x axis are shown the number of patients where each interaction occurred. (A) Interaction pairs in which the ligand is expressed on tumor cells and the receptor on oligodendrocyte cells. (B) Interaction pairs in which the ligand is expressed on oligodendrocyte cells and the receptor on tumor cells.

Figure S6. Paracrine tumor-CD8 cell interactions. Barplots show significant paracrine L-R interactions (*p-Value* <= 0.05 and scTHI score >= 0.50) occurring between tumor and CD8 cells shared in at least 4 patients. On the x axis the number of patients where each interaction occurred is shown. (A) Interaction pairs in which the ligand is expressed on tumor cells and the receptor on CD8 cells. (B) Interaction pairs in which the ligand is expressed on CD8 cells and the receptor on tumor cells.

Figure S7. CADM1-CRTAM interaction. TSNE plots of tumor and non-tumor cells for each dataset analyzed. (A) Each cell is colored by patient (on the right non-tumor and on the left tumor cells). (B) Each cell is colored according to expression value of the genes CADM1 and CRTAM in tumor and non tumor cells, respectively. (C) tSNE plot of non-tumor cells colored according to cell type classification.

Figure S8. A map of tumor-host interactions in glioma. Chord diagram of paracrine tumor-host interaction detected by scTHI. The color of the arcs indicates the clusters of cells among which the interaction has been identified. The origin of the arch indicates that the ligand or receptor is expressed on the tumor, instead the arrow of the arch indicates that the ligand or receptor is expressed on the cells of the microenvironment.

REFERENCES

1. Schreiber RD, Old LJ, Smyth MJ. Cancer immunoediting: integrating immunity's roles in cancer suppression and promotion. Science. 2011;331:1565–70. doi:10.1126/science.1203486.

2. Zhang J, Caruso FP, Sa JK, Justesen S, Nam D-H, Sims P, et al. The combination of neoantigen quality and T lymphocyte infiltrates identifies glioblastomas with the longest survival. Commun Biol. 2019;2:135. doi:10.1038/s42003-019-0369-7.

3. Bedognetti D, Hendrickx W, Ceccarelli M, Miller LD, Seliger B. Disentangling the relationship between tumor genetic programs and immune responsiveness. Curr Opin Immunol. 2016;39:150–8. doi:10.1016/j.coi.2016.02.001.

4. Joyce JA, Fearon DT. T cell exclusion, immune privilege, and the tumor microenvironment. Science. 2015;348:74–80. doi:10.1126/science.aaa6204.

5. Cristescu R, Mogg R, Ayers M, Albright A, Murphy E, Yearley J, et al. Pan-tumor genomic biomarkers for PD-1 checkpoint blockade-based immunotherapy. Science. 2018;362. doi:10.1126/science.aar3593.

6. Ceccarelli M, Barthel FP, Malta TM, Sabedot TS, Salama SR, Murray BA, et al. Molecular profiling reveals biologically discrete subsets and pathways of progression in diffuse glioma. Cell. 2016;164:550–63. doi:10.1016/j.cell.2015.12.028.

7. Angelova M, Mlecnik B, Vasaturo A, Bindea G, Fredriksen T, Lafontaine L, et al. Evolution of Metastases in Space and Time under Immune Selection. Cell. 2018;175:751-765.e16. doi:10.1016/j.cell.2018.09.018.

8. Bedognetti D, Ceccarelli M, Galluzzi L, Lu R, Palucka K, Samayoa J, et al. Toward a comprehensive view of cancer immune responsiveness: a synopsis from the SITC workshop. J Immunother Cancer. 2019;7:131. doi:10.1186/s40425-019-0602-4.

9. Hoos A. Development of immuno-oncology drugs - from CTLA4 to PD1 to the next generations. Nat Rev Drug Discov. 2016;15:235–47. doi:10.1038/nrd.2015.35.

10. Pardoll DM. The blockade of immune checkpoints in cancer immunotherapy. Nat Rev Cancer. 2012;12:252–64. doi:10.1038/nrc3239.

11. Roelands J, Hendrickx W, Mall R, Saad M, Halliwill K, Zoppoli G, et al. Genomic landscape of tumor-host interactions with differential prognostic and predictive connotations. BioRxiv. 2019. doi:10.1101/546069.

12. Svensson V, Vento-Tormo R, Teichmann SA. Exponential scaling of single-cell RNA-seq in the past decade. Nat Protoc. 2018;13:599–604. doi:10.1038/nprot.2017.149.

13. Azizi E, Carr AJ, Plitas G, Cornish AE, Konopacki C, Prabhakaran S, et al. Single-Cell Map of Diverse Immune Phenotypes in the Breast Tumor Microenvironment. Cell. 2018;174:1293-1308.e36. doi:10.1016/j.cell.2018.05.060.

14. Kumar MP, Du J, Lagoudas G, Jiao Y, Sawyer A, Drummond DC, et al. Analysis of Single-Cell RNA-Seq Identifies Cell-Cell Communication Associated with Tumor Characteristics. Cell Rep. 2018;25:1458-1468.e4. doi:10.1016/j.celrep.2018.10.047.

15. Govek KW, Troisi EC, Woodhouse S, Camara PG. Single-Cell Transcriptomic Analysis of mIHC Images via Antigen Mapping. BioRxiv. 2019. doi:10.1101/672501.

16. Stupp R, Mason WP, van den Bent MJ, Weller M, Fisher B, Taphoorn MJB, et al. Radiotherapy

plus concomitant and adjuvant temozolomide for glioblastoma. N Engl J Med. 2005;352:987–96. doi:10.1056/NEJMoa043330.

17. Yuan J, Levitin HM, Frattini V, Bush EC, Boyett DM, Samanamud J, et al. Single-cell transcriptome analysis of lineage diversity in high-grade glioma. Genome Med. 2018;10:57. doi:10.1186/s13073-018-0567-9.

18. Wang Q, Hu B, Hu X, Kim H, Squatrito M, Scarpace L, et al. Tumor Evolution of Glioma-Intrinsic Gene Expression Subtypes Associates with Immunological Changes in the Microenvironment. Cancer Cell. 2018;33:152. doi:10.1016/j.ccell.2017.12.012.

19. Quail DF, Joyce JA. The microenvironmental landscape of brain tumors. Cancer Cell. 2017;31:326–41. doi:10.1016/j.ccell.2017.02.009.

20. Hussain SF, Yang D, Suki D, Aldape K, Grimm E, Heimberger AB. The role of human glioma-infiltrating microglia/macrophages in mediating antitumor immune responses. Neuro Oncol. 2006;8:261–79. doi:10.1215/15228517-2006-008.

21. Ramilowski JA, Goldberg T, Harshbarger J, Kloppmann E, Lizio M, Satagopam VP, et al. A draft network of ligand-receptor-mediated multicellular signalling in human. Nat Commun. 2015;6:7866. doi:10.1038/ncomms8866.

22. Tirosh I, Venteicher AS, Hebert C, Escalante LE, Patel AP, Yizhak K, et al. Single-cell RNA-seq supports a developmental hierarchy in human oligodendroglioma. Nature. 2016;539:309–13. doi:10.1038/nature20123.

23. Venteicher AS, Tirosh I, Hebert C, Yizhak K, Neftel C, Filbin MG, et al. Decoupling genetics, lineages, and microenvironment in IDH-mutant gliomas by single-cell RNA-seq. Science. 2017;355. doi:10.1126/science.aai8478.

24. Darmanis S, Sloan SA, Croote D, Mignardi M, Chernikova S, Samghababi P, et al. Single-Cell RNA-Seq Analysis of Infiltrating Neoplastic Cells at the Migrating Front of Human Glioblastoma. Cell Rep. 2017;21:1399–410. doi:10.1016/j.celrep.2017.10.030.

25. Neftel C, Laffy J, Filbin MG, Hara T, Shore ME, Rahme GJ, et al. An integrative model of cellular states, plasticity, and genetics for glioblastoma. Cell. 2019;178:835-849.e21. doi:10.1016/j.cell.2019.06.024.

26. Yu K, Hu Y, Wu F, Guo Q, Qian Z, Hu W, et al. Surveying Brain Tumor Heterogeneity by Single-Cell RNA Sequencing of Multi-sector Biopsies. BioRxiv. 2020. doi:10.1101/2020.01.19.911701.

27. van Dijk D, Sharma R, Nainys J, Yim K, Kathail P, Carr AJ, et al. Recovering Gene Interactions from Single-Cell Data Using Data Diffusion. Cell. 2018;174:716-729.e27. doi:10.1016/j.cell.2018.05.061.

28. Patel AP, Tirosh I, Trombetta JJ, Shalek AK, Gillespie SM, Wakimoto H, et al. Single-cell RNA-seq highlights intratumoral heterogeneity in primary glioblastoma. Science. 2014;344:1396–401. doi:10.1126/science.1254257.

29. Vento-Tormo R, Efremova M, Botting RA, Turco MY, Vento-Tormo M, Meyer KB, et al. Single-cell reconstruction of the early maternal-fetal interface in humans. Nature. 2018;563:347–53. doi:10.1038/s41586-018-0698-6.

30. Butler A, Hoffman P, Smibert P, Papalexi E, Satija R. Integrating single-cell transcriptomic data across different conditions, technologies, and species. Nat Biotechnol. 2018;36:411–20. doi:10.1038/nbt.4096.

31. Zhang Y, Chen K, Sloan SA, Bennett ML, Scholze AR, O'Keeffe S, et al. An RNA-sequencing transcriptome and splicing database of glia, neurons, and vascular cells of the cerebral cortex. J Neurosci. 2014;34:11929–47. doi:10.1523/JNEUROSCI.1860-14.2014.

32. Bindea G, Mlecnik B, Tosolini M, Kirilovsky A, Waldner M, Obenauf AC, et al. Spatiotemporal dynamics of intratumoral immune cells reveal the immune landscape in human cancer. Immunity. 2013;39:782–95. doi:10.1016/j.immuni.2013.10.003.

33. Charoentong P, Finotello F, Angelova M, Mayer C, Efremova M, Rieder D, et al. Pan-cancer Immunogenomic Analyses Reveal Genotype-Immunophenotype Relationships and Predictors of Response to Checkpoint Blockade. Cell Rep. 2017;18:248–62. doi:10.1016/j.celrep.2016.12.019.

34. Rooney MS, Shukla SA, Wu CJ, Getz G, Hacohen N. Molecular and genetic properties of tumors associated with local immune cytolytic activity. Cell. 2015;160:48–61. doi:10.1016/j.cell.2014.12.033.

35. Tirosh I, Izar B, Prakadan SM, Wadsworth MH, Treacy D, Trombetta JJ, et al. Dissecting the multicellular ecosystem of metastatic melanoma by single-cell RNA-seq. Science. 2016;352:189–96. doi:10.1126/science.aad0501.

36. Wang Y, Wang R, Zhang S, Song S, Jiang C, Han G, et al. iTALK: an R Package to Characterize and Illustrate Intercellular Communication. BioRxiv. 2019. doi:10.1101/507871.

37. Joost S, Jacob T, Sun X, Annusver K, La Manno G, Sur I, et al. Single-Cell Transcriptomics of Traced Epidermal and Hair Follicle Stem Cells Reveals Rapid Adaptations during Wound Healing. Cell Rep. 2018;25:585-597.e7. doi:10.1016/j.celrep.2018.09.059.

38. Halpern KB, Shenhav R, Massalha H, Toth B, Egozi A, Massasa EE, et al. Paired-cell sequencing enables spatial gene expression mapping of liver endothelial cells. Nat Biotechnol. 2018;36:962–70. doi:10.1038/nbt.4231.

39. Frattini V, Pagnotta SM, Tala, Fan JJ, Russo MV, Lee SB, et al. A metabolic function of FGFR3-TACC3 gene fusions in cancer. Nature. 2018;553:222–7. doi:10.1038/nature25171.

40. Lorente M, Torres S, Salazar M, Carracedo A, Hernández-Tiedra S, Rodríguez-Fornés F, et al. Stimulation of the midkine/ALK axis renders glioma cells resistant to cannabinoid antitumoral action. Cell Death Differ. 2011;18:959–73. doi:10.1038/cdd.2010.170.

41. Chen Z, Hambardzumyan D. Immune microenvironment in glioblastoma subtypes. Front Immunol. 2018;9:1004. doi:10.3389/fimmu.2018.01004.

42. Wang Q, Hu B, Hu X, Kim H, Squatrito M, Scarpace L, et al. Tumor Evolution of Glioma-Intrinsic Gene Expression Subtypes Associates with Immunological Changes in the Microenvironment. Cancer Cell. 2017;32:42-56.e6. doi:10.1016/j.ccell.2017.06.003.

43. Hu F, Dzaye OD, Hahn A, Yu Y, Scavetta RJ, Dittmar G, et al. Glioma-derived versican promotes tumor expansion via glioma-associated microglial/macrophages Toll-like receptor 2 signaling. Neuro Oncol. 2015;17:200–10. doi:10.1093/neuonc/nou324.

44. Wang W, Xu G-L, Jia W-D, Ma J-L, Li J-S, Ge Y-S, et al. Ligation of TLR2 by versican: a link between inflammation and metastasis. Arch Med Res. 2009;40:321–3. doi:10.1016/j.arcmed.2009.04.005.

45. Kim S, Takahashi H, Lin W-W, Descargues P, Grivennikov S, Kim Y, et al. Carcinoma-produced factors activate myeloid cells through TLR2 to stimulate metastasis. Nature. 2009;457:102–6. doi:10.1038/nature07623.

46. Edwards JP, Zhang X, Mosser DM. The expression of heparin-binding epidermal growth factor-like growth factor by regulatory macrophages. J Immunol. 2009;182:1929–39. doi:10.4049/jimmunol.0802703.

47. Fukaya R, Ohta S, Yaguchi T, Matsuzaki Y, Sugihara E, Okano H, et al. MIF Maintains the Tumorigenic Capacity of Brain Tumor-Initiating Cells by Directly Inhibiting p53. Cancer Res. 2016;76:2813–23. doi:10.1158/0008-5472.CAN-15-1011.

48. Anido J, Sáez-Borderías A, Gonzàlez-Juncà A, Rodón L, Folch G, Carmona MA, et al. TGF-β

Receptor Inhibitors Target the CD44(high)/Id1(high) Glioma-Initiating Cell Population in Human Glioblastoma. Cancer Cell. 2010;18:655–68. doi:10.1016/j.ccr.2010.10.023.

49. Kaminska B, Kocyk M, Kijewska M. TGF beta signaling and its role in glioma pathogenesis. Adv Exp Med Biol. 2013;986:171–87. doi:10.1007/978-94-007-4719-7_9.

50. Markiewski MM, Vadrevu SK, Sharma SK, Chintala NK, Ghouse S, Cho J-H, et al. The ribosomal protein S19 suppresses antitumor immune responses via the complement c5a receptor 1. J Immunol. 2017;198:2989–99. doi:10.4049/jimmunol.1602057.

51. Cheng Y-P, Lin C, Lin P-Y, Cheng C-Y, Ma H-I, Chen C-M, et al. Midkine expression in high grade gliomas: Correlation of this novel marker with proliferation and survival in human gliomas. Surg Neurol Int. 2014;5:78. doi:10.4103/2152-7806.133205.

52. Song K, Yuan Y, Lin Y, Wang Y-X, Zhou J, Gai Q-J, et al. ERBB3, IGF1R, and TGFBR2 expression correlate with PDGFR expression in glioblastoma and participate in PDGFR inhibitor resistance of glioblastoma cells. Am J Cancer Res. 2018;8:792–809.

53. Masuda T. Contactin-2/TAG-1, active on the front line for three decades. Cell Adh Migr. 2017;11:524–31. doi:10.1080/19336918.2016.1269998.

54. Rickman DS, Tyagi R, Zhu XX, Bobek MP, Song S, Blaivas M, et al. The gene for the axonal cell adhesion molecule TAX-1 is amplified and aberrantly expressed in malignant gliomas. Cancer Res. 2001;61:2162–8.

55. Lustig M, Sakurai T, Grumet M. Nr-CAM promotes neurite outgrowth from peripheral ganglia by a mechanism involving axonin-1 as a neuronal receptor. Dev Biol. 1999;209:340–51. doi:10.1006/dbio.1999.9250.

56. Sehgal A, Boynton AL, Young RF, Vermeulen SS, Yonemura KS, Kohler EP, et al. Cell adhesion molecule Nr-CAM is over-expressed in human brain tumors. Int J Cancer. 1998;76:451–8. doi:10.1002/(sici)1097-0215(19980518)76:4<451::aid-ijc1>3.0.co;2-q.

57. Woroniecka KI, Rhodin KE, Chongsathidkiet P, Keith KA, Fecci PE. T-cell Dysfunction in Glioblastoma: Applying a New Framework. Clin Cancer Res. 2018;24:3792–802. doi:10.1158/1078-0432.CCR-18-0047.

58. Houshmandi SS, Surace EI, Zhang HB, Fuller GN, Gutmann DH. Tumor suppressor in lung cancer-1 (TSLC1) functions as a glioma tumor suppressor. Neurology. 2006;67:1863–6. doi:10.1212/01.wnl.0000244472.56198.84.

59. Darash-Yahana M, Gillespie JW, Hewitt SM, Chen Y-YK, Maeda S, Stein I, et al. The chemokine CXCL16 and its receptor, CXCR6, as markers and promoters of inflammation-associated cancers. PLoS ONE. 2009;4:e6695. doi:10.1371/journal.pone.0006695.

60. Sottili M, Cosmi L, Borgogni E, Sarchielli E, Maggi L, Francalanci M, et al. Immunomodulatory effects of BXL-01-0029, a less hypercalcemic vitamin D analogue, in human cardiomyocytes and T cells. Exp Cell Res. 2009;315:264–73. doi:10.1016/j.yexcr.2008.10.025.

61. Upadhyay V, Fu Y-X. Lymphotoxin signalling in immune homeostasis and the control of microorganisms. Nat Rev Immunol. 2013;13:270–9. doi:10.1038/nri3406.

62. Wimmer N, Huber B, Barabas N, Röhrl J, Pfeffer K, Hehlgans T. Lymphotoxin β receptor activation on macrophages induces cross-tolerance to TLR4 and TLR9 ligands. J Immunol. 2012;188:3426–33. doi:10.4049/jimmunol.1103324.

63. Müller S, Kohanbash G, Liu SJ, Alvarado B, Carrera D, Bhaduri A, et al. Single-cell profiling of human gliomas reveals macrophage ontogeny as a basis for regional differences in macrophage activation in the tumor microenvironment. Genome Biol. 2017;18:234. doi:10.1186/s13059-017-1362-4.

64. Wenger KJ, Wagner M, You S-J, Franz K, Harter PN, Burger MC, et al. Bevacizumab as a last-line treatment for glioblastoma following failure of radiotherapy, temozolomide and lomustine. Oncol Lett. 2017;14:1141–6. doi:10.3892/ol.2017.6251.

65. Li J, Khankan RR, Caneda C, Godoy MI, Haney MS, Krawczyk MC, et al. Astrocyte-to-astrocyte contact and a positive feedback loop of growth factor signaling regulate astrocyte maturation. Glia. 2019;67:1571–97. doi:10.1002/glia.23630.

66. Shin CH, Robinson JP, Sonnen JA, Welker AE, Yu DX, VanBrocklin MW, et al. HBEGF promotes gliomagenesis in the context of Ink4a/Arf and Pten loss. Oncogene. 2017;36:4610–8. doi:10.1038/onc.2017.83.

67. Zeiner PS, Preusse C, Blank A-E, Zachskorn C, Baumgarten P, Caspary L, et al. MIF Receptor CD74 is Restricted to Microglia/Macrophages, Associated with a M1-Polarized Immune Milieu and Prolonged Patient Survival in Gliomas. Brain Pathol. 2015;25:491–504. doi:10.1111/bpa.12194.

68. Ghoochani A, Schwarz MA, Yakubov E, Engelhorn T, Doerfler A, Buchfelder M, et al. MIF-CD74 signaling impedes microglial M1 polarization and facilitates brain tumorigenesis. Oncogene. 2016;35:6246–61. doi:10.1038/onc.2016.160.

69. Figueiredo CR, Azevedo RA, Mousdell S, Resende-Lara PT, Ireland L, Santos A, et al. Blockade of MIF-CD74 Signalling on Macrophages and Dendritic Cells Restores the Antitumour Immune Response Against Metastatic Melanoma. Front Immunol. 2018;9:1132. doi:10.3389/fimmu.2018.01132.

70. Zhang Q, Yi D-Y, Xue B-Z, Wen W-W, Lu Y-P, Abdelmaksou A, et al. CD90 determined two subpopulations of glioma-associated mesenchymal stem cells with different roles in tumour progression. Cell Death Dis. 2018;9:1101. doi:10.1038/s41419-018-1140-6.

71. Avril T, Etcheverry A, Pineau R, Obacz J, Jegou G, Jouan F, et al. CD90 expression controls migration and predicts dasatinib response in glioblastoma. Clin Cancer Res. 2017;23:7360–74. doi:10.1158/1078-0432.CCR-17-1549.

72. Podolnikova NP, Kushchayeva YS, Wu Y, Faust J, Ugarova TP. The Role of Integrins αMβ2 (Mac-1, CD11b/CD18) and αDβ2 (CD11d/CD18) in Macrophage Fusion. Am J Pathol. 2016;186:2105–16. doi:10.1016/j.ajpath.2016.04.001.

73. Schmid MC, Khan SQ, Kaneda MM, Pathria P, Shepard R, Louis TL, et al. Integrin CD11b activation drives anti-tumor innate immunity. Nat Commun. 2018;9:5379. doi:10.1038/s41467-018-07387-4.

74. Yamamoto T. Roles of the ribosomal protein S19 dimer and the C5a receptor in pathophysiological functions of phagocytic leukocytes. Pathol Int. 2007;57:1–11. doi:10.1111/j.1440-1827.2007.02049.x.

75. Christ A, Christa A, Kur E, Lioubinski O, Bachmann S, Willnow TE, et al. LRP2 is an auxiliary SHH receptor required to condition the forebrain ventral midline for inductive signals. Dev Cell. 2012;22:268–78. doi:10.1016/j.devcel.2011.11.023.

76. Niyaz M, Khan MS, Mudassar S. Hedgehog signaling: an achilles' heel in cancer. Transl Oncol. 2019;12:1334–44. doi:10.1016/j.tranon.2019.07.004.

77. Martinho O, Longatto-Filho A, Lambros MBK, Martins A, Pinheiro C, Silva A, et al. Expression, mutation and copy number analysis of platelet-derived growth factor receptor A (PDGFRA) and its ligand PDGFA in gliomas. Br J Cancer. 2009;101:973–82. doi:10.1038/sj.bjc.6605225.

78. Brown CE, Alizadeh D, Starr R, Weng L, Wagner JR, Naranjo A, et al. Regression of Glioblastoma after Chimeric Antigen Receptor T-Cell Therapy. N Engl J Med. 2016;375:2561–9. doi:10.1056/NEJMoa1610497.

79. Zhao J, Chen AX, Gartrell RD, Silverman AM, Aparicio L, Chu T, et al. Immune and genomic correlates of response to anti-PD-1 immunotherapy in glioblastoma. Nat Med. 2019;25:462–9. doi:10.1038/s41591-019-0349-y.

80. Boles KS, Barchet W, Diacovo T, Cella M, Colonna M. The tumor suppressor TSLC1/NECL-2 triggers NK-cell and CD8+ T-cell responses through the cell-surface receptor CRTAM. Blood. 2005;106:779–86. doi:10.1182/blood-2005-02-0817.

81. Yu G, Wang L-G, Han Y, He Q-Y. clusterProfiler: an R package for comparing biological themes among gene clusters. OMICS. 2012;16:284–7. doi:10.1089/omi.2011.0118.

Figure 1

Click here to access/download;Figure;Figure1.pdf

Figure 1

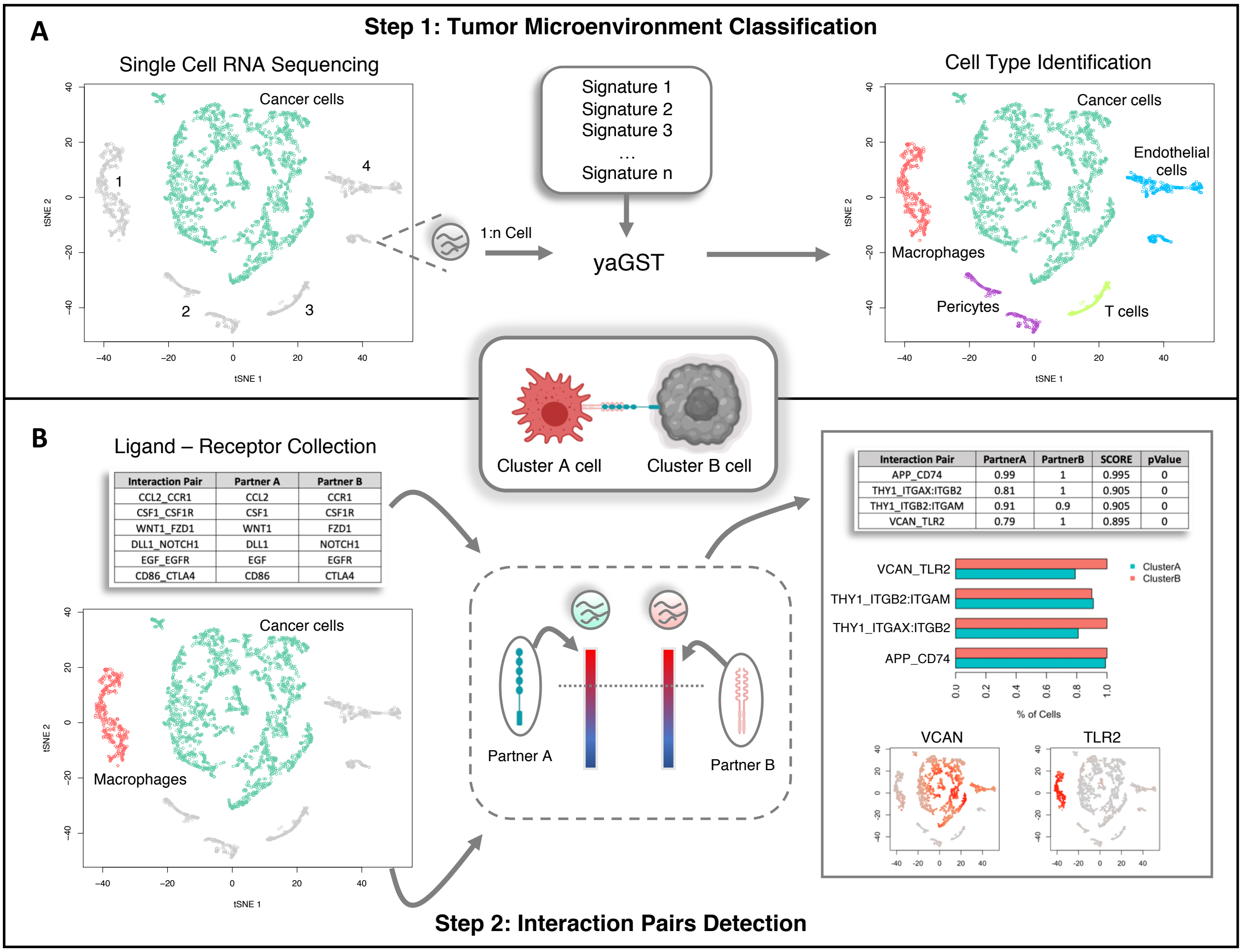

Figure 2

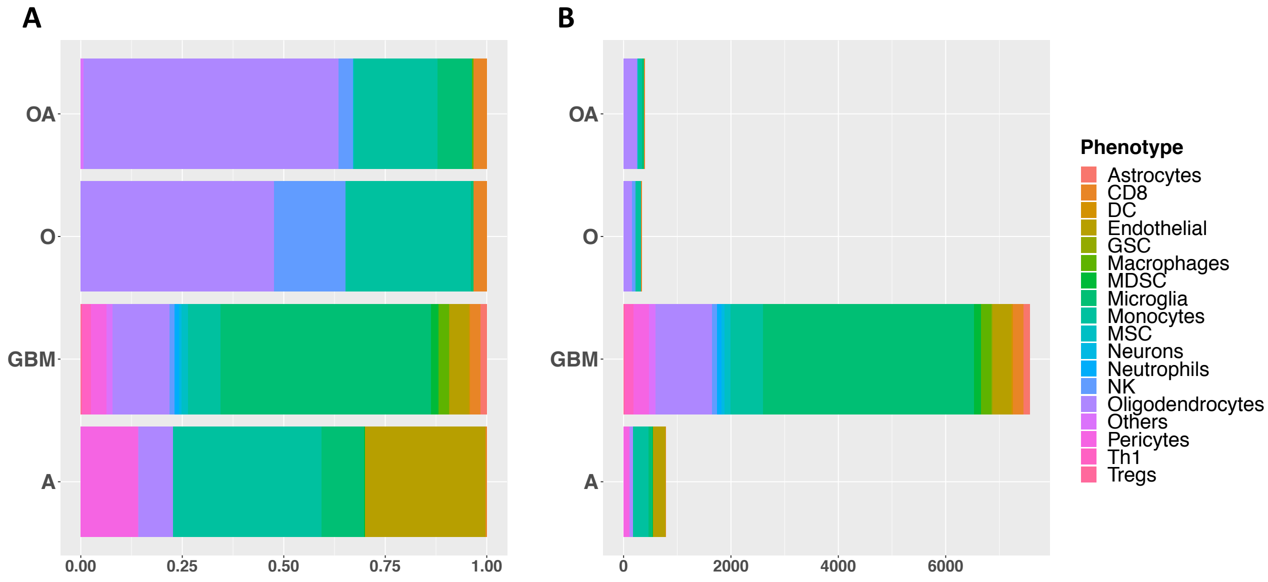

Figure 2. Tumor microenvironment cell type classification in glioma. The barplots show the relative percentage (A) and the number of cells (B) of each cell type identified in the microenvironment of the main histological subtypes of glioma.

Figure 3

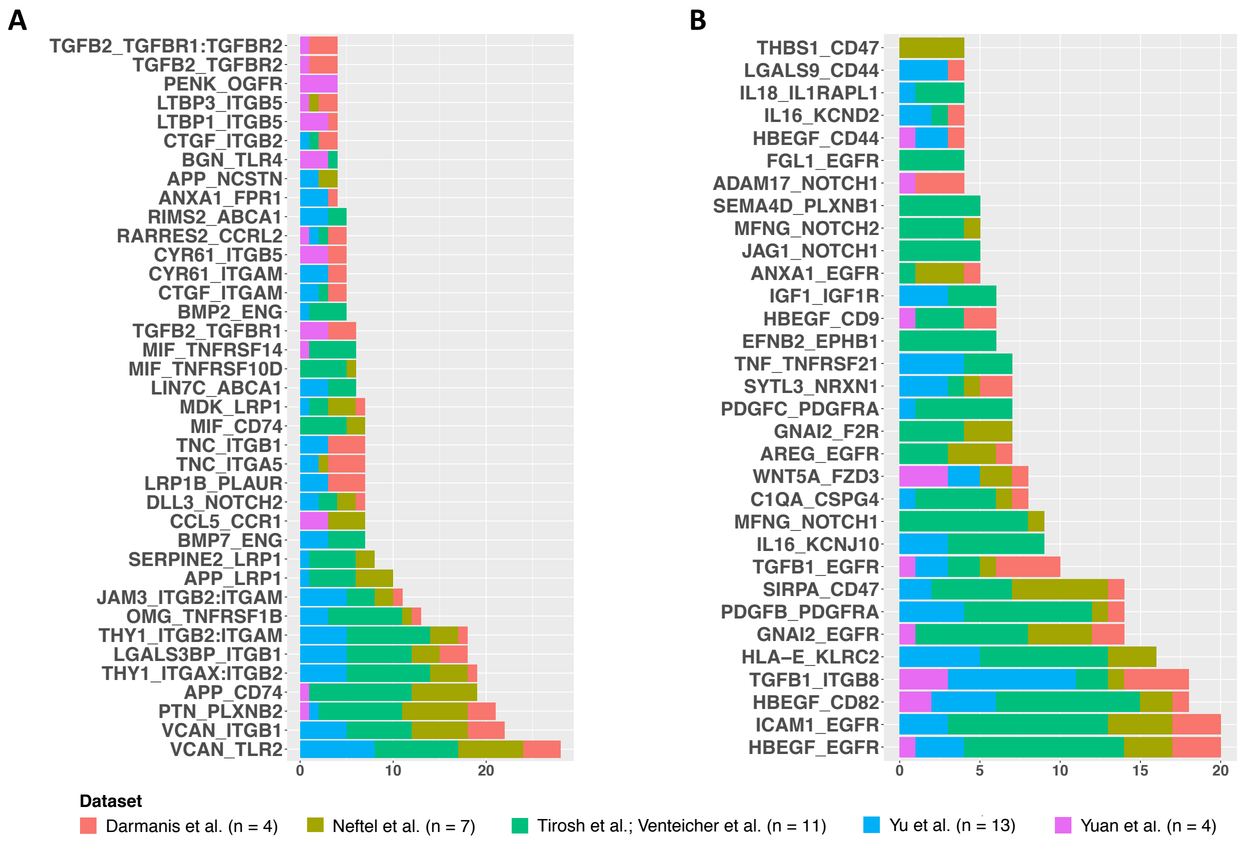

A
TGFB2_TGFBR1:TGFBR2
TGFB2_TGFBR2
PENK_OGFR
LTBP3_ITGB5
LTBP1_ITGB5
CTGF_ITGB2
BGN_TLR4
APP_NCSTN
ANXA1_FPR1
RIMS2_ABCA1
RARRES2_CCRL2
CYR61_ITGB5
CYR61_ITGAM
CTGF_ITGAM
BMP2_ENG
TGFB2_TGFBR1
MIF_TNFRSF14
MIF_TNFRSF10D
LIN7C_ABCA1
MDK_LRP1
MIF_CD74
TNC_ITGB1
TNC_ITGA5
LRP1B_PLAUR
DLL3_NOTCH2
CCL5_CCR1
BMP7_ENG
SERPINE2_LRP1
APP_LRP1
JAM3_ITGB2:ITGAM
OMG_TNFRSF1B
THY1_ITGB2:ITGAM
LGALS3BP_ITGB1
THY1_ITGAX:ITGB2
APP_CD74
PTN_PLXNB2
VCAN_ITGB1
VCAN_TLR2
0
10
20
B
THBS1_CD47
LGALS9_CD44
IL18_IL1RAPL1
IL16_KCND2
HBEGF_CD44
FGL1_EGFR
ADAM17_NOTCH1
SEMA4D_PLXNB1
MFNG_NOTCH2
JAG1_NOTCH1
ANXA1_EGFR
IGF1_IGF1R
HBEGF_CD9
EFNB2_EPHB1
TNF_TNFRSF21
SYTL3_NRXN1
PDGFC_PDGFRA
GNAI2_F2R
AREG_EGFR
WNT5A_FZD3
C1QA_CSPG4
MFNG_NOTCH1
IL16_KCNJ10
TGFB1_EGFR
SIRPA_CD47
PDGFB_PDGFRA
GNAI2_EGFR
HLA−E_KLRC2
TGFB1_ITGB8
HBEGF_CD82
ICAM1_EGFR
HBEGF_EGFR
0
5
10
15
20
Dataset
Darmanis et al. (n = 4)
Neftel et al. (n = 7)
Tirosh et al.; Venteicher et al. (n = 11)
Yu et al. (n = 13)
Yuan et al. (n = 4)

Figure 4

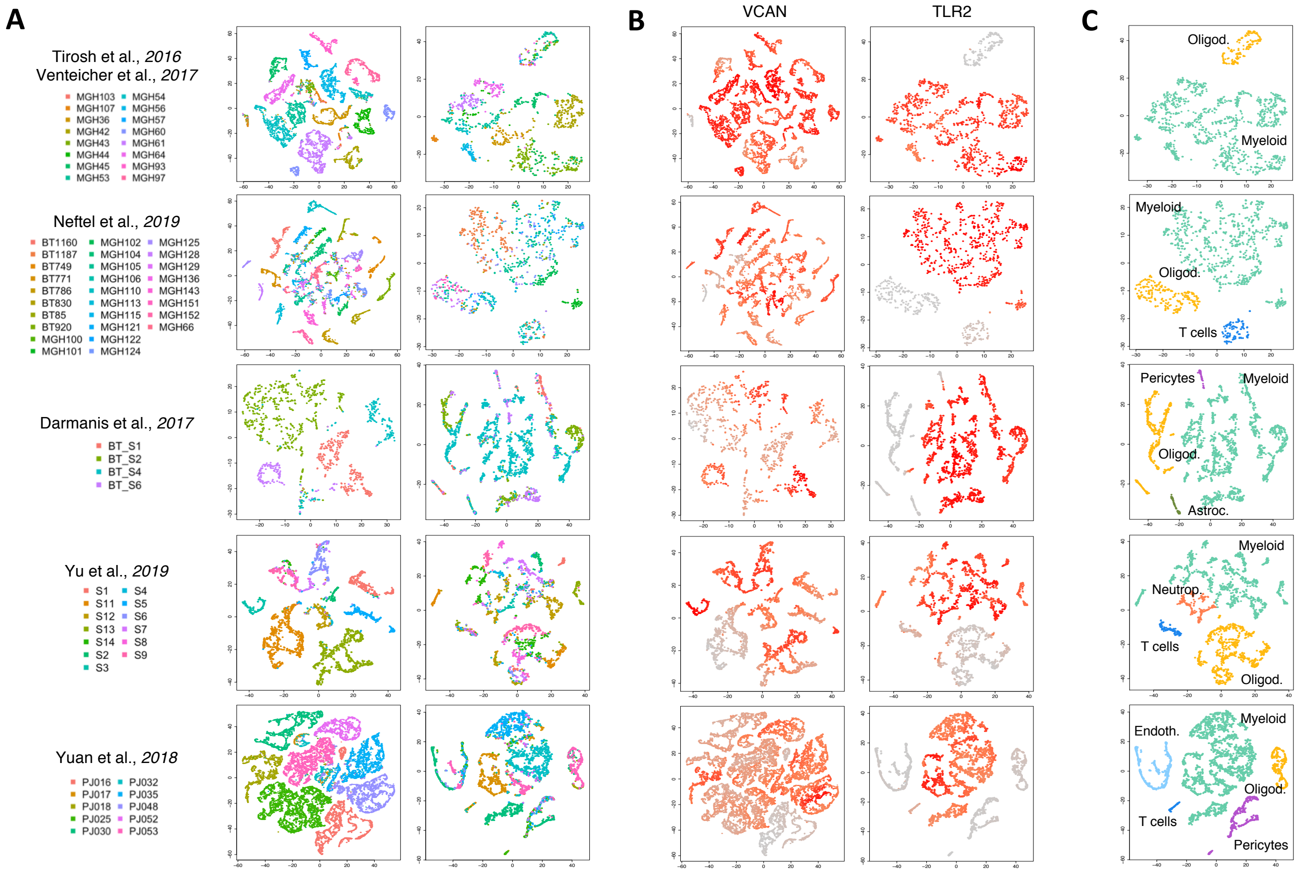
A
Tirosh et al., 2016
Venteicher et al., 2017
MGH103 MGH107 MGH36 MGH42 MGH43 MGH44 MGH45 MGH53 MGH54 MGH56 MGH57 MGH60 MGH61 MGH64 MGH93 MGH97
Neftel et al., 2019
BT1160 BT1187 BT749 BT771 BT786 BT830 BT85 BT920 MGH100 MGH101 MGH102 MGH104 MGH105 MGH106 MGH110 MGH113 MGH115 MGH121 MGH122 MGH124 MGH125 MGH128 MGH129 MGH136 MGH143 MGH151 MGH152 MGH66
Darmanis et al., 2017
BT_S1 BT_S2 BT_S4 BT_S6
Yu et al., 2019
S1 S11 S12 S13 S14 S2 S3 S4 S5 S6 S7 S8 S9
Yuan et al., 2018
PJ016 PJ017 PJ018 PJ025 PJ030 PJ032 PJ035 PJ048 PJ052 PJ053
B
VCAN
TLR2
C
Oligod.
Myeloid
T cells
Pericytes
Astroc.
Neutrop.
Endoth.

Figure S1

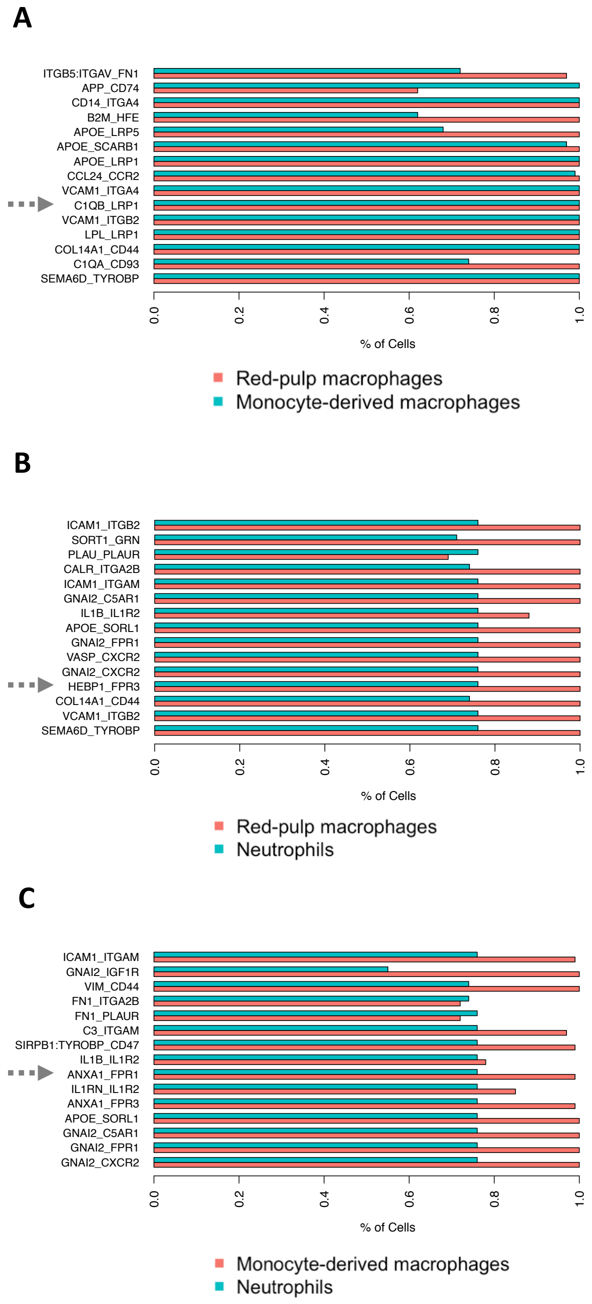
A
ITGB5:ITGAV_FN1
APP_CD74
CD14_ITGA4
B2M_HFE
APOE_LRP5
APOE_SCARB1
APOE_LRP1
CCL24_CCR2
VCAM1_ITGA4
C1QB_LRP1
VCAM1_ITGB2
LPL_LRP1
COL14A1_CD44
C1QA_CD93
SEMA6D_TYROBP
0.0
0.2
0.4
0.6
0.8
1.0
% of Cells
Red-pulp macrophages
Monocyte-derived macrophages
B
ICAM1_ITGB2
SORT1_GRN
PLAU_PLAUR
CALR_ITGA2B
ICAM1_ITGAM
GNAI2_C5AR1
IL1B_IL1R2
APOE_SORL1
GNAI2_FPR1
VASP_CXCR2
GNAI2_CXCR2
HEBP1_FPR3
COL14A1_CD44
VCAM1_ITGB2
SEMA6D_TYROBP
0.0
0.2
0.4
0.6
0.8
1.0
% of Cells
Red-pulp macrophages
Neutrophils
C
ICAM1_ITGAM
GNAI2_IGF1R
VIM_CD44
FN1_ITGA2B
FN1_PLAUR
C3_ITGAM
SIRPB1:TYROBP_CD47
IL1B_IL1R2
ANXA1_FPR1
IL1RN_IL1R2
ANXA1_FPR3
APOE_SORL1
GNAI2_C5AR1
GNAI2_FPR1
GNAI2_CXCR2
0.0
0.2
0.4
0.6
0.8
1.0
% of Cells
Monocyte-derived macrophages
Neutrophils

Figure S2

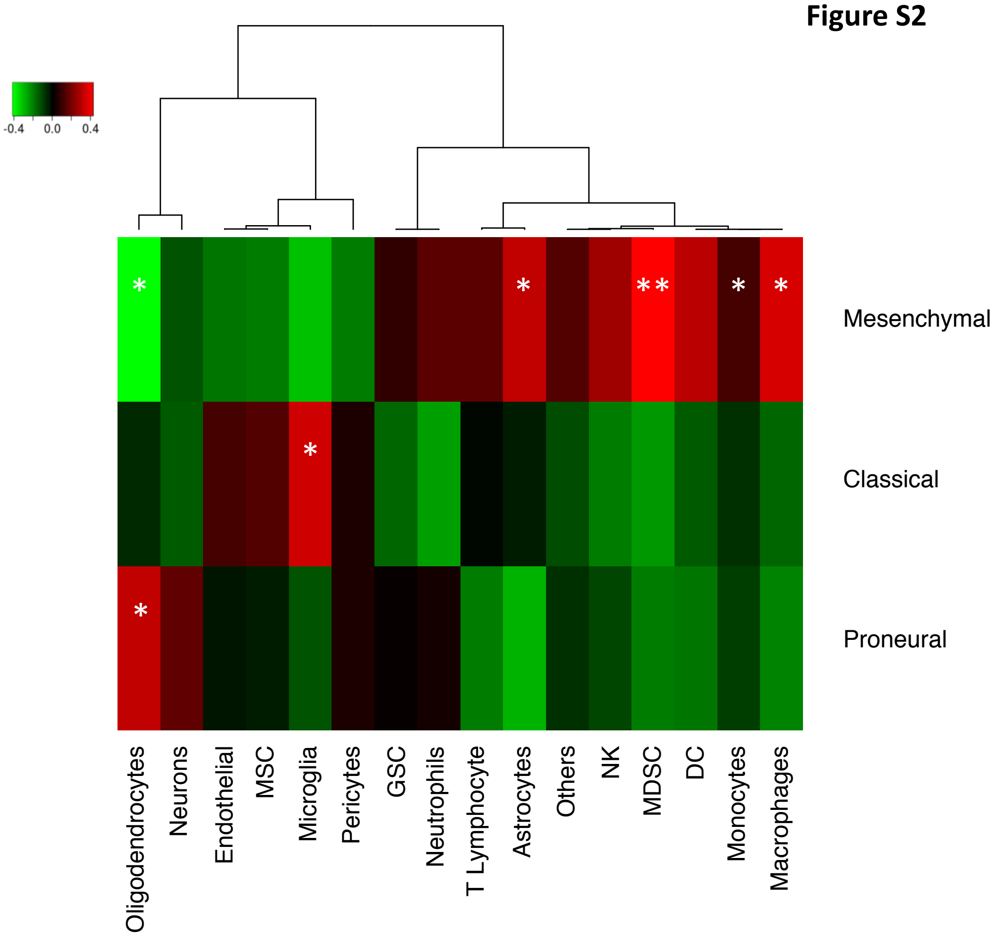

-0.4
0.0
0.4
*
*
*
**
*
*
*
Mesenchymal
Classical
Proneural
Oligodendrocytes
Neurons
Endothelial
MSC
Microglia
Pericytes
GSC
Neutrophils
T Lymphocyte
Astrocytes
Others
NK
MDSC
DC
Monocytes
Macrophages

Figure S3

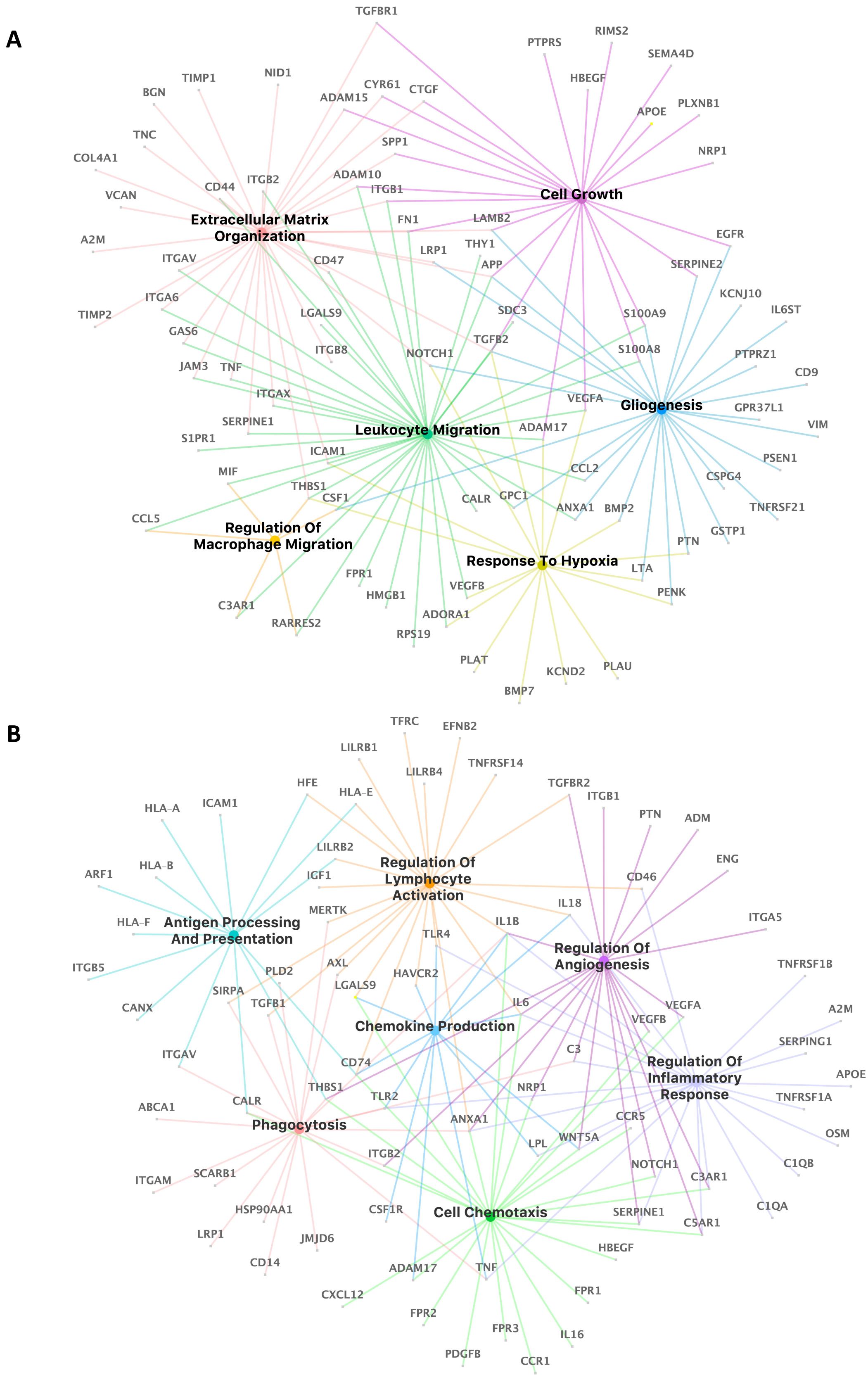
A
TGFBR1
PTPRS
RIMS2
SEMA4D
HBEGF
APOE
PLXNB1
NRP1
BGN
TIMP1
NID1
CYR61
CTGF
ADAM15
TNC
SPP1
COL4A1
VCAN
CD44
ITGB2
ADAM10
ITGB1
Cell Growth
Extracellular Matrix Organization
FN1
LAMB2
EGFR
A2M
LRP1
THY1
ITGAV
CD47
APP
SERPINE2
KCNJ10
ITGA6
IL6ST
TIMP2
LGALS9
SDC3
S100A9
GAS6
TGFB2
S100A8
PTPRZ1
ITGB8
NOTCH1
CD9
JAM3
TNF
ITGAX
VEGFA
Gliogenesis
GPR37L1
VIM
SERPINE1
Leukocyte Migration
ADAM17
S1PR1
ICAM1
PSEN1
MIF
CCL2
THBS1
CSPG4
CSF1
CALR
GPC1
ANXA1
BMP2
TNFRSF21
CCL5
Regulation Of Macrophage Migration
GSTP1
PTN
Response To Hypoxia
LTA
FPR1
VEGFB
PENK
C3AR1
HMGB1
ADORA1
RARRES2
RPS19
PLAT
PLAU
KCND2
BMP7
B
TFRC
EFNB2
LILRB1
TNFRSF14
LILRB4
HFE
TGFBR2
HLA-E
ITGB1
HLA-A
ICAM1
PTN
ADM
LILRB2
ENG
ARF1
HLA-B
IGF1
Regulation Of Lymphocyte Activation
CD46
IL18
MERTK
ITGA5
HLA-F
Antigen Processing And Presentation
IL1B
TLR4
Regulation Of Angiogenesis
ITGB5
PLD2
AXL
HAVCR2
TNFRSF1B
SIRPA
LGALS9
CANX
TGFB1
IL6
VEGFA
A2M
Chemokine Production
VEGFB
SERPING1
ITGAV
CD74
C3
Regulation Of Inflammatory Response
APOE
THBS1
NRP1
TNFRSF1A
ABCA1
CALR
TLR2
CCR5
ANXA1
Phagocytosis
OSM
LPL
WNT5A
ITGB2
NOTCH1
C1QB
ITGAM
SCARB1
C3AR1
C1QA
HSP90AA1
CSF1R
Cell Chemotaxis
SERPINE1
C5AR1
LRP1
JMJD6
HBEGF
CD14
ADAM17
TNF
CXCL12
FPR1
FPR2
FPR3
IL16
PDGFB
CCR1

Figure S4

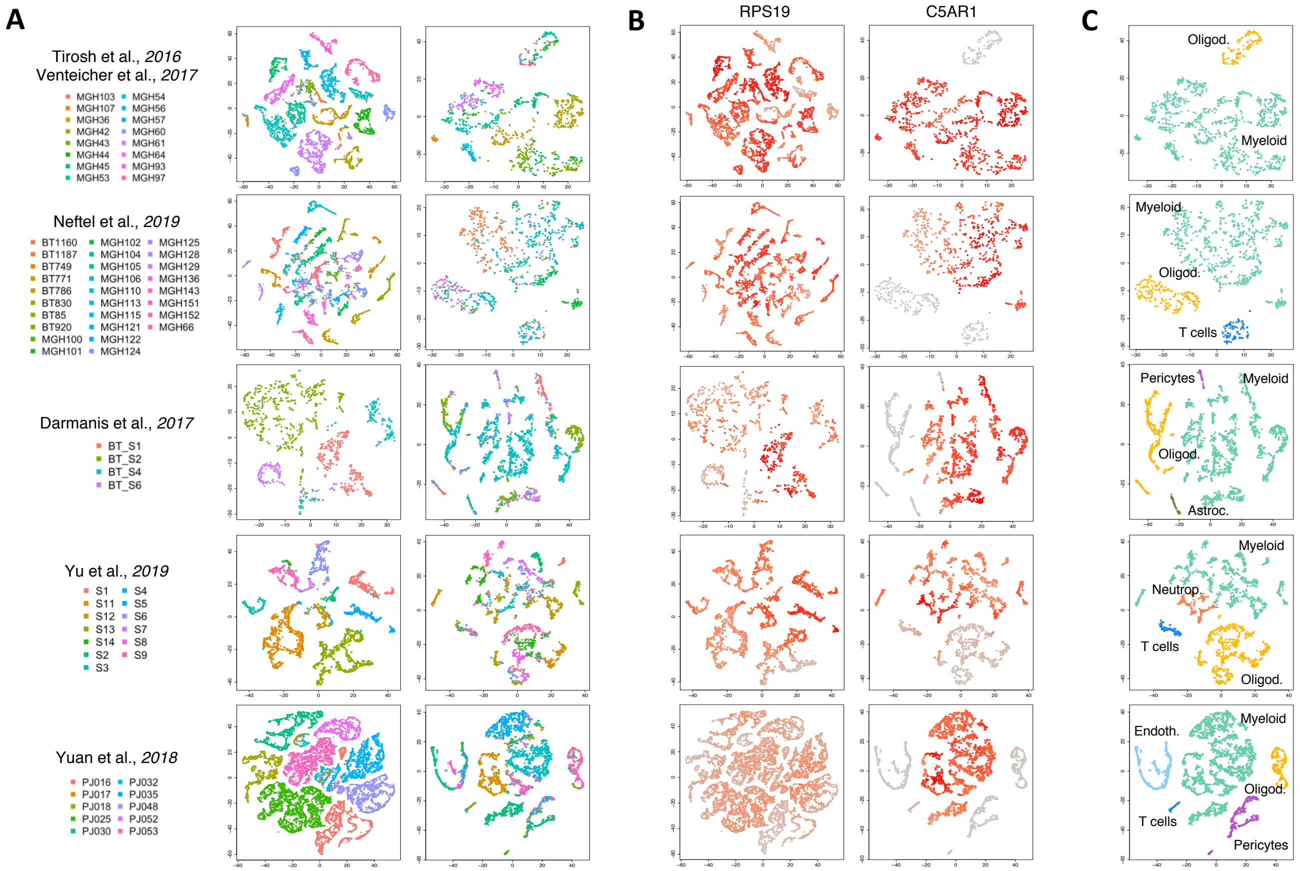
A
Tirosh et al., *2016*
Venteicher et al., *2017*
MGH103 MGH107 MGH36 MGH42 MGH43 MGH44 MGH45 MGH53 MGH54 MGH56 MGH57 MGH60 MGH61 MGH64 MGH93 MGH97
Neftel et al., *2019*
BT1160 BT1187 BT749 BT771 BT786 BT830 BT85 BT920 MGH100 MGH101 MGH102 MGH104 MGH105 MGH106 MGH110 MGH113 MGH115 MGH121 MGH122 MGH124 MGH125 MGH128 MGH129 MGH136 MGH143 MGH151 MGH152 MGH66
Darmanis et al., *2017*
BT_S1 BT_S2 BT_S4 BT_S6
Yu et al., *2019*
S1 S11 S12 S13 S14 S2 S3 S4 S5 S6 S7 S8 S9
Yuan et al., *2018*
PJ016 PJ017 PJ018 PJ025 PJ030 PJ032 PJ035 PJ048 PJ052 PJ053
B
RPS19
C5AR1
C
Oligod.
Myeloid
Myeloid
Oligod.
T cells
Pericytes
Myeloid
Oligod.
Astroc.
Myeloid
Neutrop.
T cells
Oligod.
Endoth.
Myeloid
Oligod.
T cells
Pericytes

Figure S5

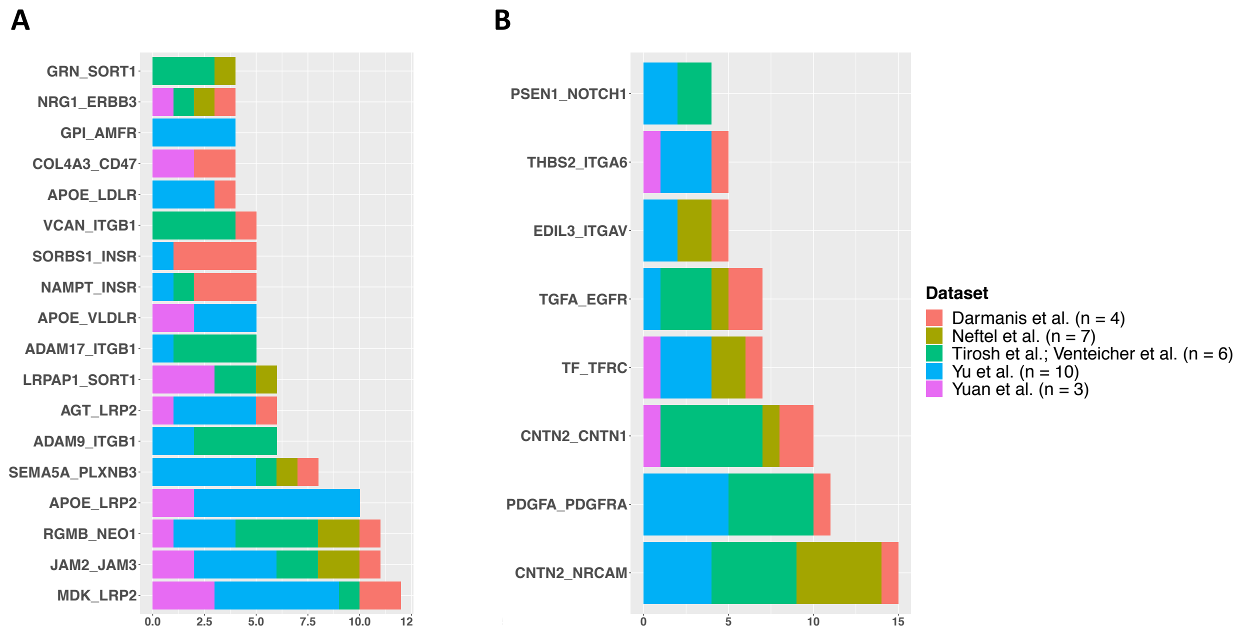

A
GRN_SORT1
NRG1_ERBB3
GPI_AMFR
COL4A3_CD47
APOE_LDLR
VCAN_ITGB1
SORBS1_INSR
NAMPT_INSR
APOE_VLDLR
ADAM17_ITGB1
LRPAP1_SORT1
AGT_LRP2
ADAM9_ITGB1
SEMA5A_PLXNB3
APOE_LRP2
RGMB_NEO1
JAM2_JAM3
MDK_LRP2
0.0
2.5
5.0
7.5
10.0
12
B
PSEN1_NOTCH1
THBS2_ITGA6
EDIL3_ITGAV
TGFA_EGFR
TF_TFRC
CNTN2_CNTN1
PDGFA_PDGFRA
CNTN2_NRCAM
0
5
10
15
Dataset
Darmanis et al. (n = 4)
Neftel et al. (n = 7)
Tirosh et al.; Venteicher et al. (n = 6)
Yu et al. (n = 10)
Yuan et al. (n = 3)

Figure S6

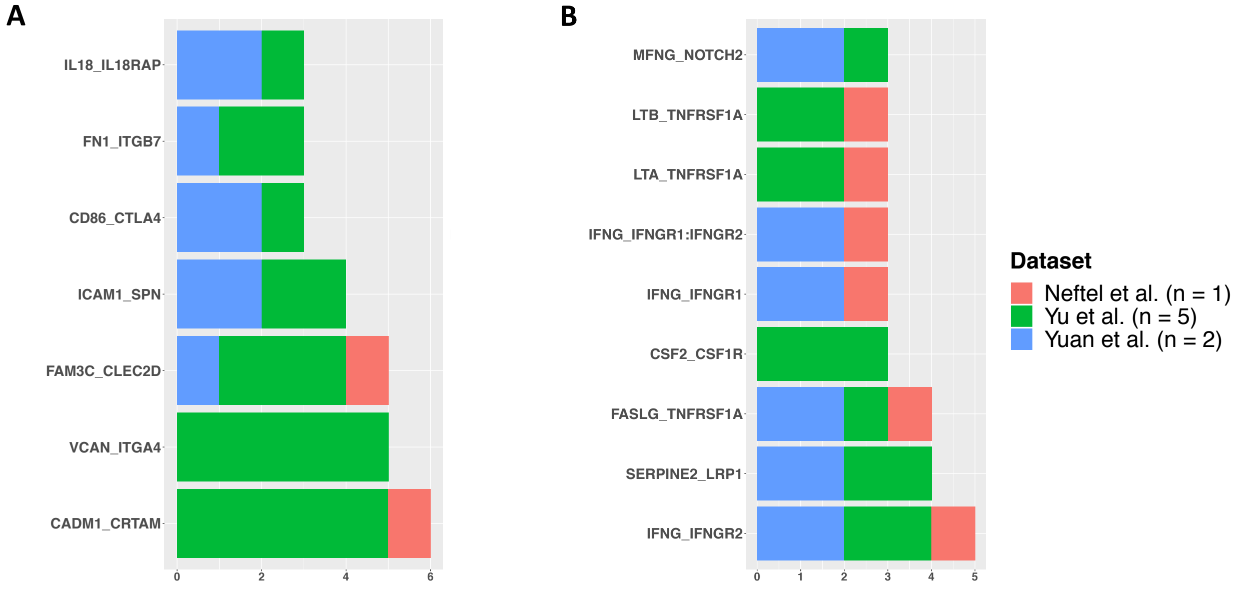

A
IL18_IL18RAP
FN1_ITGB7
CD86_CTLA4
ICAM1_SPN
FAM3C_CLEC2D
VCAN_ITGA4
CADM1_CRTAM
0
2
4
6
B
MFNG_NOTCH2
LTB_TNFRSF1A
LTA_TNFRSF1A
IFNG_IFNGR1:IFNGR2
IFNG_IFNGR1
CSF2_CSF1R
FASLG_TNFRSF1A
SERPINE2_LRP1
IFNG_IFNGR2
0
1
2
3
4
5
Dataset
Neftel et al. (n = 1)
Yu et al. (n = 5)
Yuan et al. (n = 2)

Figure S7

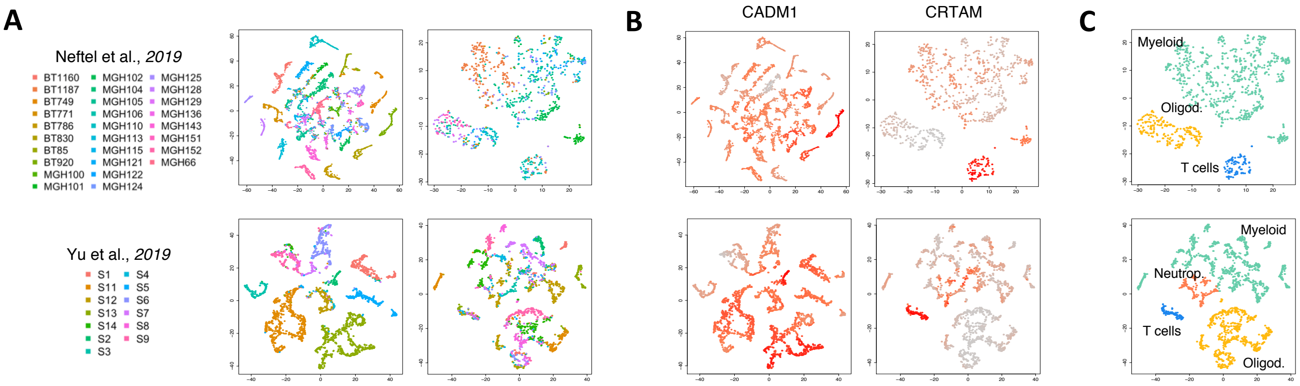
A
Neftel et al., 2019
BT1160
BT1187
BT749
BT771
BT786
BT830
BT85
BT920
MGH100
MGH101
MGH102
MGH104
MGH105
MGH106
MGH110
MGH113
MGH115
MGH121
MGH122
MGH124
MGH125
MGH128
MGH129
MGH136
MGH143
MGH151
MGH152
MGH66
Yu et al., 2019
S1
S11
S12
S13
S14
S2
S3
S4
S5
S6
S7
S8
S9
B
CADM1
CRTAM
C
Myeloid
Oligod.
T cells
Myeloid
Neutrop.
T cells
Oligod.

Figure S8

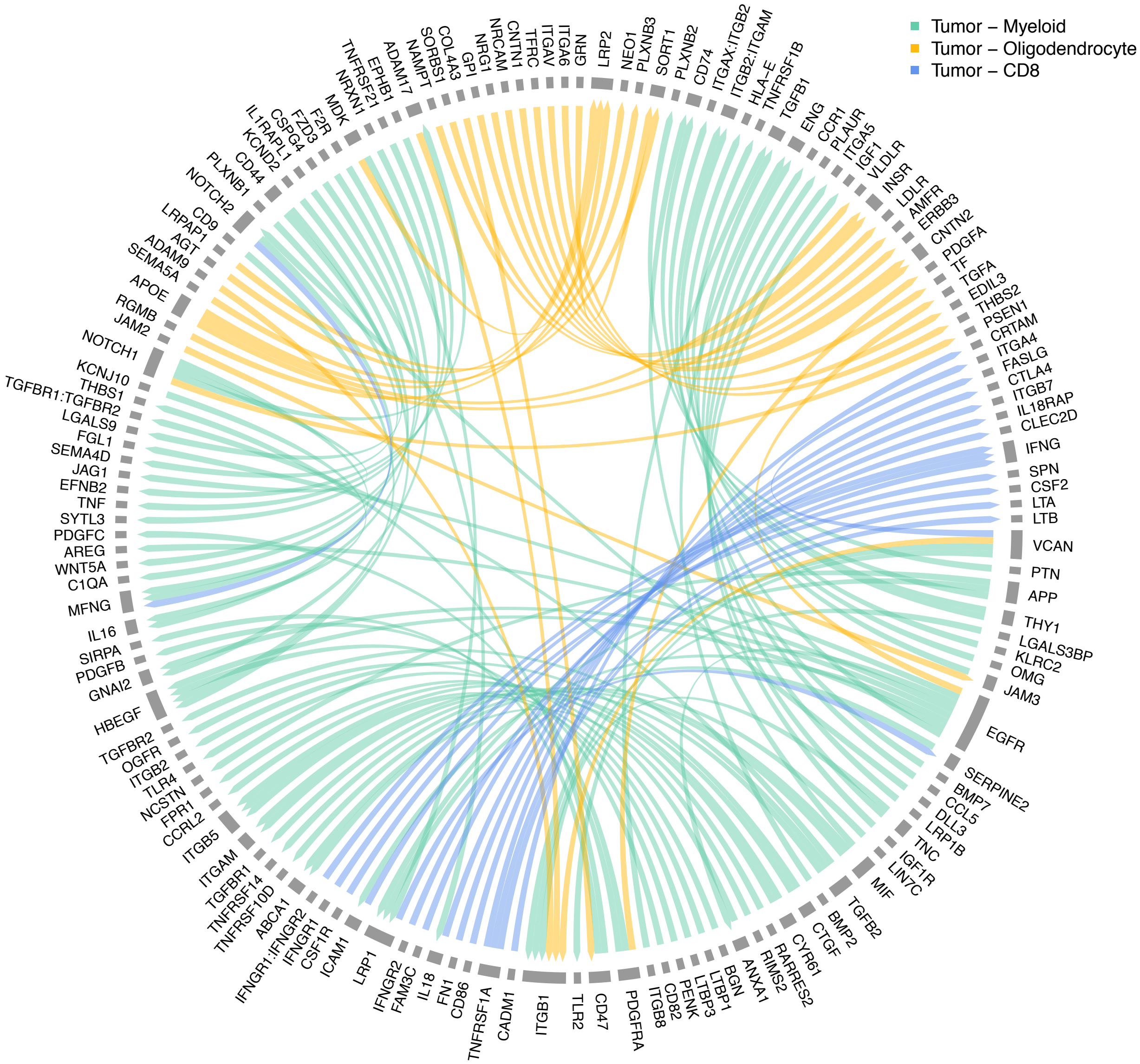
Tumor – Myeloid
Tumor – Oligodendrocyte
Tumor – CD8
LRP2
NEO1
PLXNB3
SORT1
PLXNB2
CD74
ITGAX:ITGB2
ITGB2:ITGAM
HLA-E
TNFRSF1B
TGFB1
ENG
CCR1
PLAUR
ITGA5
IGF1
VLDLR
INSR
LDLR
AMFR
ERBB3
CNTN2
PDGFA
TF
TGFA
EDIL3
THBS2
PSEN1
CRTAM
ITGA4
FASLG
CTLA4
ITGB7
IL18RAP
CLEC2D
IFNG
SPN
CSF2
LTA
LTB
VCAN
PTN
APP
THY1
LGALS3BP
KLRC2
OMG
JAM3
EGFR
SERPINE2
BMP7
CCL5
DLL3
LRP1B
TNC
IGF1R
LIN7C
MIF
TGFB2
BMP2
CTGF
CYR61
RARRES2
RIMS2
ANXA1
BGN
LTBP1
LTBP3
PENK
CD82
ITGB8
PDGFRA
CD47
TLR2
ITGB1
CADM1
TNFRSF1A
CD86
FN1
IL18
FAM3C
IFNGR2
LRP1
ICAM1
CSF1R
IFNGR1
IFNGR1:IFNGR2
ABCA1
TNFRSF10D
TNFRSF14
TGFBR1
ITGAM
ITGB5
CCRL2
FPR1
NCSTN
TLR4
ITGB2
OGFR
TGFBR2
HBEGF
GNAI2
PDGFB
SIRPA
IL16
MFNG
C1QA
WNT5A
AREG
PDGFC
SYTL3
TNF
EFNB2
JAG1
SEMA4D
FGL1
LGALS9
TGFBR1:TGFBR2
THBS1
KCNJ10
NOTCH1
JAM2
RGMB
APOE
SEMA5A
ADAM9
AGT
LRPAP1
CD9
NOTCH2
PLXNB1
CD44
KCND2
IL1RAPL1
CSPG4
FZD3
F2R
MDK
NRXN1
TNFRSF21
EPHB1
ADAM17
NAMPT
SORBS1
COL4A3
GPI
NRG1
NRCAM
CNTN1
TFRC
ITGAV
ITGA6
GRN

Figure 2

Click here to access/download;Figure;Figure2.pdf

Figure 2

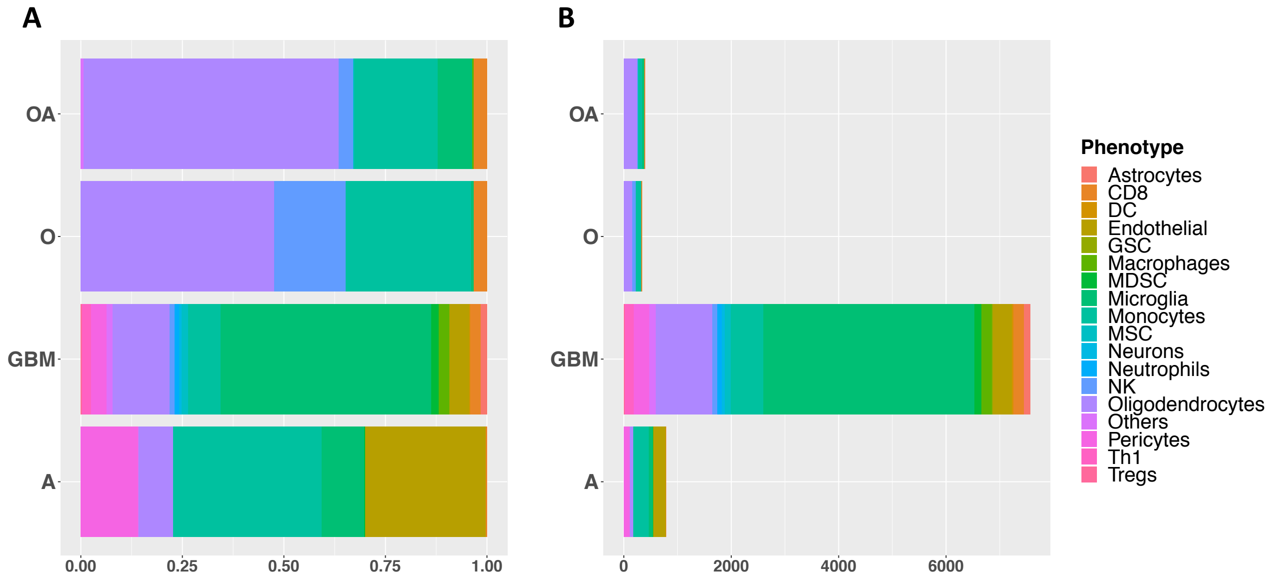

Figure 3

Click here to access/download;Figure;Figure3.pdf

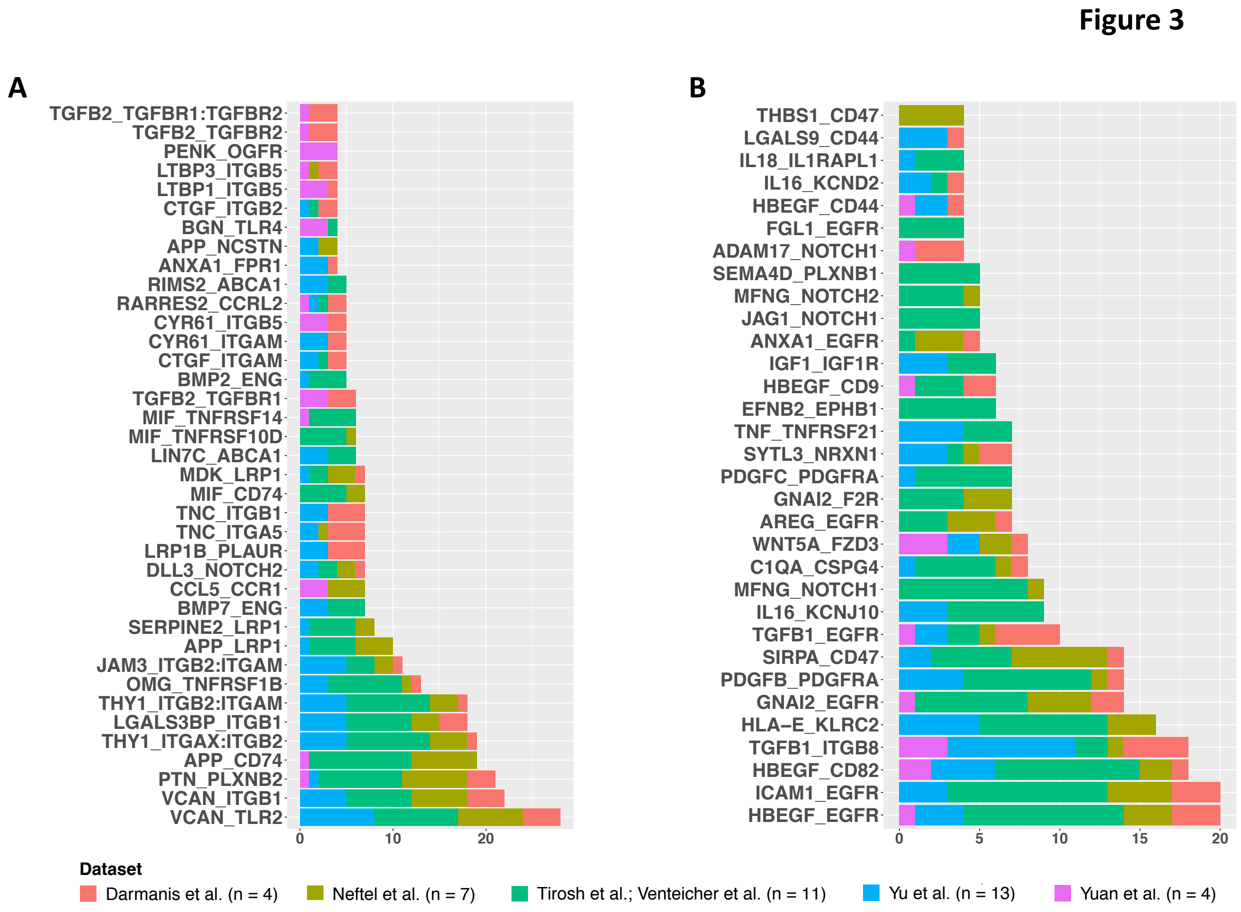

Figure 4

Click here to access/download;Figure;Figure4.pdf

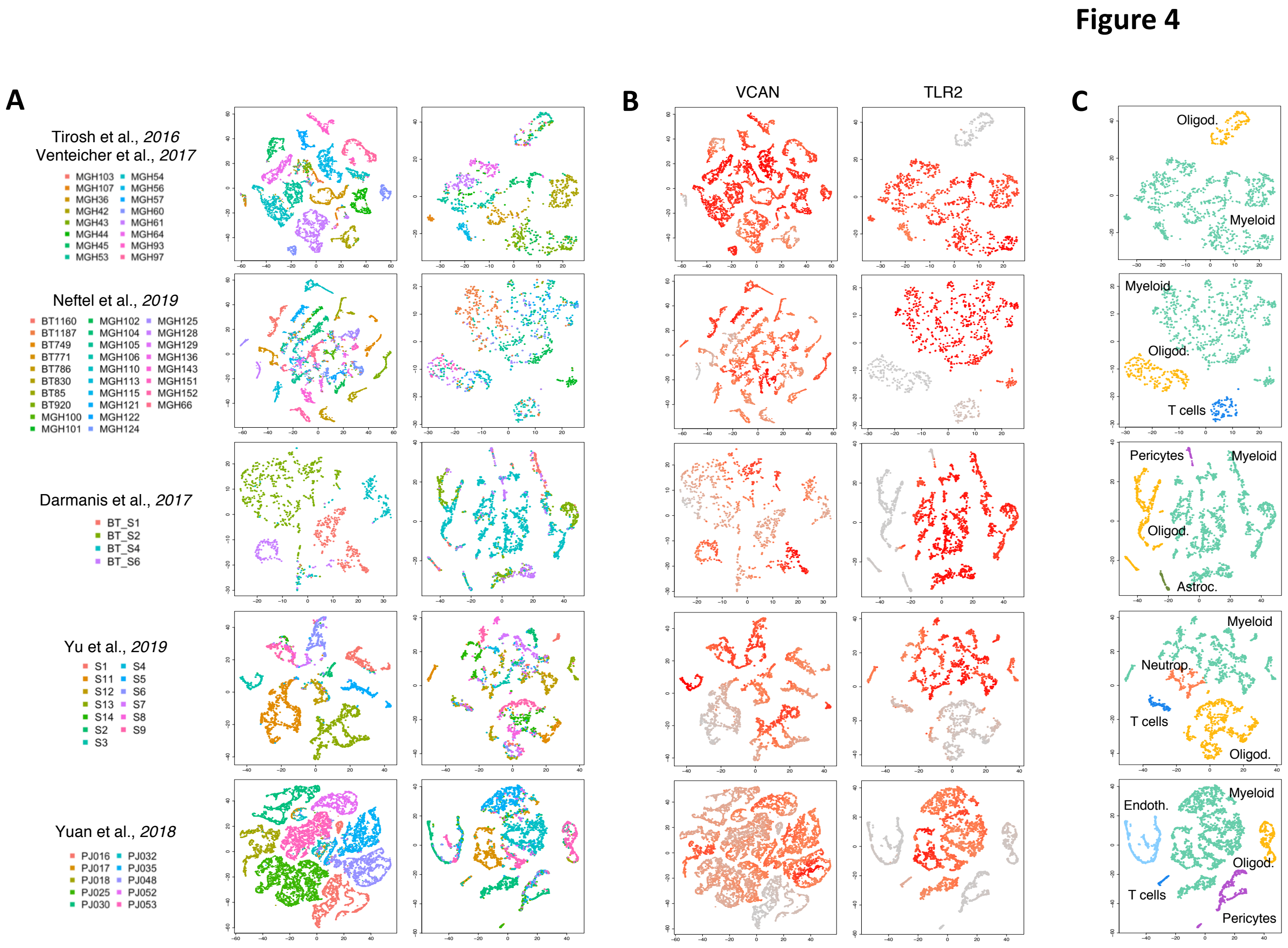

Figure S1

Click here to access/download;Figure;FigureS1.pdf

Figure S1

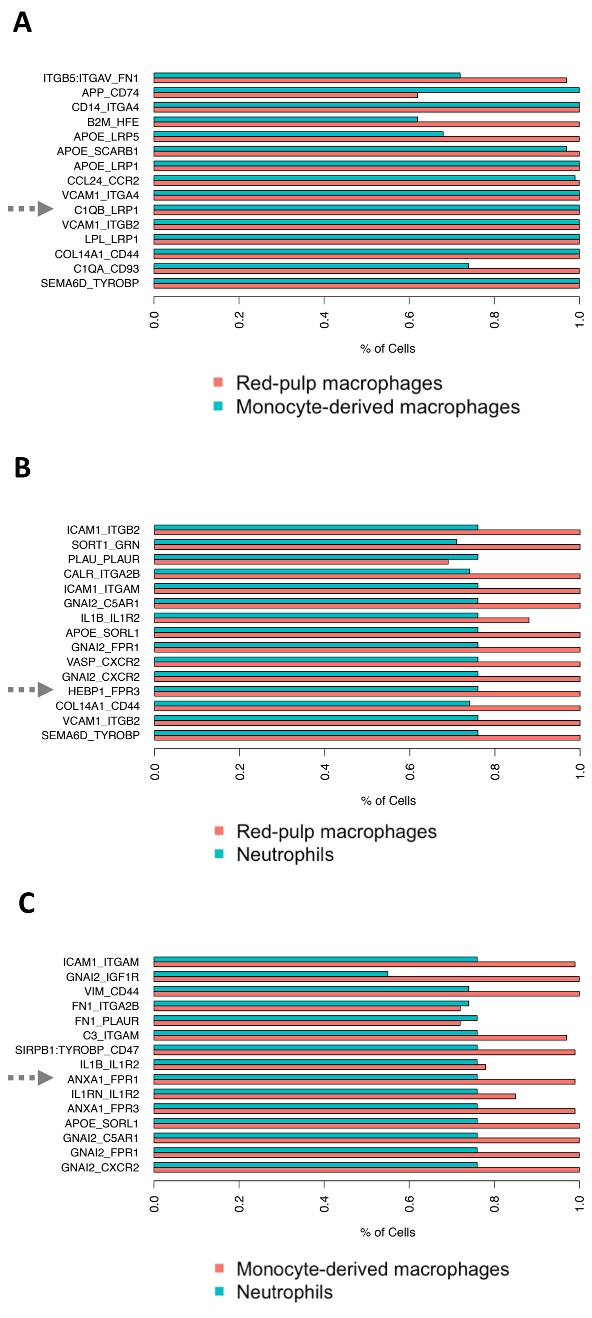

Figure S2

Click here to access/download;Figure;FigureS2.pdf

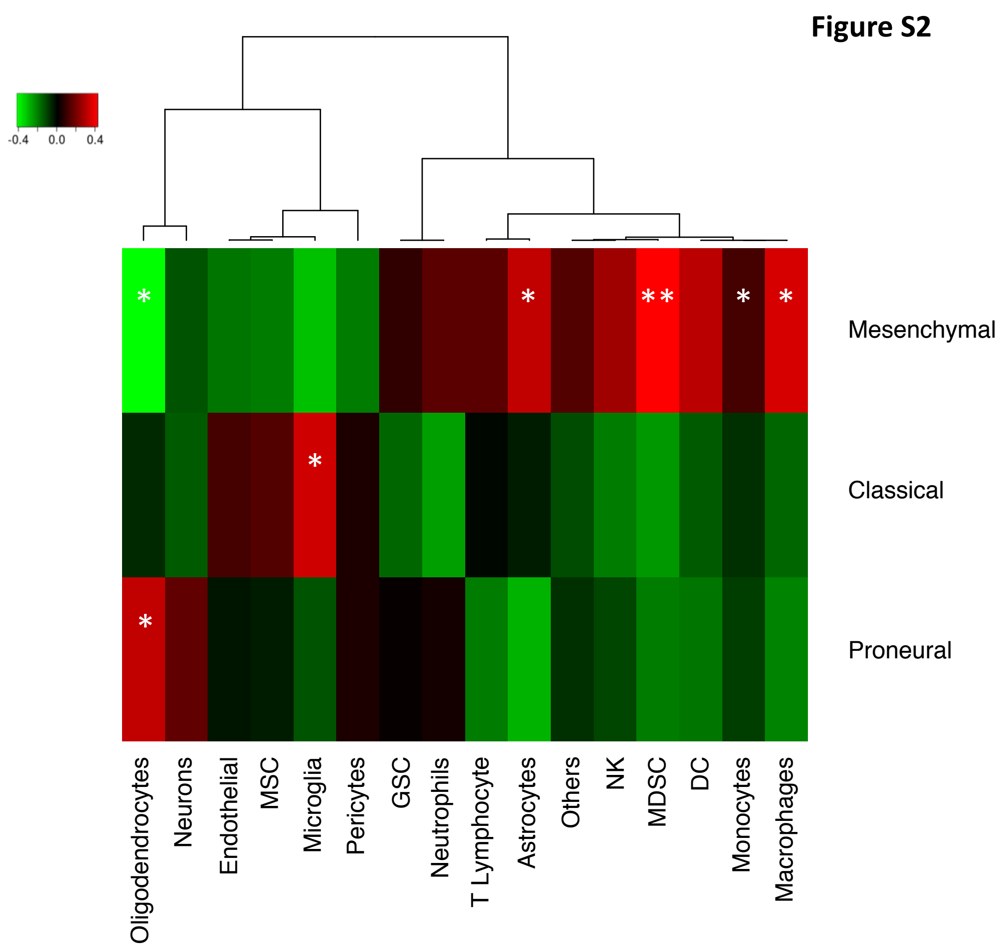

Click here to access/download;Figure;FigureS3.pdf

Figure S3

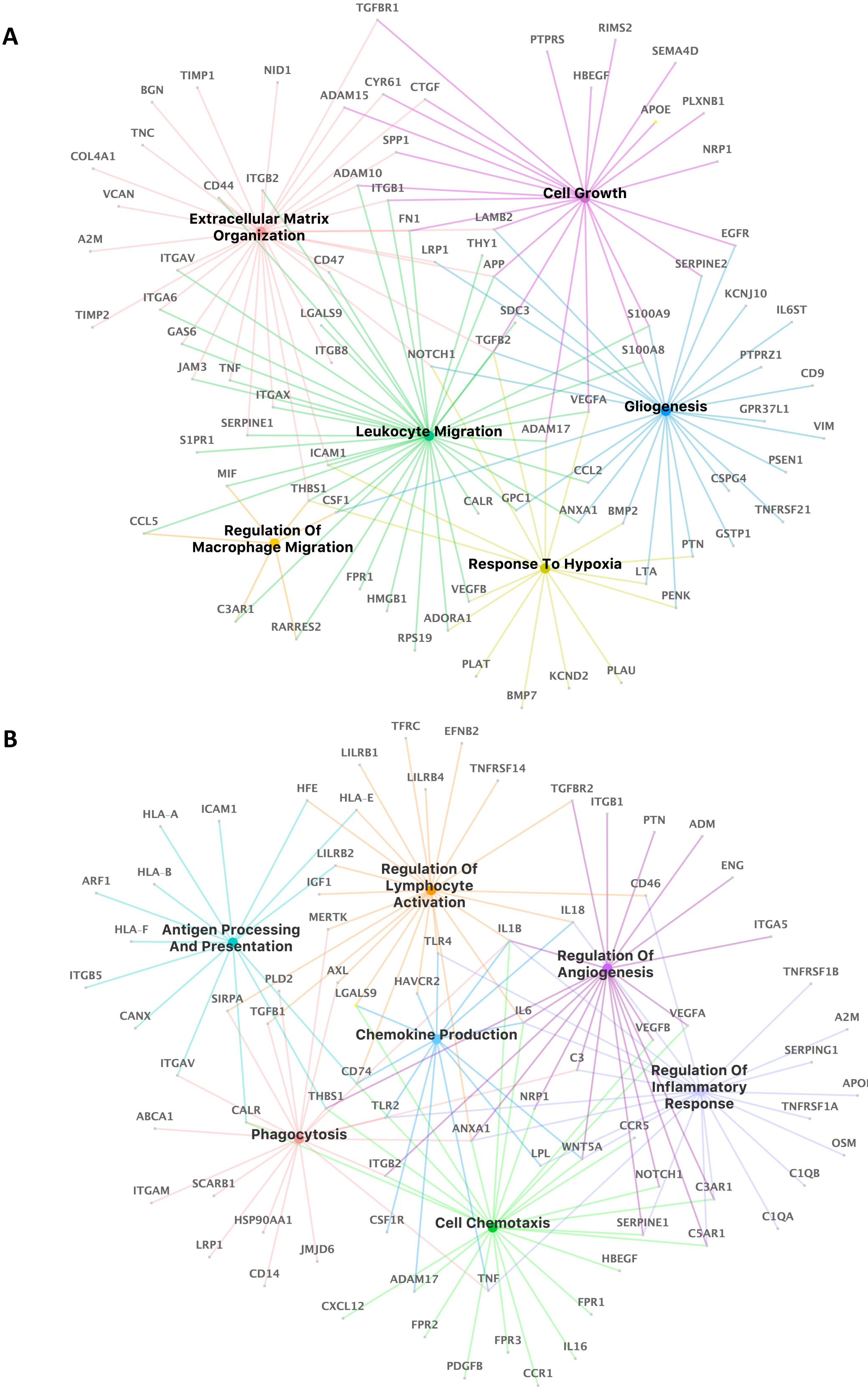

Figure S4

Click here to access/download;Figure;FigureS4.pdf

Figure S4

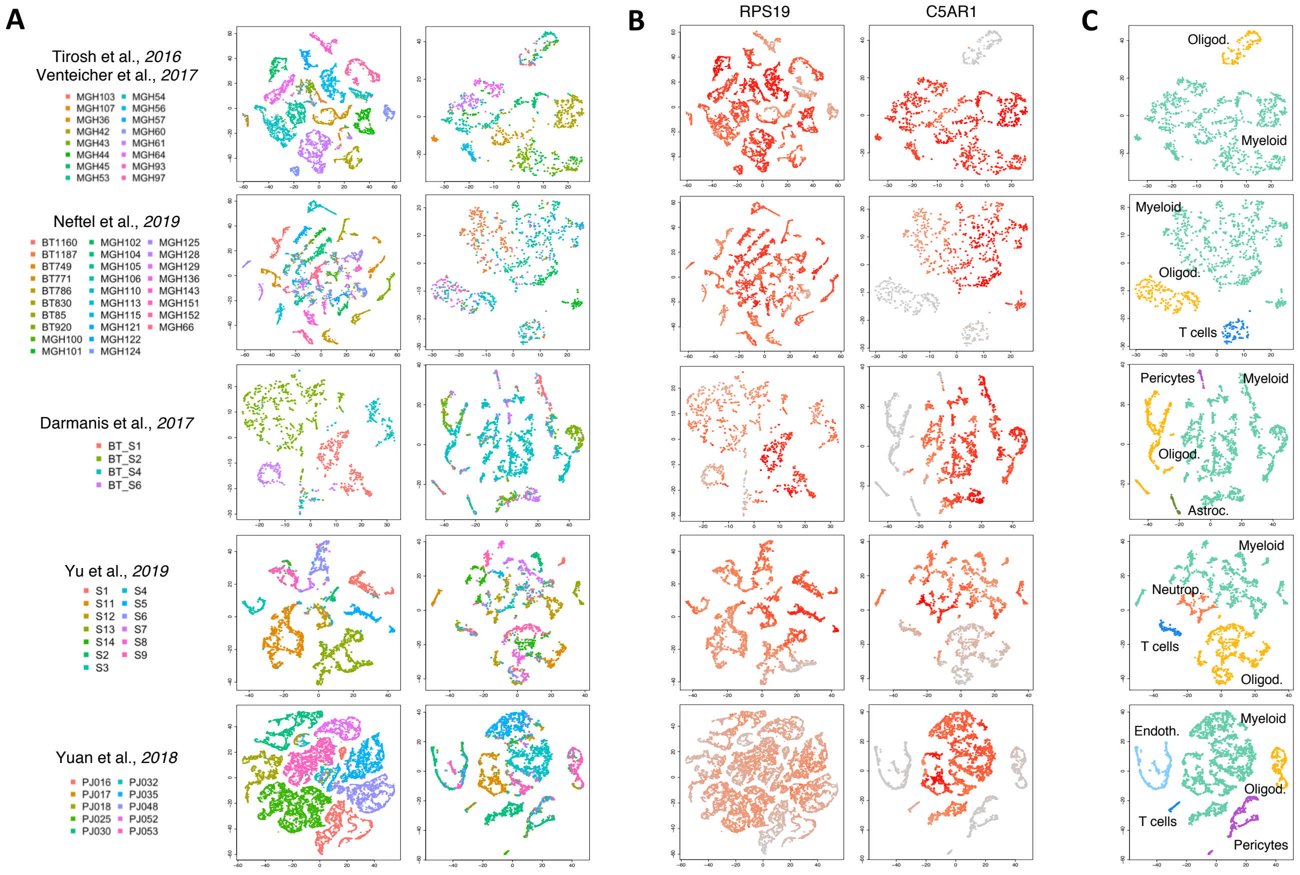

Figure S5

Click here to access/download;Figure;FigureS5.pdf

Figure S5

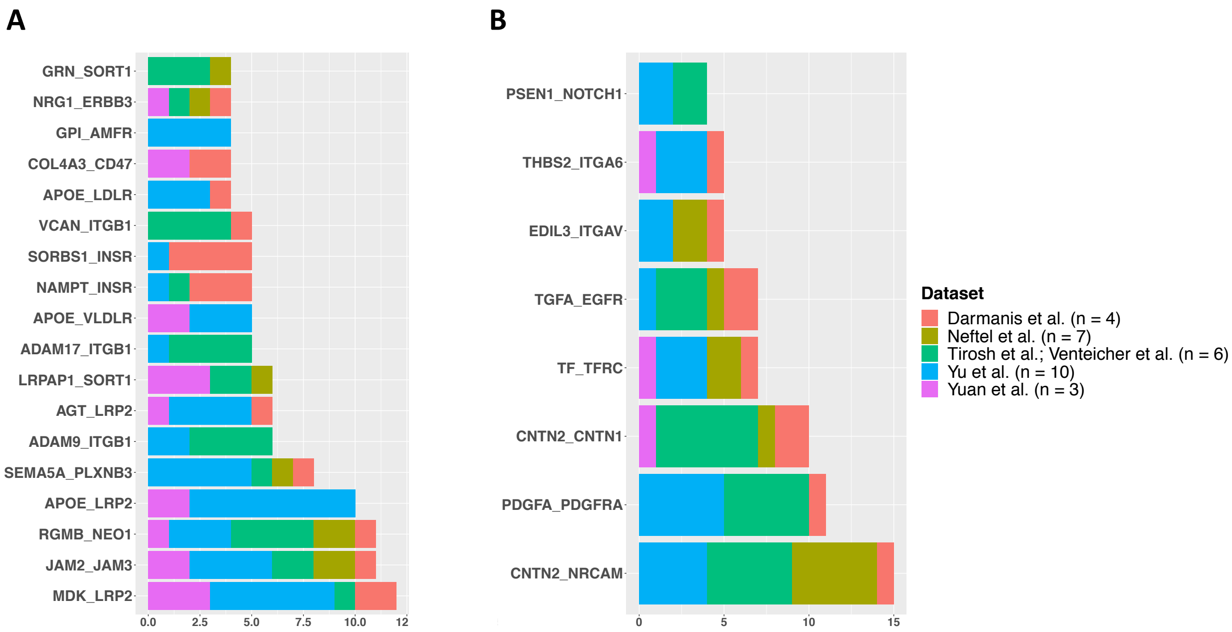

Figure S6

Click here to access/download;Figure;FigureS6.pdf

Figure S6

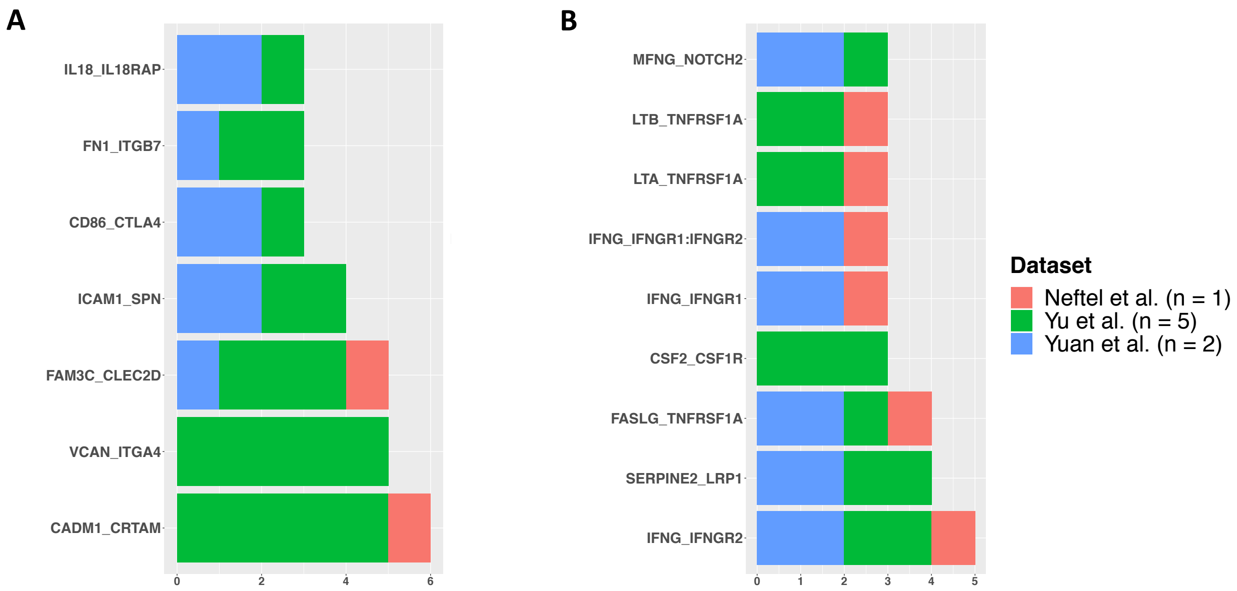

Figure S7

Click here to access/download;Figure;FigureS7.pdf

Figure S7

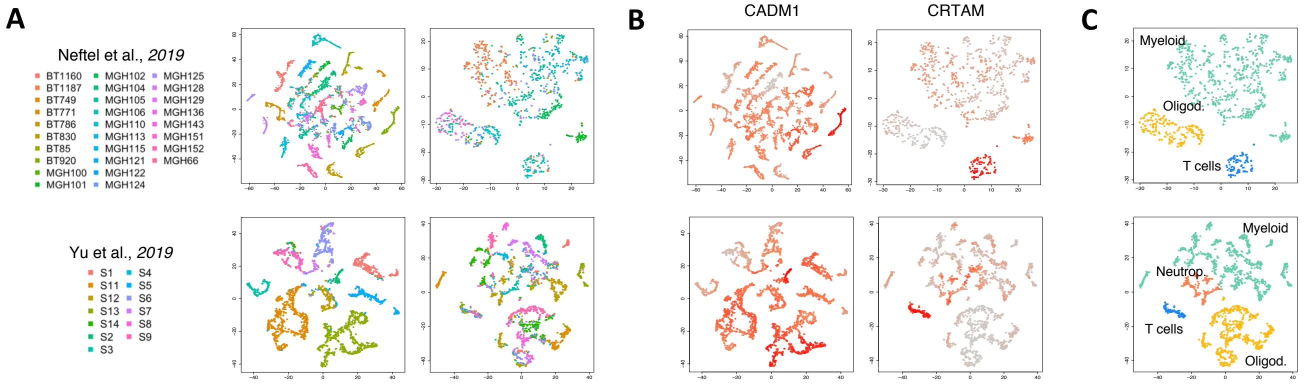

Figure S8

Click here to access/download;Figure;FigureS8.pdf

Figure S8

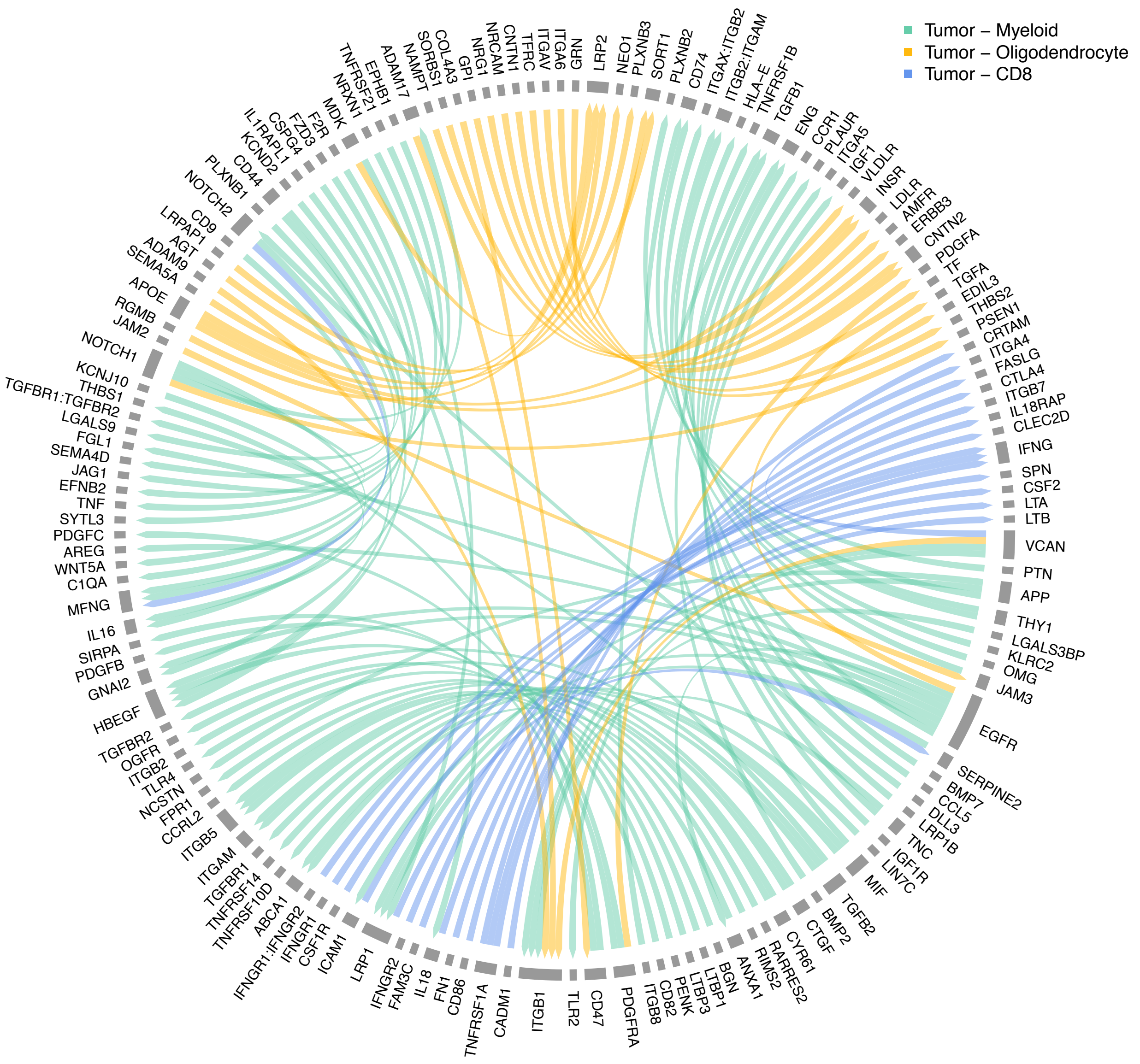

Table S1

Click here to access/download
Supplementary Material
Table S1_interaction.xlsx

Table S2

Click here to access/download
Supplementary Material
Table S2_signatures.xlsx

Table S3

Click here to access/download
Supplementary Material
Table S3_NonTumorCellClassification.xlsx

Table S4

Click here to access/download
Supplementary Material
Table S4_TumorMyeloidInteractions.xlsx

Table S5

Click here to access/download
Supplementary Material
Table S5_GOEnrichmentsOfL-

Table S6

Click here to access/download
Supplementary Material
Table S6_Tumor-Oligodendrocyte interactions.xlsx

Table S7

Click here to access/download
Supplementary Material

Table S7_GO Enrichments of

Table S8

Click here to access/download
Supplementary Material
Table S8_Tumor-CD8 interactions.xlsx

Table S9

Click here to access/download
Supplementary Material
Table S9_myeloidCD8interaction.xlsx

Cover Letter Click here to access/download;Personal Cover;GigaScience Cover Letter.pdf

DIETI UNIVERSITA' DEGLI STUDI DI NAPOLI FEDERICO II
DIPARTIMENTO DI INGEGNERIA ELETTRICA E DELLE TECNOLOGIE DELL'INFORMAZIONE

Dear Editors,
I am happy to submit the paper “A MAP of tumor-host interactions in glioma at single cell resolution” to *GigaScience*. Gliomas account for about 80 percent of all malignant brain tumors. Indeed we have previously characterized the recurrent molecular features of the main subtypes of glioma using bulk gene expression, exome sequencing, methylation microarrays and genotyping microarrays (1). Although gliomas are typically “cold” tumors characterized by an immuno-repressive microenvironment enriched of M2 macrophages, we have shown that a small subset of glioma present high-quality neoantigens and lymphocyte infiltration conferring a relatively better prognosis (2). The presence of this particular phenotype was also recently confirmed by our group in the context of syndromic glioma (3). In this paper, we tried to answer an important open question in the biology of glioma. Which are the main drivers that govern the interaction between the cancer cells and their microenvironment? To address this problem, we collected the largest collection of glioma single-cell rna-seq integrating six different datasets from several groups (45,550 tumor cells and 11,510 non-tumor cells from 71 glioma patients) and developed a novel computational approach, `scTHI`, an algorithm and tool to discover the significantly activated ligand-receptor pairs that modulate the tumor-microenvironment signaling. We report an accurate and complete map of the main ligand-receptor interactions focusing on the cross-talk between mesenchymal GBM tumor cells and myeloid cells, proneural GBM tumor cells and oligodendrocytes, and interactions involving T-cells. We described for the first time some interactions between tumor and myeloid cells that could therapeutically be translated. We also believe that the tool we have developed with the accompanying signatures and curated list of ligand-receptor pairs is an important resource for the scientific community that can be used is several other domains. The assembled cohort results in the most comprehensive dataset to study heterogeneity and the characteristic of the microenvironment in Glioma. Therefore we believe that the manuscript fits the mission of GigaScience and is general enough to be of interest to its readers.

Naples January 16th 2020

Michele Ceccarelli

Michele Ceccarelli

Michele Ceccarelli
Professor of Computer Science
Department of Electrical Engineering and Information Technology (DIETI)
University of Naples “Federico II”

DIPARTIMENTO DI INGEGNERIA ELETTRICA E DELLE TECNOLOGIE DELL'INFORMAZIONE
Università degli Studi di Napoli Federico II
Via Claudio 21, 80125 Naples, Italy

Bibliography

1. Ceccarelli,M., Barthel,F.P., Malta,T.M., Sabedot,T.S., Salama,S.R., Murray,B.A., Morozova,O., Newton,Y., Radenbaugh,A., Pagnotta,S.M., *et al.* (2016) Molecular profiling reveals biologically discrete subsets and pathways of progression in diffuse glioma. *Cell*, **164**, 550–563.

2. Zhang,J., Caruso,F.P., Sa,J.K., Justesen,S., Nam,D.-H., Sims,P., Ceccarelli,M., Lasorella,A. and Iavarone,A. (2019) The combination of neoantigen quality and T lymphocyte infiltrates identifies glioblastomas with the longest survival. *Commun. Biol.*, **2**, 135.

3. D'Angelo,F., Ceccarelli,M., Tala, Garofano,L., Zhang,J., Frattini,V., Caruso,F.P., Lewis,G., Alfaro,K.D., Bauchet,L., *et al.* (2019) The molecular landscape of glioma in patients with Neurofibromatosis 1. *Nat. Med.*, **25**, 176–187.
