# Supplementary figures and images for: A map of tumor–host interactions in glioma at single-cell resolution
Source: Gigascience. 2020 Oct 14;9(10):giaa109. doi: 10.1093/gigascience/giaa109 (PMC7645027; doi:10.1093/gigascience/giaa109)

### Figure S10

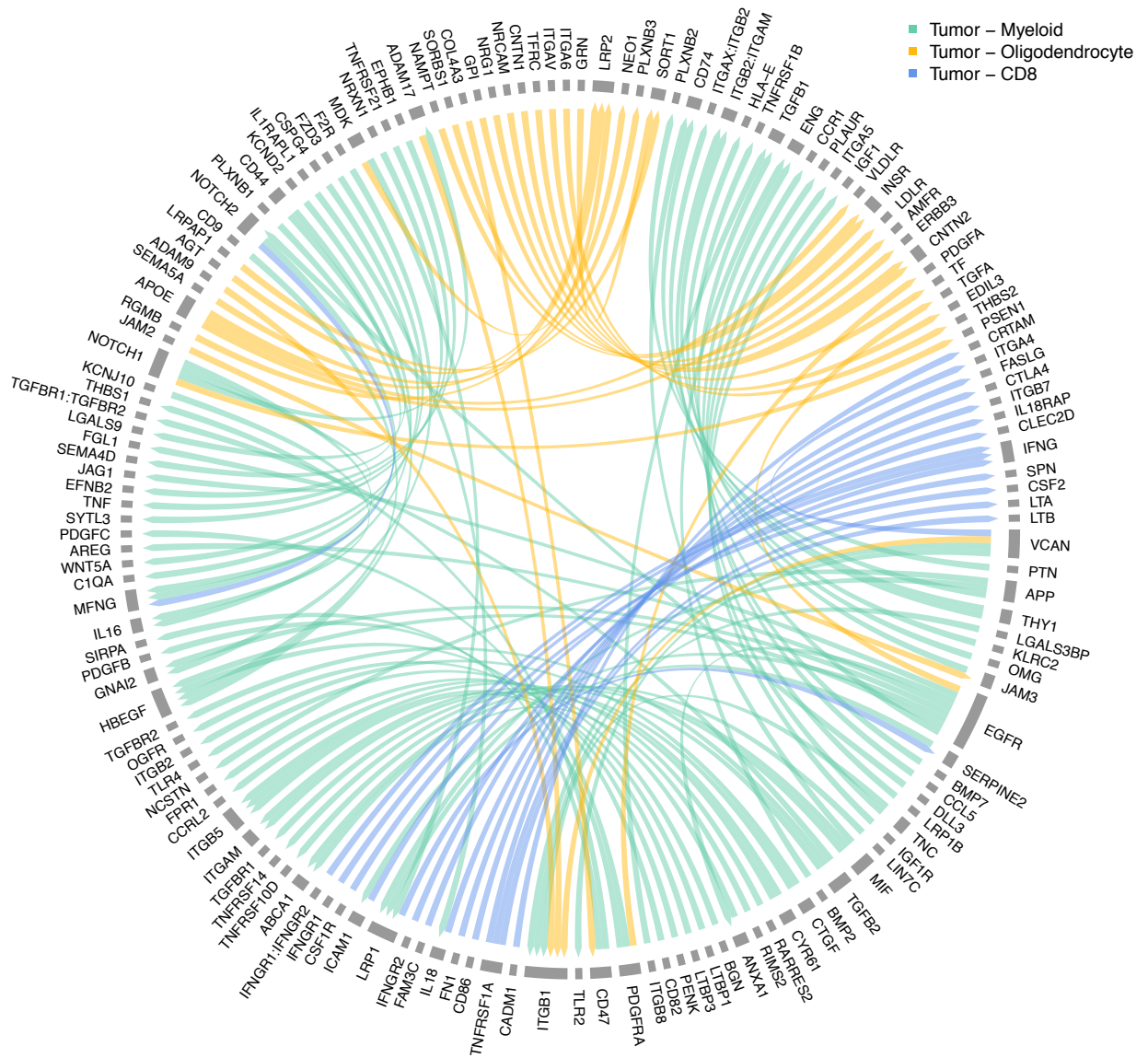

Supplement: giaa109_Supplemental_Figures_and_Tables [file giaa109_supplemental_figures_and_tables.zip › Figure_S10_R1.pdf]

Figure S1

A

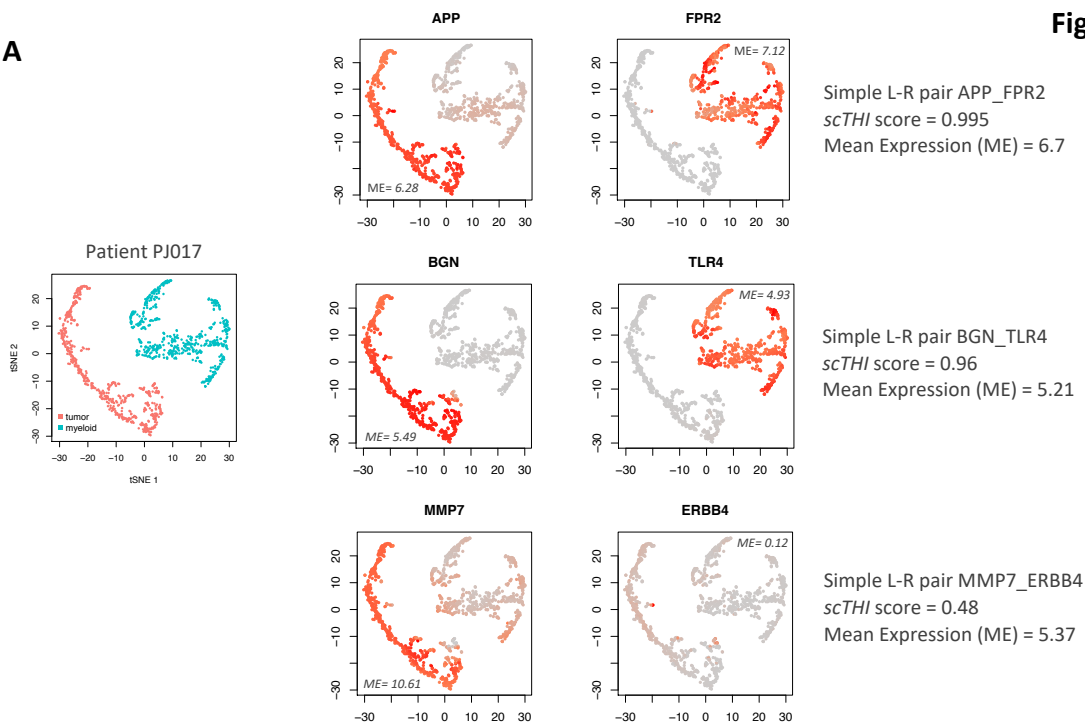

B

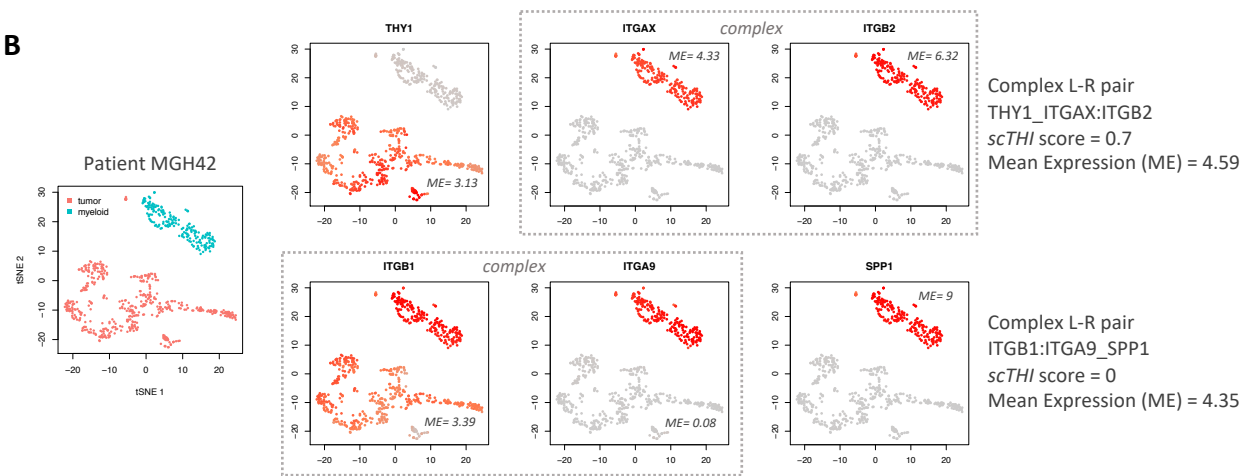

C

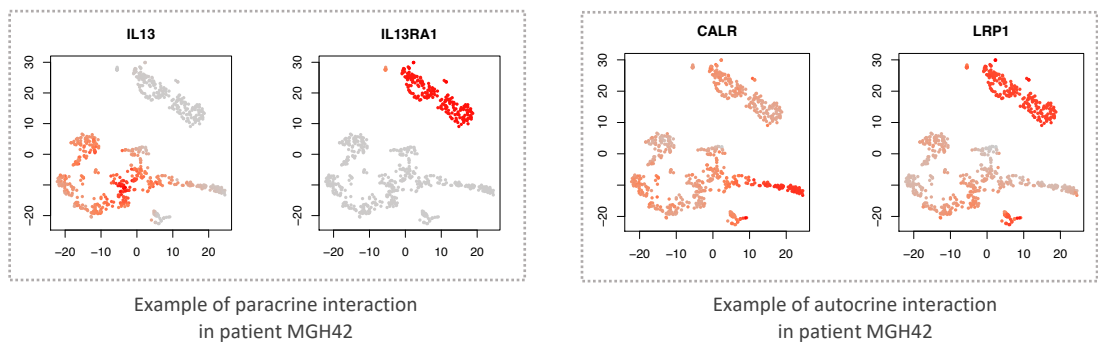

Supplement: giaa109_Supplemental_Figures_and_Tables [file giaa109_supplemental_figures_and_tables.zip › Figure_S1_R1.pdf]

Figure S2

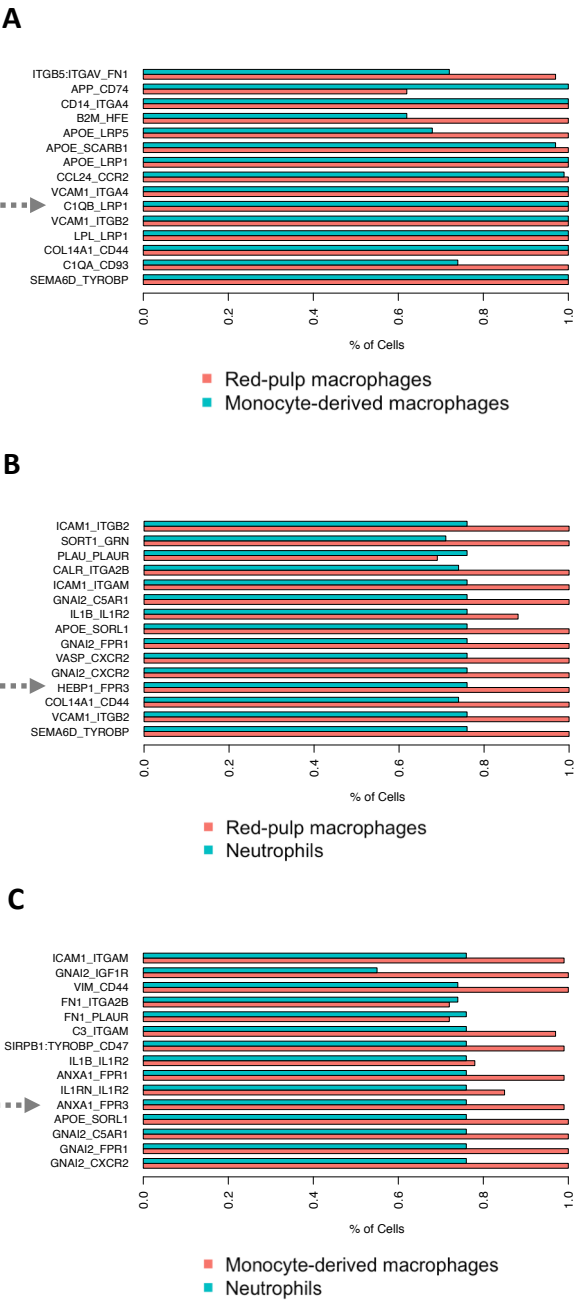

Supplement: giaa109_Supplemental_Figures_and_Tables [file giaa109_supplemental_figures_and_tables.zip › Figure_S2_R1.pdf]

Figure S3

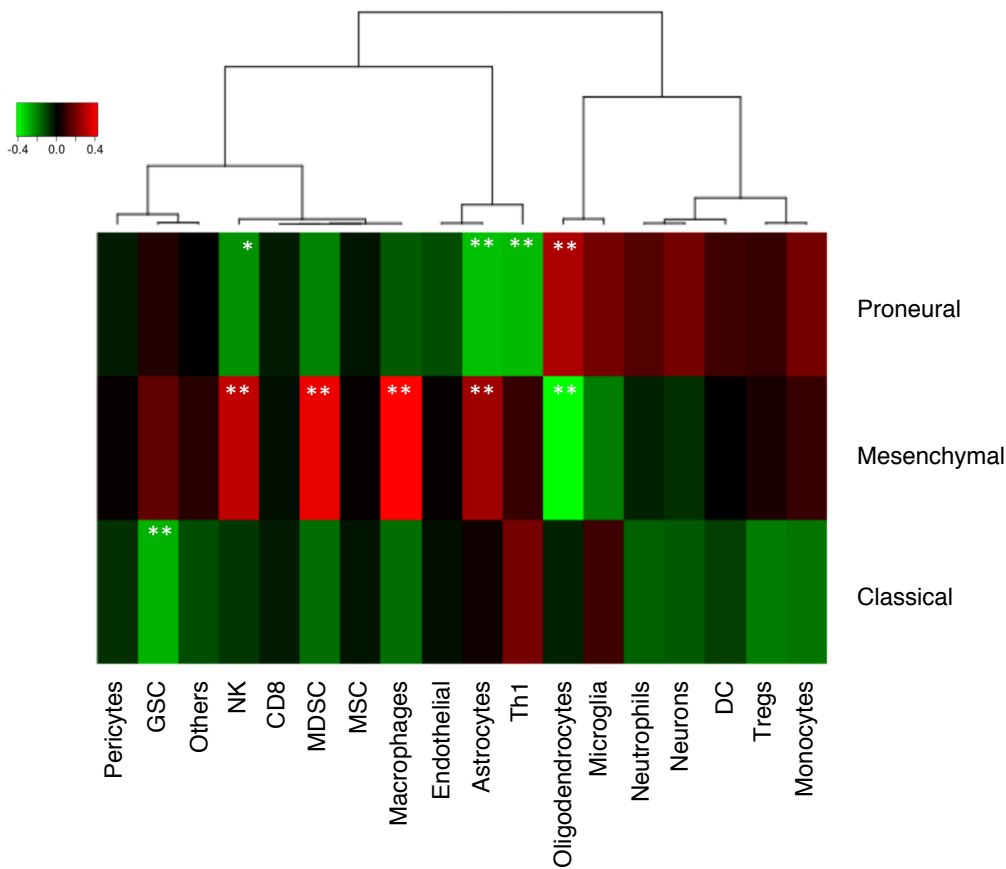

Supplement: giaa109_Supplemental_Figures_and_Tables [file giaa109_supplemental_figures_and_tables.zip › Figure_S3_R1.pdf]

### Figure S4

**A**

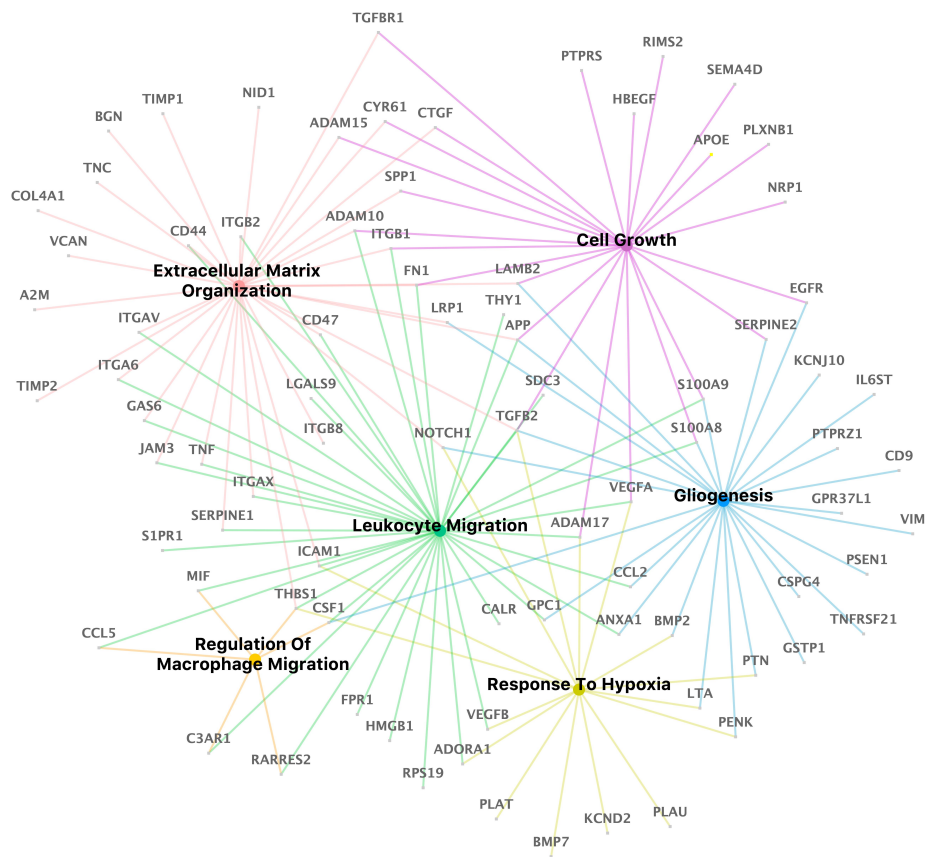

**B**

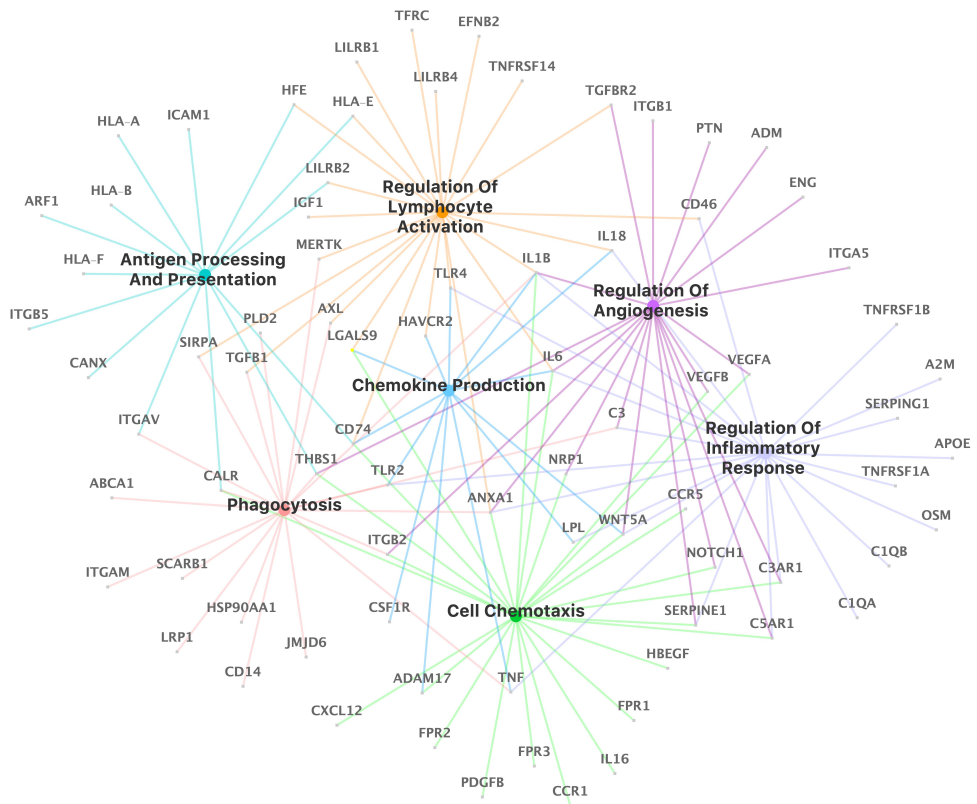

Supplement: giaa109_Supplemental_Figures_and_Tables [file giaa109_supplemental_figures_and_tables.zip › Figure_S4_R1.pdf]

Figure S5

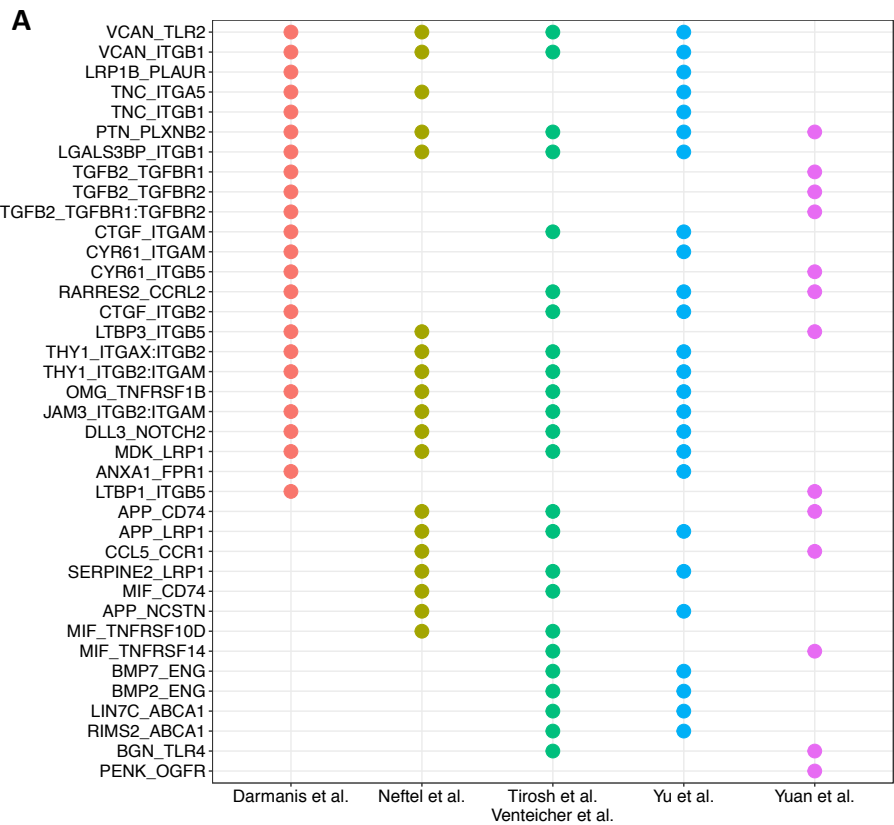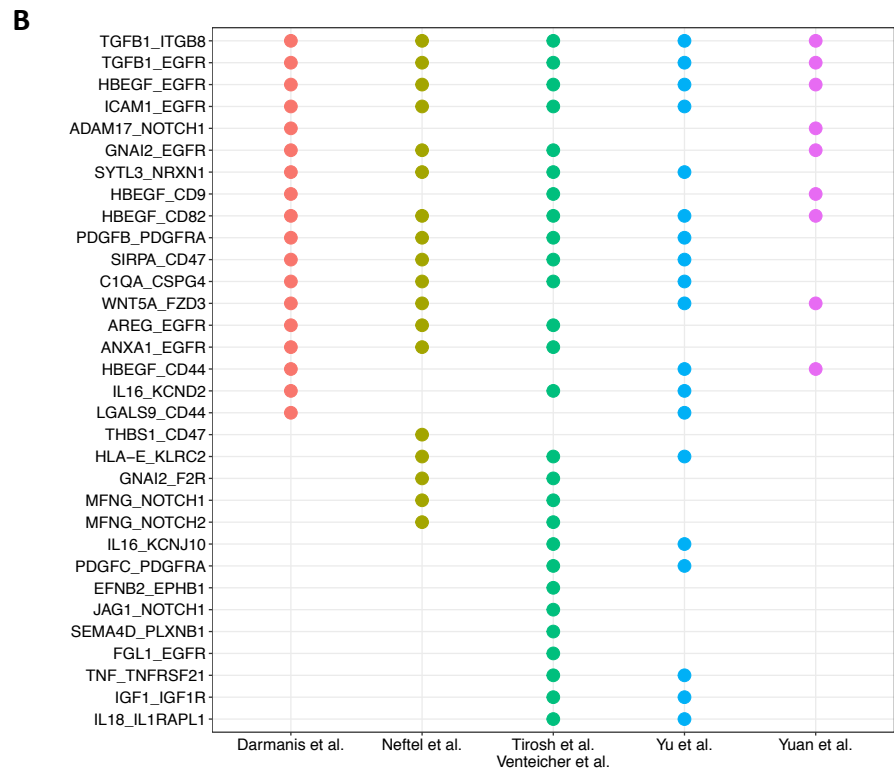

Supplement: giaa109_Supplemental_Figures_and_Tables [file giaa109_supplemental_figures_and_tables.zip › Figure_S5_R1.pdf]

**Figure S6**

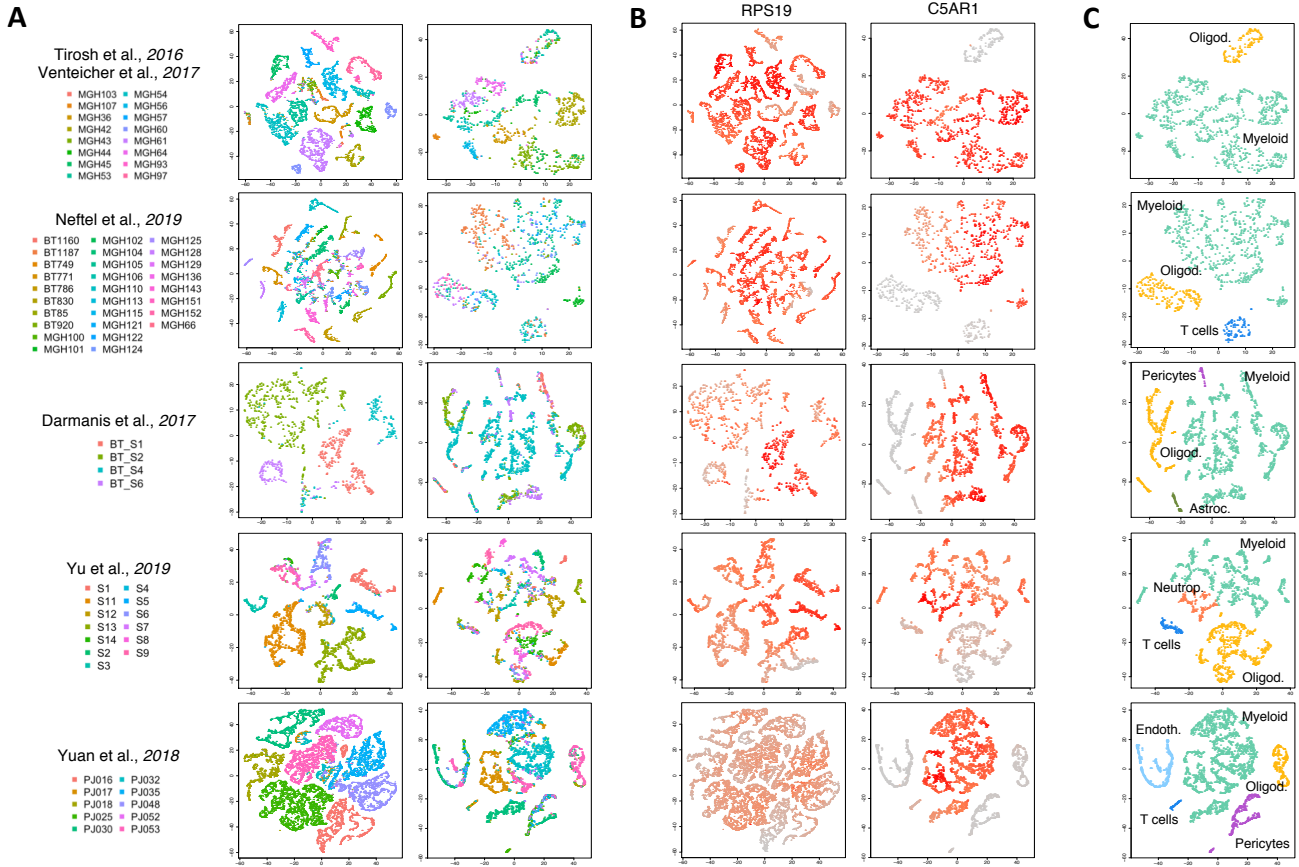

Supplement: giaa109_Supplemental_Figures_and_Tables [file giaa109_supplemental_figures_and_tables.zip › Figure_S6_R1.pdf]

Figure S7

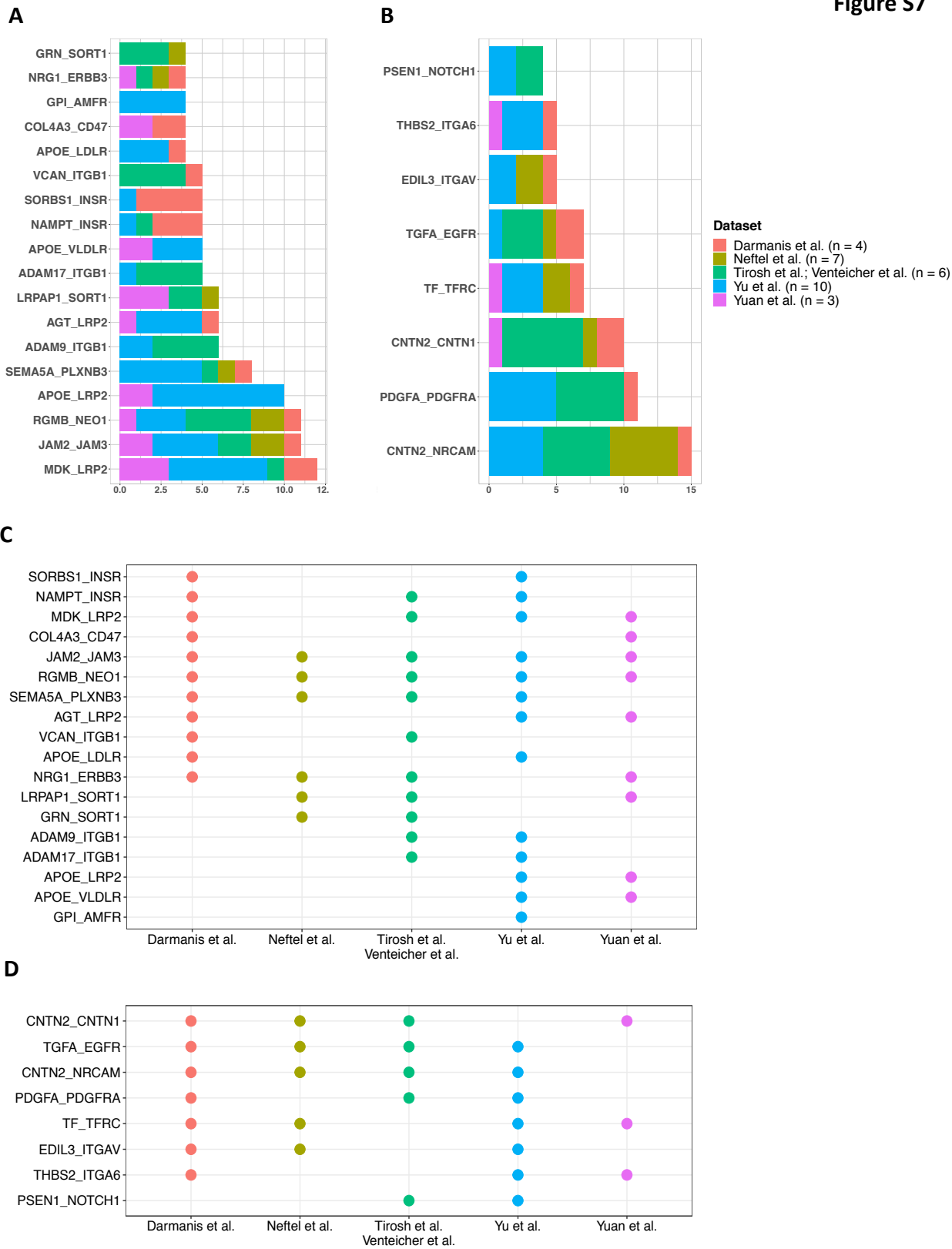

Supplement: giaa109_Supplemental_Figures_and_Tables [file giaa109_supplemental_figures_and_tables.zip › Figure_S7_R1.pdf]

Figure S8

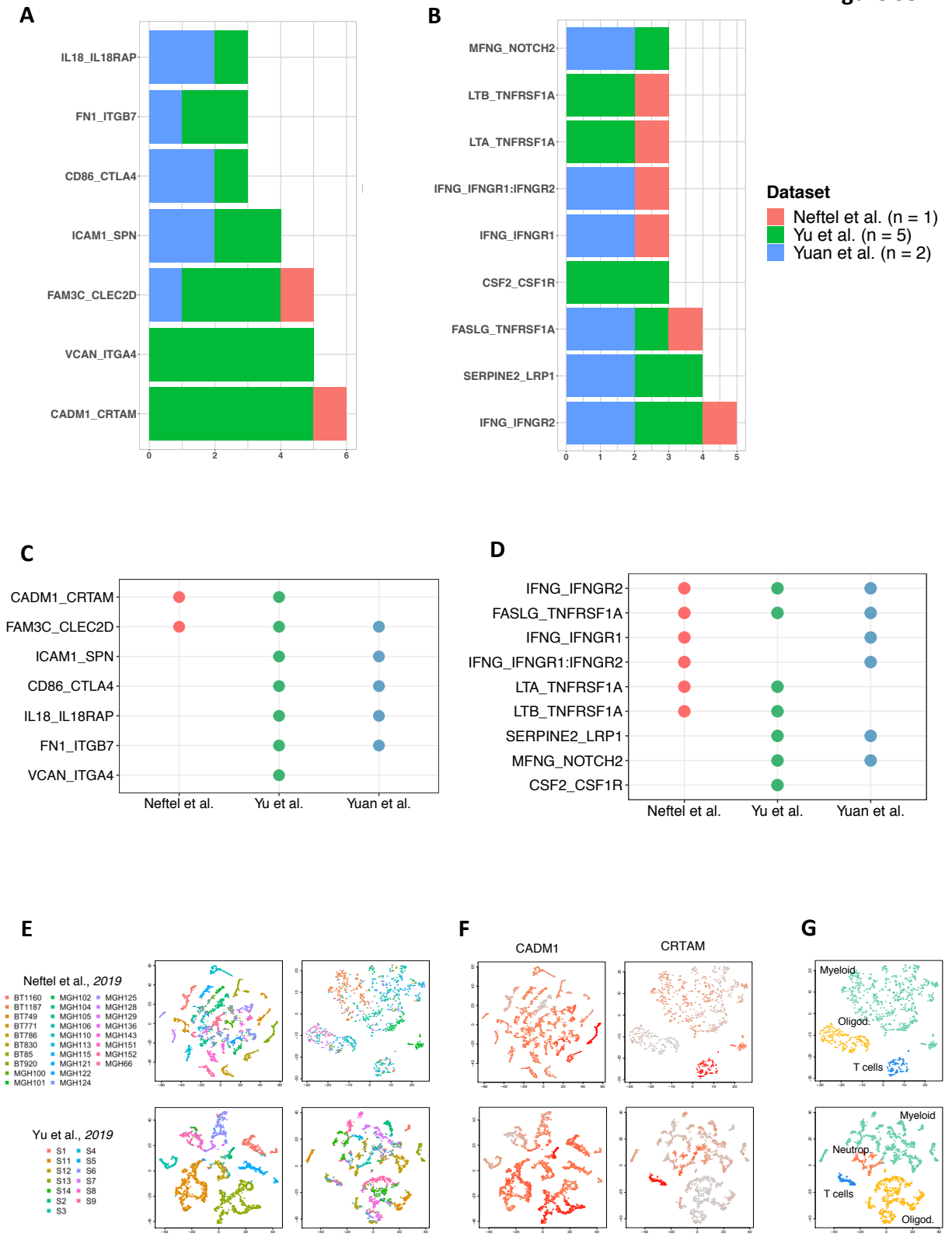

Supplement: giaa109_Supplemental_Figures_and_Tables [file giaa109_supplemental_figures_and_tables.zip › Figure_S8_R1.pdf]

Figure S9

A

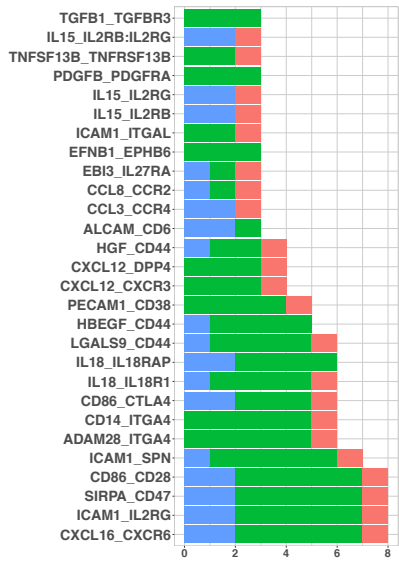

B

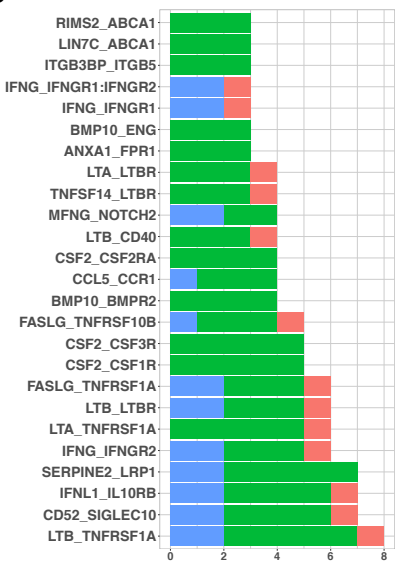

Dataset

Neftel et al. (n = 1)  
Yu et al. (n = 5)  
Yuan et al. (n = 2)

C

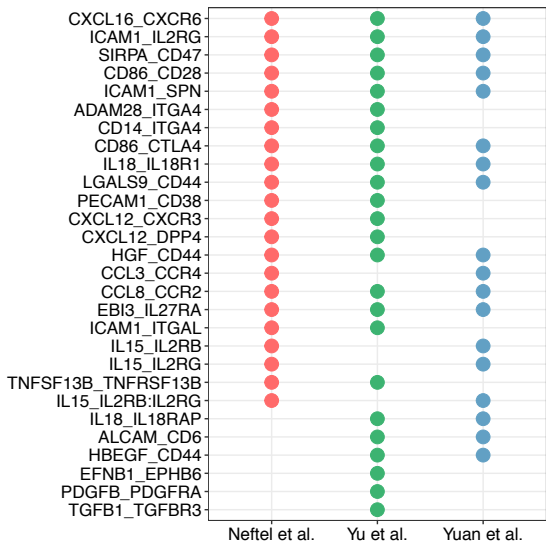

D

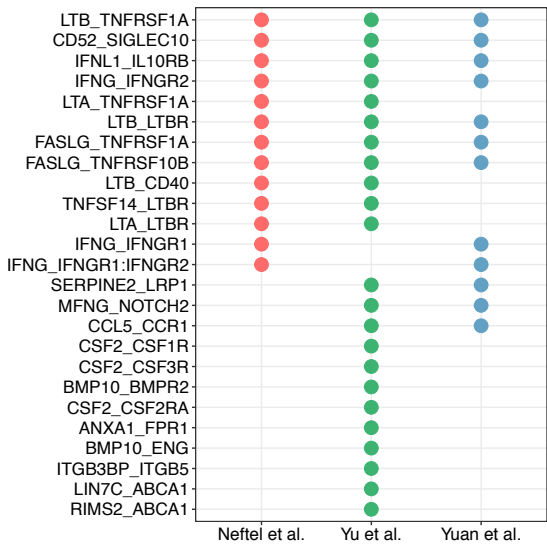

Supplement: giaa109_Supplemental_Figures_and_Tables [file giaa109_supplemental_figures_and_tables.zip › Figure_S9_R1.pdf]
