# Supplementary material for: A map of tumor–host interactions in glioma at single-cell resolution
Source: Gigascience. 2020 Oct 14;9(10):giaa109. doi: 10.1093/gigascience/giaa109 (PMC7645027; doi:10.1093/gigascience/giaa109)
Supplement: giaa109_Response_to_Reviewer_Comments_Original_Submission [file giaa109_response_to_reviewer_comments_original_submission.pdf]

Author's Response To Reviewer Comments

Close

We would like to thank the Editor and the Reviewers for taking the time to critique our manuscript so carefully. We are extremely pleased with their comments as well as their very useful and constructive criticisms and suggestions. With this revision, we have taken careful steps to address the concerns of the Referees and to incorporate their suggestions into the new manuscript. Following are our responses to each of the Reviewers' comments.

Reviewer #1:
In this paper the authors use a large collection of scRNA-seq glioma data to identify cells from hosts which communicate with tumor cells. They identify a large number of interesting interactions specific to subtypes of tumors and various non-neoplastic cells. These interactions would be of great value to the scientific and medical community. It is however not clear how much trust should be put in the identified interactions. The authors greatest tool available to prove this trust is the large number of patients, which is under-utilized for statistical analysis.

The authors make use of a number of rank-based statistics methodology, and in particular use imputed gene expression which is highly likely to produce over-confident expression levels and a large number of false positives.

Major issues

- The most convincing results are those which are replicated between many individual patients. The authors need to quantify these results with some statistical analysis. It appears substantial that an interaction is preserved across e.g. 20 out of 39 patients, or 11 out of 30 patients. But how much more is that than would be expected by chance? The authors can use some form of binomial test to quantify this, with some randomization strategy to identify what the probability of success for the null hypothesis should be.

Response: We thank the reviewer for this interesting suggestion. We agree that the observation of recurrent interaction among patients and among datasets was unexpected and this is our main finding. In order to answer the question about the significance of recurrence, we performed an extensive set of experiments trying to generate a null hypothesis through randomization. Our statistical analysis, as suggested by the reviewer, is based on a binomial test. In order to compute the parameter of the binomial distribution under the null hypothesis, for each cell compartment (myeloid, oligodendrocyte, T-lymphocyte) we first generate a synthetic patient with 1000 cells, 500 from the clusters of malignant cells and 500 from the cluster of non-malignant cells, and then generated 100 random ligand-receptor table by shuffling the rows of the original interaction table. This generates a set of random L-R pairs preserving the overall distribution of the gene expression of the genes involved in the interaction. By applying scTHI to the synthetic patient and the random interaction tables, we compute the expected number of active interactions per patient in the null case. We then use this value as the p parameter of the binomial test. It is worth mentioning that we obtain exactly the same estimate (up to the fourth decimal digit), if, on the contrary, we generate a synthetic interaction table and sample on the patients profiles. For each interaction described in the text we report its significance, in particular tables S4, S6, S8 and S9 were updated with the column of the p-value of the binomial test. The main conclusion is the recurrence of the preserved interactions is highly significant and therefore our results constitute a general map of active cross-talk between cancer cells and microenvironment in glioma that can be exploited in functional follow-up studies.

- Similar to this point, can a similar quantification be made regarding identifying the validated L-R interactions from the Govek et al paper? Is it significant that 3 interactions are present among the top 10 identified interactions?

Response: This is another interesting suggestion that we answered with a simple Fisher’s exact test. We tested 361 interactions and checked whether in the top 10 we have one of the validated interactions of Fig. 4 of the Govek et al. paper for each cell type, obtaining a p-value of 0.0277.

Minor issues

- It is not clear what 'relevant cells' refer to on page 9. Is it how large the fraction of a cell type is? Or how many potential communication partners they appear to have?

Response: Here we intended that a large fraction of cells are shared among patients and datasets. We agree that the term is not truly appropriate and rephrased the corresponding sentence.

- The argument for why the custom developed scTHI score would not suffer from the same issues as the mean expression based scores in previous publications is unclear.

Response: This is an important point that we tried to address in this revision with a new supplementary figure S1 with typical cases where our rank-based score seems to better quantify the properties of the expression of L-R pairs in order to be considered potentially active. As also pointed out in the response to a similar comment raised by reviewer #3, in order to perform a quantitative comparison, there will be the need of a suitable dataset with a ground truth, and this is something that still is not available in this emerging field.

- The authors claim that the use of ranked expression values is more stable than observed counts to compare gene pairs, yet provide no citation nor demonstration.

Response: We agree that this sentence was misleading and has been removed. Indeed we argumented the use of ranked expressions as underlined in the previous response.

- In the discussion section, when writing cell types are 'associated', do the authors mean they have the ability to communicate?

Response: Thanks for the accurate comment. Indeed, we observed that some cell types are particularly represented in tumors with high presence of cell belonging to specific subtypes, such as macrophages for Mesenchymal cells, oligodendrocytes for Proneural cells, and microglia for the cells of the Classical subtype (Figure S3). Nevertheless, we rephrase the unclear sentences.

Reviewer #2:
This work provide a map of the active tumor-host interaction pairs in glioma.The author utilized single cell data developing scTHI to identify the ligand-receptor pairs that modulate the tumor-microenvironment cross-talk in glioma.This is a meaningful and informative study. Moreover, the manuscript is logical and well-written.I think the paper can be accepted directly.

Response: Many thanks for the positive comments.

Reviewer #3:
Caruso et al. developed a novel method named scTHI to identify the cell-to-cell interactions. They applied scTHI to detect the ligand-receptor interactions in six glioma datasets and found some interesting cell-cell communications.

Major:
1. A number of approaches are available for identifying the ligand-receptor interactions, such as CellPhoneDB, PyMINEr, and iTALK. It is intriguing to know the advantages of scTHI compared to other similar methods. Does scTHI have higher sensitivity and/or specificity?

Response: We completely agree with the reviewer that there are a number of approaches already available each with its pros and cons, and a quantitative comparison will be interesting. For example

iTALK comes with excellent visualization tools but is based on average expression, and as we pointed out in the new manuscript with some explicit examples, this can generate several false positives. Moreover, CellPhoneDB is probably the best known method with an excellent ligand-receptor database (that we extend in scTHI) and a web interface. This reviewer will agree that in order to perform a quantitative comparison in terms of sensitivity and/or specificity there will be the need of a suitable dataset with a ground truth, and this is something that still is not available in this emerging field. However we have reported that scTHI is able to detect some of the validated interactions from the paper of Govek et al., but this cannot be considered in no way as a ground truth as this would require that the interactions described by the authors are all and the only active L-R pairs in the considered populations, and there is no reason to not assume that iTALK and CellPhoneDB are not able to detected the ones reported, as indeed they do. The recent paper about the CellPhoneDB protocol (Efermova et al., Nature Protocols 2020) outlines the strength of their work in terms of the quality of the ligand–receptor interaction database, but no quantitative comparison is possible for the time being with the lack of a complete ground truth database. However, in the revised manuscript we have reported in Figure S1, typical exemple cases where our rank-based score seems to better quantify the properties of the expression of L-R pairs in order to be considered potentially active. Overall we can summarize the contributions and strengths of our work as follows:

- scTHI differently from similar approaches takes into account paracrine effects and is able to report L-R pairs specifically expressed in the analyzed subpopulations.
- scTHI score is different from the current approaches as it is based on ranks of gene expression and can generate less false positives with respect to approaches based on average of the gene expression alone
- scTHI is a Bioconductor package and therefore it is integrated with the largest collection of tools for genomics data analysis
- In addition to the development of scTHI, the main contribution of the paper is its application to a large collection of glioma single cell datasets. We have shown that, surprisingly, there are cross-talk partners significantly shared across patients and across dataset. We believe that the reported candidates are a rich set of targets that can be therapeutically exploited in follow-up functional studies.

2. The authors detected unexpected cross-talk partners that were highly conserved across different datasets. This could be interesting. Can the author add a figure to show the similarities and differences of cell-cell interactions among distinct datasets?

Response: As also mentioned by reviewer #1, our main results is the identification of recurrent crosstalk across different patients and different datasets. With this revision we have included a statistical analysis to characterize the significance of the recurrent cross-talks as explained above, the tables S4, S6, S8 and S9 were updated with the column of the p-value of the statistical adopted test, and the described interaction were characterized by their significance of recurrence. Moreover, in order to accommodate the specific request of this reviewer, we have amended figures S5, S7, S8, and S9 that now show, for every significant interaction, the datasets where they were detected.

3. Table 1 showed six glioma datasets used in this study. Some single-cell RNA-seq (scRNA-seq) technologies can sequence the full-length of transcripts (e.g. Smart-seq2), while some others can only capture the 3'/5'-end of the transcripts (e.g. 10X Genomics). These two different types of protocols may influence the data analysis. It would be helpful to add one more column in table 1 to indicate the type of scRNA-seq strategy used in the original studies.

Response: The reviewer correctly noticed that we used datasets from different platforms, and we specified the platforms in Table 1 of the revised manuscript. However we underline that our analysis is performed per patient, and we do not integrate datasets from different technologies. The reviewer will agree that the fact that there are several cross-talk partners across multiple datasets from different technologies corroborates our findings.

Minor:

1. Figure 2 showed the cell-type classification in glioma, but the six datasets used in this study were not mentioned. Can the authors explain more about this figure?

Response: We explained in the text that the percentages of specific cell compartments were computed using the datasets where the cells did not undergo any gating or selection strategy (specifically: Yu et

al.; Yuan et al.; Darmanis et al.). In the revised manuscript we report in table S3 the breakdown of the cell number of cells for each compartment and dataset.

2. It is recommended to use the white background for Figure 2 and Figure 3.

Response: Many thanks for the suggestion, we have updated Figure 2 and 3 as advised.

Close
