# Supplementary material for: A map of tumor–host interactions in glioma at single-cell resolution
Source: Gigascience. 2020 Oct 14;9(10):giaa109. doi: 10.1093/gigascience/giaa109 (PMC7645027; doi:10.1093/gigascience/giaa109)
Supplement: giaa109_Reviewer_1_Report_Original_Submission — Valentine Svensson -- 5/4/2020 Reviewed [file giaa109_reviewer_1_report_original_submission.pdf]

Reviewer Report

Title: A MAP of tumor-host interactions in glioma at single cell resolution

Version: Original Submission **Date:** 5/4/2020

Reviewer name: Valentine Svensson

Reviewer Comments to Author:

Review
In this paper the authors use a large collection of scRNA-seq glioma data to identify cells from hosts which communicate with tumor cells. They identify a large number of interesting interactions specific to subtypes of tumors and various non-neoplastic cells. These interactions would be of great value to the scientific and medical community. It is however not clear how much trust should be put in the identified interactions. The authors greatest tool available to prove this trust is the large number of patients, which is under-utilized for statistical analysis.
The authors make use of a number of rank-based statistics methodology, and in particular use imputed gene expression which is highly likely to produce over-confident expression levels and a large number of false positives.
Major issues
- The most convincing results are those which are replicated between many individual patients. The authors need to quantify these results with some statistical analysis. It appears substantial that an interaction is preserved across e.g. 20 out of 39 patients, or 11 out of 30 patients. But how much more is that than would be expected by chance? The authors can use some form of binomial test to quantify this, with some randomization strategy to identify what the probability of success for the null hypothesis should be.
- Similar to this point, can a similar quantification be made regarding identifying the validated L-R interactions from the Govek et al paper? Is it significant that 3 interactions are present among the top 10 identified interactions?
Minor issues
- It is not clear what 'relevant cells' refer to on page 9. Is it how large the fraction of a cell type is? Or how many potential communication partners they appear to have?
- The argument for why the custom developed scTHI score would not suffer from the same issues as the mean expression based scores in previous publications is unclear.
- The authors claim that the use of ranked expression values is more stable than observed counts to compare gene pairs, yet provide no citation nor demonstration.
- In the discussion section, when writing cell types are 'associated', do the authors mean they have the ability to communicate?

Methods

Are the methods appropriate to the aims of the study, are they well described, and are necessary controls included? Choose an item.

Conclusions

Are the conclusions adequately supported by the data shown? Choose an item.

Reporting Standards

Does the manuscript adhere to the journal's guidelines on minimum standards of reporting? Choose an item.

Choose an item.

Statistics

Are you able to assess all statistics in the manuscript, including the appropriateness of statistical tests used? Choose an item.

Quality of Written English

Please indicate the quality of language in the manuscript: Choose an item.

Declaration of Competing Interests

Please complete a declaration of competing interests, considering the following questions:

- Have you in the past five years received reimbursements, fees, funding, or salary from an organisation that may in any way gain or lose financially from the publication of this manuscript, either now or in the future?
- Do you hold any stocks or shares in an organisation that may in any way gain or lose financially from the publication of this manuscript, either now or in the future?
- Do you hold or are you currently applying for any patents relating to the content of the manuscript?
- Have you received reimbursements, fees, funding, or salary from an organization that holds or has applied for patents relating to the content of the manuscript?
- Do you have any other financial competing interests?
- Do you have any non-financial competing interests in relation to this paper?

If you can answer no to all of the above, write 'I declare that I have no competing interests' below. If your reply is yes to any, please give details below.

I declare that I have no competing interests

I agree to the open peer review policy of the journal. I understand that my name will be included on my report to the authors and, if the manuscript is accepted for publication, my named report including any attachments I upload will be posted on the website along with the authors' responses. I agree for my

report to be made available under an Open Access Creative Commons CC-BY license (http://creativecommons.org/licenses/by/4.0/). I understand that any comments which I do not wish to be included in my named report can be included as confidential comments to the editors, which will not be published.

Choose an item.

To further support our reviewers, we have joined with Publons, where you can gain additional credit to further highlight your hard work (see: https://publons.com/journal/530/gigascience). On publication of this paper, your review will be automatically added to Publons, you can then choose whether or not to claim your Publons credit. I understand this statement.

Yes Choose an item.
