# Supplementary material for: A map of tumor–host interactions in glioma at single-cell resolution
Source: Gigascience. 2020 Oct 14;9(10):giaa109. doi: 10.1093/gigascience/giaa109 (PMC7645027; doi:10.1093/gigascience/giaa109)
Supplement: giaa109_Reviewer_3_Report_Original_Submission — Geng Chen -- 6/3/2020 Reviewed [file giaa109_reviewer_3_report_original_submission.pdf]

Reviewer Report

Title: A MAP of tumor-host interactions in glioma at single cell resolution

Version: Original Submission **Date:** 6/3/2020

Reviewer name: Geng Chen

Reviewer Comments to Author:

Caruso et al. developed a novel method named scTHI to identify the cell-to-cell interactions. They applied scTHI to detect the ligand-receptor interactions in six glioma datasets and found some interesting cell-cell communications.

Major:

1. A number of approaches are available for identifying the ligand-receptor interactions, such as CellPhoneDB, PyMINEr, and iTALK. It is intriguing to know the advantages of scTHI compared to other similar methods. Does scTHI have higher sensitivity and/or specificity?
2. The authors detected unexpected cross-talk partners that were highly conserved across different datasets. This could be interesting. Can the author add a figure to show the similarities and differences of cell-cell interactions among distinct datasets?
3. Table 1 showed six glioma datasets used in this study. Some single-cell RNA-seq (scRNA-seq) technologies can sequence the full-length of transcripts (e.g. Smart-seq2), while some others can only capture the 3'/5'-end of the transcripts (e.g. 10X Genomics). These two different types of protocols may influence the data analysis. It would be helpful to add one more column in table 1 to indicate the type of scRNA-seq strategy used in the original studies.

Minor:

1. Figure 2 showed the cell-type classification in glioma, but the six datasets used in this study were not mentioned. Can the authors explain more about this figure?
2. It is recommended to use the white background for Figure 2 and Figure 3.

Methods

Are the methods appropriate to the aims of the study, are they well described, and are necessary controls included? Choose an item.

Conclusions

Are the conclusions adequately supported by the data shown? Choose an item.

Reporting Standards

Does the manuscript adhere to the journal's guidelines on [minimum standards of reporting?]() Choose an item.

Choose an item.

Statistics

Are you able to assess all statistics in the manuscript, including the appropriateness of statistical tests used? Choose an item.

Quality of Written English

Please indicate the quality of language in the manuscript: Choose an item.

Declaration of Competing Interests

Please complete a declaration of competing interests, considering the following questions:

- Have you in the past five years received reimbursements, fees, funding, or salary from an organisation that may in any way gain or lose financially from the publication of this manuscript, either now or in the future?
- Do you hold any stocks or shares in an organisation that may in any way gain or lose financially from the publication of this manuscript, either now or in the future?
- Do you hold or are you currently applying for any patents relating to the content of the manuscript?
- Have you received reimbursements, fees, funding, or salary from an organization that holds or has applied for patents relating to the content of the manuscript?
- Do you have any other financial competing interests?
- Do you have any non-financial competing interests in relation to this paper?

If you can answer no to all of the above, write 'I declare that I have no competing interests' below. If your reply is yes to any, please give details below.

I declare that I have no competing interests.

I agree to the open peer review policy of the journal. I understand that my name will be included on my report to the authors and, if the manuscript is accepted for publication, my named report including any attachments I upload will be posted on the website along with the authors' responses. I agree for my report to be made available under an Open Access Creative Commons CC-BY license (http://creativecommons.org/licenses/by/4.0/). I understand that any comments which I do not wish to be included in my named report can be included as confidential comments to the editors, which will not be published.

Choose an item.

To further support our reviewers, we have joined with Publons, where you can gain additional credit to further highlight your hard work (see: https://publons.com/journal/530/gigascience). On publication of this paper, your review will be automatically added to Publons, you can then choose whether or not to claim your Publons credit. I understand this statement.

Yes Choose an item.
